# Polymorphism and Multi-Component Crystal Formation of GABA and Gabapentin

**DOI:** 10.3390/pharmaceutics15092299

**Published:** 2023-09-10

**Authors:** Daniel Komisarek, Fulya Demirbas, Takin Haj Hassani Sohi, Klaus Merz, Carsten Schauerte, Vera Vasylyeva

**Affiliations:** 1 Laboratory for Crystal Engineering, Department of Inorganic and Structural Chemistry 1, Heinrich-Heine-University Dueseldorf, Universitaetsstraße 1, 40225 Duesseldorf, Germany; daniel.komisarek@hhu.de (D.K.);; 2 Inorganic Chemistry 1, Ruhr-University Bochum, Universitaetstrasse 150, 44801 Bochum, Germany; 3 SOLID-CHEM GmbH, Universitaetstrasse 136, 44799 Bochum, Germany

**Keywords:** GABA, gabapentin, API, non-covalent interactions, pharmaceutical crystal engineering, hydrogen bond, polymorphism, multicomponent crystals, crystallization

## Abstract

This study exploits the polymorphism and multi-component crystal formation of γ-amino butanoic acid (GABA) and its pharmaceutically active derivative, gabapentin. Two polymorphs of GABA and both polymorphs of gabapentin are structurally revisited, together with gabapentin monohydrate. Hereby, GABA form **II** is only accessible under special conditions using additives, whereas gabapentin converts to the monohydrate even in the presence of trace amounts of water. Different accessibilities and phase stabilities of these phases are still not fully clarified. Thus, indicators of phase stability are discussed involving intermolecular interactions, molecular conformations, and crystallization environment. Calculated lattice energy differences for polymorphs reveal their similar stability. Quantification of the hydrogen bond strengths with the atoms-in-molecules (AIM) model in conjunction with non-covalent interaction (NCI) plots also shows similar hydrogen bond binding energy values for all polymorphs. We demonstrate that differences in the interacting modes, in an interplay with the intermolecular repulsion, allow the formation of the desired phase under different crystallization environments. Salts and co-crystals of GABA and gabapentin with fumaric as well as succinic acid further serve as models to highlight how strongly HBs act as the motif-directing force in the solid-phase GABA-analogs. Six novel multi-component entities were synthesized, and structural and computational analysis was performed: GABA fumarate (2:1); two gabapentin fumarates (2:1) and (1:1); two GABA succinates (2:1) and (1:1); and a gabapentin:succinic acid co-crystal. Energetically highly attractive carboxyl/carboxylate interaction overcomes other factors and dominates the multi-component phase formation. Decisive commonalities in the crystallization behavior of zwitterionic GABA-derivatives are discussed, which show how they can and should be understood as a whole for possible related future products.

## 1. Introduction

Understanding the solid phase remains a topic of high interest in crystal engineering for many applications, and co-crystallization and polymorphism control are in demand for the optimization of materials such as polymers [1], batteries [2], luminescent compounds [3], or high-energy substances [4,5]. However, the production of a reliable phase is a core objective, especially in pharmaceutics. Bernstein and Dunitz’s work on disappearing polymorphs in 1995, as well as Bernstein’s 2015 follow-up on the same topic, could be considered classics in the literature on crystal engineering to this point [6,7]. Still, more recent research has been conducted, providing either a broad overview [8,9] or a specific outlook concerning single substances [10,11]. Additionally, emerging as well as established techniques in crystal synthesis control are being continuously updated [12,13,14]. With growing accessibility and increasingly higher performances of computational methods, these are becoming more prevalent, refined, and numerous in their uses. Examples include approachable programs like T. Lus multiwfn, which enables atoms-in-molecules (AIM) analyses as well as non-covalent interaction (NCI) plotting and other useful applications [15,16,17]. Furthermore, many tools are implemented into the popular Cambridge Crystallographic Data Centre’s (CCDC) Mercury software, which provides, among numerous other uses, applications such as polymorph or co-crystal screening [18]. Even Density Functional Theory (DFT)-based programs like Giannozzi’s Quantum Espresso (QE) or the popular Gaussian software show increased usage and performance [19,20]. Recent advancements in computational crystal structure evaluations allow insights into the basics of the crystallization process [21,22,23,24] and in-depth studies specifically aimed to describe properties of singular compounds of interest [25,26,27,28].

A specific compound class that attracts interest due to its structural behavior is that of small, zwitterionic amino acids. A prominent example, glycine, which is the simplest amino acid, has gained some notoriety over the years for its unpredictable polymorphism and crystallization behavior, being called ‘The Gift that Keeps on Giving’ by Boldyreva in 2021 [28,29,30,31,32,33,34,35]. Structurally related and not less interesting, γ-amino butanoic acid (GABA) also exhibits polymorphic behavior; however, it has received considerably less attention. A Scifinder-n search (November 2022) of the terms ‘Glycine AND crystal’ compared to ‘GABA AND crystal’ led to 12,331 and 858 results, respectively. GABA, an important nonessential amino acid with a variety of GABA receptors named after it, acts as a neurotransmitter inhibitor and is linked to sleep and stress relief [36,37,38,39].

The polymorphism of GABA has been sporadically structurally characterized; however, there are still gaps in understanding despite several reported attempts to control and clarify its formation. Single crystal structure determination of both known polymorphs **I**- and **II**-GABA reaches back to the 1970s and 1990s [40,41]. A monoclinic polymorph **I** (*gauche* conformer), a thermodynamic, commercially available form crystallizes readily from an aqueous solution, and a tetragonal form **II** (*trans* conformer) is stated to be kinetic, elusive modification. Recently, Wang et al. found strong evidence of the existence of a polymorph **III**-GABA in their 2020 contribution, where authors explain the polymorph formation to be dependent on the dihedral angle in different GABA modifications, in congruence with the interaction motifs [42]. Interestingly, Vasceq et al. showed polymorphs of GABA to perform different permeabilities in crossing the blood−brain barrier, where the metastable **II**-GABA modification exhibits higher bioactivity [43]. Wang et al. reported strategies of additive-induced and liquid-assisted mechanochemical GABA polymorph control [42,44], followed by Lamkowski et al., who discussed the pH influences on the stabilization of **II**-GABA in 2022 [45]. A plethora of pharmaceutically active ingredients (APIs) are derived from GABA, such as pregabalin, phenibut, baclofen, and gabapentin, which have all been discussed regarding their crystal structure over time [46,47,48,49,50,51,52,53,54,55,56,57]. Gabapentin stands out as a reliable medication, popularly used for nearly 30 years. Its primary applications include anti-epileptic capabilities as well as uses in the treatment of neuropathic and inflammatory pain [58,59,60]. Its crystallization, polymorphism [61,62,63,64,65,66], and co-crystallization behavior have been widely studied over time [67,68,69,70]. Lastly, in 2022, Liu et al. proposed that the polymorphic phase transfer can be explained by careful analysis of gabapentin conformations in solvent environments [71]. Both polymorphs of gabapentin (form **II** and form **IV**) remain stable once crystallized from the respective solvents. However, gabapentin readily converts to its hydrate modification (**I**-gabapentin) when water is present during the crystallization process. To sum up, previous factors like crystallization conditions and molecular conformations, as well as energetic contributions on a molecular level, were taken into consideration to describe phase stability for GABA and gabapentin, mainly in solution or in a gas phase [24,42,43,44,45,61,63,64,65,66,71,72].

**Scheme 1:**
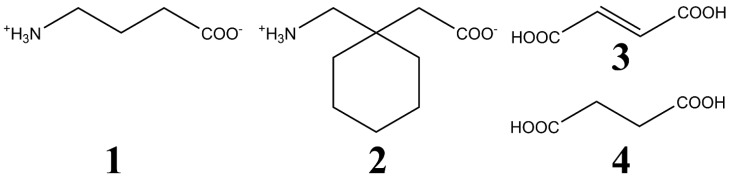
Overview of the examined compounds. **1** = γ-amino butanoic acid (GABA), **2** = gabapentin, **3** = fumaric acid, **4** = succinic acid.

A careful examination of the crystal architecture from an energetic perspective on intermolecular interaction strengths and lattice energy differences performed in this study can significantly contribute to the previously held discussions on the present topic. Furthermore, analysis of crystalline multi-component systems as salts or co-crystals can help to clarify the driving forces for single and multi-component phase formation. Thus, in order to better understand how and why the crystallization processes occur, different crystalline embodiments of GABA (**1**) and gabapentin (**2**) with fumaric (**3**) and succinic acid (**4**) (Scheme 1) were synthesized as single and multi-component entities, followed by their structural and computational examination. The respective phases are polymorphic modifications **I**-**1** and **II**-**1** (*P*2_1_/*c* and *I*4_1_*cd*), **II**-**2** and **IV**-**2** (*P*2_1_/*c* and *C*2/*c*), a gabapentin monohydrate (**I**-gabapentin), as well as five salts and a co-crystal: GABA fumarate (2:1, **1**-**3**), two gabapentin fumarates (2:1, **2**-**3a**) and (1:1, **2**-**3b**), two GABA succinates (2:1, **1**-**4a**) and (1:1, **1**-**4b**) and a gabapentin:succinic acid co-crystal (**2**-**4**). The nomenclature for GABA and gabapentin single phases is based on the works by Liu et al. and Lamkowski et al. and their respective 2022 publications [45,71].

The AIM model is used to calculate energy values for the direct sphere of interaction of each crystallographically independent molecule in all investigated species. NCI-based scatterplots were generated for these spheres of interactions and single molecule/molecule interactions to understand the influence of strong hydrogen bonds (HBs) and molecular repulsion on the crystallization product. Additionally, the molecular dihedral angles on the GABA moieties were compared. Influences on phase stability were evaluated under consideration of experimental crystal growth conditions, energetic differences in polymorphs and energetic contributions of strong and weak hydrogen bonds, and molecular conformation of the GABA moiety in each species. 

## 2. Materials and Methods

Synthesis:

Polymorphs and Hydrate of GABA and gabapentin were synthesized via slow evaporation of the solvent from different solution environments: **I**-GABA and gabapentin • H_2_O were grown from aqueous solutions, **II**-gabapentin was received from methanol, **II**-GABA and **IV**-gabapentin were obtained from aqueous solution with 2 vol% acetic acid as an additive. A partial interconversion of pure **II**-**2** (85.5 mg, 0.5 mmol) to its hydrate **I**-**2** occurred during the neat mechanochemical grinding for 30 min at 25 Hz in a ball-mill Retsch MM400 with three ZrO_2_ balls of 10 mm diameter at ambient conditions. Milling vessels were not heat-dried prior, and no air exclusion was performed during the grinding experiment.

Salts and Co-Crystal were all synthesized by slow evaporation of solvent from aqueous solution. The amount of the solvent varied from 1 to 5 mL to completely dissolve the respective sample. The 1:1 forms were prepared by dissolving equimolar amounts of either API with a dicarboxylic acid; the 2:1 forms with double the amount of API. The following measures were used: GABA fumarate (2:1) (**1**-**3**) with 206 mg (2 mmol) of **1** and 116 mg (1 mmol) of **3**; gabapentin fumarate (2:1) (**2**-**3a**) with 342 mg (2 mmol) of **2** and 116 mg (1 mmol) of **3**; gabapentin fumarate (1:1) (**2**-**3b**) with 171 mg (1 mmol) of **2** and 116 mg (1 mmol) of **3**; GABA succinate (2:1) (**1**-**4a**) 206 mg (2 mmol) of **1** and 118 mg (1 mmol) of **4**; GABA succinate (1:1) (**1**-**4b**) 103 mg (1 mmol) of **1** and 118 mg (1 mmol) of **4**; gabapentin:succinic acid (2:1) (**2**-**4**) with 342 mg (2 mmol) of **2** and 118 mg (1 mmol) of **4**.

Characterization:

PXRD measurements were conducted on a Rigaku Miniflex diffractometer in θ/2θ geometry at ambient temperature using Cu-Kα radiation (λ = 1.54182 Å).

SCXRD measurements were conducted by choosing suitable crystals from a sample and mounting them under oil. Diffraction data were collected using a Rigaku Synergy S diffractometer with Hybrid Pixel Arrow detector with Cu-Kα radiation (λ = 1.54182 Å) at 100 K, or in the case of **II**-GABA, a Bruker Apex Duo with a Kappa geometry and an APEX-II CCD area detector at 140 K. In each case, a colorless plate-shaped crystal was measured. Data reduction and absorption correction were performed using CrysAlisPRO v. 42 software, with numerical absorption correction based on Gaussian integration over a multifaceted crystal model and empirical absorption correction with spherical harmonics, implemented in SCALE3 ABSPACK scaling algorithm [73]. Structure analysis was performed through direct methods (SHELXT-2015), full-matrix least-squares refinements on F2 were performed using the SHELXL2017/1 program package, and structure solution and refinements were executed using Olex2-1.5 software [74,75,76]. Hydrogen atoms were freely refined except for C-H hydrogens in GABA-succinates (1:1) and (2:1), where the following atomic displacement parameters were used: Uiso(HCH) = 1.2 Ueq. Furthermore, the O1-H1 distance was fixed in GABA-succinate 1:1 at 0.9 Å with σ of 0.09 Å. The proton position could not be decisively determined by single crystal X-ray analysis, and thus, the more likely variant (deprotonation of succinic acid, protonation of GABA) based on similar systems was chosen. Lastly, a disorder is present on gabapentin-fumarate (2:1). A carboxyl oxygen O2 on gabapentin is split over two positions parts O2A (Occu: 0.58) and O2B (Occu: 0.42). For calculations, a version of the file in which the disorder was not resolved was used. CCDC numbers: 2240263-2240273.

FT-IR measurements were conducted on a Bruker Tensor 27 Fourier transformed IR spectrometer in attenuated total reflectance mode in the range of 4000 cm^−1^ to 400 cm^−1^.

Computational methods:

Energy calculations for the lattice energy differences were executed with Quantum Espresso (QE) PWSCF v. 6.6 with the Perdew–Burke–Ernzerhof (PBE) model to calculate lattice energy differences between the polymorphs of GABA and gabapentin [20]. The PBEsol basis set was used for atomic pseudopotentials. Our approach described in a previous publication was applied [50]. Calculations of the wave functions for further processing in multiwfn were performed with Gaussian v. 16 on the B3LYP level of theory with the def2-TZVP basis set [19]. AIM and scatterplot analyses were conducted with multiwfn v. 3.8, and figures of NCI and the scatterplots were prepared in *VMD* v. 1.9.4 and gnuplot v. 5.4, respectively [15,77]. AIM analysis was applied for molecular coordinates received from the single crystal structures. Energy values were calculated for every distinct hydrogen bond between each crystallographically independent molecule in the crystal lattices of the investigated compounds. Interaction energies for charged and uncharged hydrogen bonds were calculated based on the model proposed by Emamian et al. in 2019 [17].

Additional software used includes Mercury 2022.3.0 for structural depictions based on received .cif files and calculation of torsions [18] and PLATON for hydrogen bond analysis [78].

Chemicals were obtained from the following suppliers: GABA (≥99%) J&K Scientific, gabapentin (>98%), abcr, succinic acid (99%), and fumaric acid (99%) TCI. Purified water and acetic acid (p.a.) were used as solvents. All chemicals were used without further purification.

## 3. Results and Discussion

### 3.1. Polymorphs of GABA and Gabapentin

To evaluate the phase stability in polymorphs of **1** and **2**, three common discussion points shall be investigated: (i) differences in lattice energy, (ii) molecular conformations in dependence of the crystallization environment and in the final product, and (iii) intermolecular interactions in the crystal lattices of the various polymorphic modifications. First, the relative differences in lattice energies (ΔE_lat_) between polymorphs **I**-**1** and **II**-**1**, as well as **II**-**2** and **IV**-**2**, are investigated. These were calculated by applying geometry optimization through QE on the recorded crystal structures of these compounds. Subsequently, an energy value for the ideal solid-state (E_iss_) of each system was received. By adjusting such E_iss_ values for the number of formula units in the unit cell (Z) and subtraction according to Equation (1), ΔE_lat_ values are received.

(1) $$\Delta E_{lat}=\frac{E_{iss1}}{Z_{1}}-\frac{E_{iss2}}{Z_{2}}$$

Evaluation of the lattice energy differences shows small differences between the modifications, which presumes comparable stability for both **1** and **2** phases, respectively. Polymorph **I**-**1** is more stable compared to form **II**-**1** only by −1.94 kJ mol^−1,^ and form **II** of **2** is more stable than **IV**-**2** by −3.49 kJ mol^−1^. The obtained ΔE_lat_ values are in a typical range for polymorphic substances, as determined by Nyman et al. in 2015 [79]. Now, if lattice energies were the only indicator of phase stability, **II**-**1** and **IV**-**2** should easily undergo phase transitions to **I**-**1** and **II**-**2**, respectively. Indeed, a monotropic phase transition partially occurs by sublimation of **I**-GABA [43], but crystallization of this form from an aqueous solution with acetic acid or by liquid-assisted grinding with acetic acid leads to a stable **II**-**1** product [42,45]. The **2** polymorphs can be obtained from different solvents, but influences such as temperature treatment or mechanical stress can also induce an enantiotropic solid–solid phase transition [61,62,63]. However, the minor lattice energy difference between **I**-**1** and **II**-**1** does not explain why polymorph **II**-**1** is elusive and requires special crystallization conditions. On the contrary, if lattice energies were solely responsible for the phase stability and accessibility, **IV**-**2** should be less accessible than **II**-**1** given a larger ΔE_lat_. 

**Figure 1:**
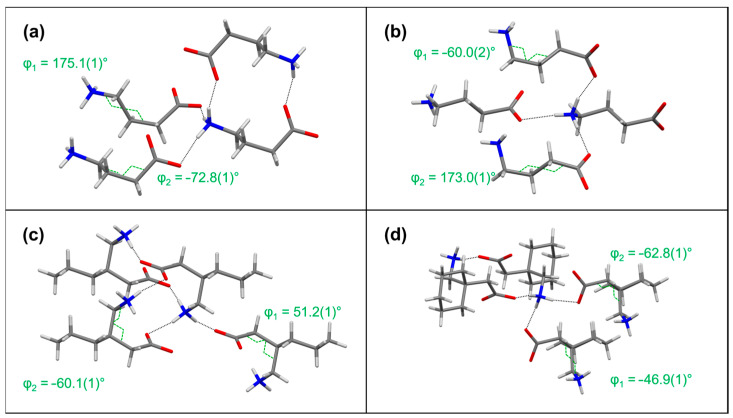
Torsion angles φ1 (N1-C4-C3-C2) and φ2 (C1-C2-C3-C4) as well as characteristic HBs in polymorphs (**a**) **I**-**1**, (**b**) **II**-**1**, (**c**) **II**-**2**, and (**d**) **IV**-**2**.

On the other hand, conformational changes in GABA moieties of **1** and **2** have been connected to their stability in different crystallization environments. To exploit whether additives have a similar influence on the molecular conformation of **2**, the less stable **IV**-**2** was also produced from an aqueous solution with acetic acid. It remains stable over time once synthesized via this method, similar to the **II**-**1** form. Additionally, to characterize GABA conformations in **1** and **2**, modifications in the torsion angles φ_1_ (N1-C4-C3-C2) and φ_2_ (C1-C2-C3-C4) are analyzed along with characteristic HBs (Figure 1). Although both forms **I**-**1** and **II**-**1** have an eclipse conformation in the solid state, φ_1_ and φ_2_ values differ and are inverted with respect to each other. Song et al. reported that neither conformation is especially favorable for dissolved zwitterionic **1** in an aqueous environment, but the state of **I**-**1** can be considered more beneficial compared to **II**-**1** [72]. This is in accordance with our experimental observations and ΔE_lat_ calculations. For gabapentin polymorphs, Liu and colleagues investigated conformational changes in the GABA moieties and cyclohexyl-residue during the nucleation and crystal growth process and explained how saturation and solvent environment favor or disfavor their formation [71]. Thus, the formation process of the received form of **1** or **2** is significantly impacted by the crystallization environment via stabilization of a conformational state that does not necessarily lead to the most stable solid modification.

The question remains: Which factors lead to the higher energetic stabilization of form **I**-**1** and **II**-**2** compared to **II**-**1** and **IV**-**2**, respectively? To identify factors of stability in the solid state, it is necessary to understand the intermolecular interaction motifs. Strong charge-assisted HBs stemming from the zwitterionic nature of all systems are the main occurring attractive force. In each compound, three distinct HBs are formed that show very similar lengths and angles with donor–acceptor distances between 2.7 and 2.8 Å and angles between 158° and 179° (Table 1). To quantify these observations, AIM analyses were conducted on the **1** and **2** polymorphs, and the model established by Emamian et al. in 2019 was used to calculate intermolecular bond energies (E_bond_) for the occurring HBs [17]. Calculated energies correspond to the strong HBs. Each phase quantifies two HBs with E_bond_ values <−50 kJ mol^−1^, except **II**-**1** with a single N1-H8…O1 being −56.37 kJ mol^−1^.

**Figure 2:**
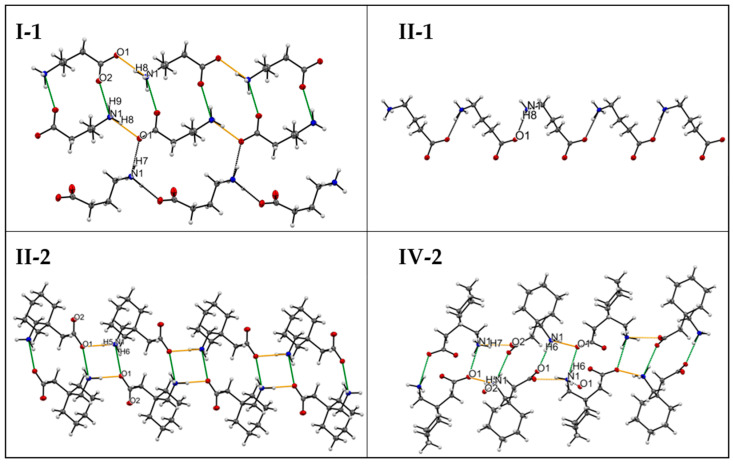
Strongest HB motives in GABA and gabapentin polymorphs: zig-zag chains of GABA molecules in **II**-**2**; layers in **I**-**1**, **II**-**1**, and **IV**-**2** built up of dimers (involved HBs are given in green) further linked to tetrameric motives (HBs in orange).

Even though the described HB characteristics of all investigated species do not vary much, there are some differences in their connectivity modes. The thermodynamic phases of GABA and gabapentin show similar overall crystal packing. A dimeric motif built up by N1-H9…O1 and N1-H6…O1 in **I**-**1** and **II**-**2**, respectively, connects two molecules via carboxylate and ammonium units (Figure 2). Such a dimeric motif is common for other GABA-related APIs, e.g., pregabalin [50,56], phenibut [49], or baclofen [52]. Dimers in **I**-**1** and **II**-**2** are further linked via two equal strongest HBs (N1-H8…O1 in **I**-**1** and N1-H5…O1 in **II**-**2**) to give tetrameric aggregates with the overall binding energy of −205.78 kJ mol^−1^ and -208.42 kJ mol^−1^, respectively. Tetramers propagate along the crystallographic axes to give a narrow 2D layer. Two N1-H6…O1 interactions build similar dimers in **IV**-**2**. Those, however, are connected via two different HBs, N1-H5…O1 and N1-H7…O2, resulting in an energetically slightly less beneficial tetramer (−202.62 kJ mol^−1^) compared to **II**-**2**. The latter one is still energetically robust, so once formed, **IV**-**2** remains stable over time. The similarity in the topology and energetic outcome is in line with the calculated ΔE_lat_ value and further explains the enantiotropic solid–solid phase transitions previously reported for gabapentin forms **II** and **IV**. These strongly bonded and energetically favorable carboxylate/ammonium tetramers are the driving force for the **I**-**1**, **II**-**2**, and **IV**-**2** phase formation. 

**Figure 3:**
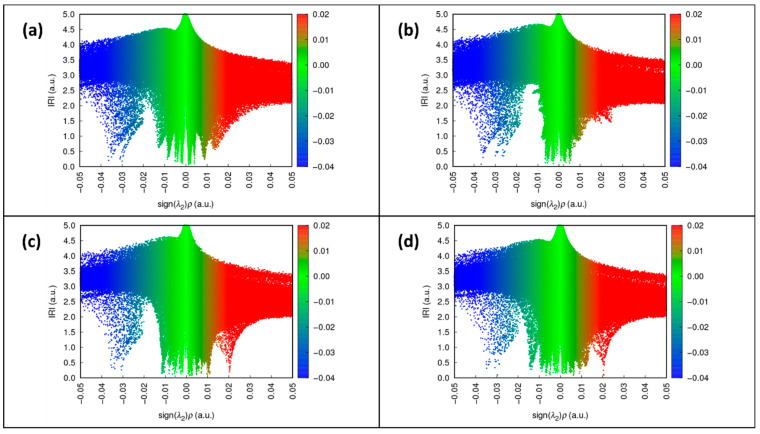
Interaction Region Indicator (IRI) scatterplots of intermolecular interaction in **1** and **2** polymorphs. Large spikes and broadness of the red region in **I**-**1** (**a**), **II**-**2** (**c**), and **IV**-**2** (**d**) indicate stronger repulsion compared to **II**-**1** (**b**). Colors for occurring interaction types: blue corresponds to HBs, green to van der Waals interactions, and red to intermolecular repulsion.

The described dimeric carboxylate/ammonium motif is noticeably missing in **II**-**1**, which could be the reason for its unfavorable formation under normal conditions despite the minute ΔE_lat_ value. Instead, GABA molecules form infinite zig-zag chains via single N1-H8…O1 interactions. Those are linked and stabilized by further strong attractive HB, which leads to a stable phase in accordance with the calculated ΔE_lat_. To understand why form **II**-**1** requires additives to promote its formation, a deeper inspection of further interaction types involved in the aggregation process might be helpful. NCI-based scatterplot analyses of the Interaction Region Indicator (IRI) type, as proposed by Lu et al. [16], provide additional information on the distribution of attractive and repulsive interactions in the solid state. The scatterplot analyses performed for **1** and **2** polymorphs (Figure 3) allow a direct visual comparison of the distribution for the occurring strong HBs, weak van der Waals interactions, and intermolecular repulsion. Whereas the distribution of strong HBs (blue regions) is similar for all polymorphs, the van der Waals interactions (green region) and molecular repulsion (red regions) are markedly less pronounced in **II**-**1** as some characteristic spikes in these regions are missing.

**Figure 4:**
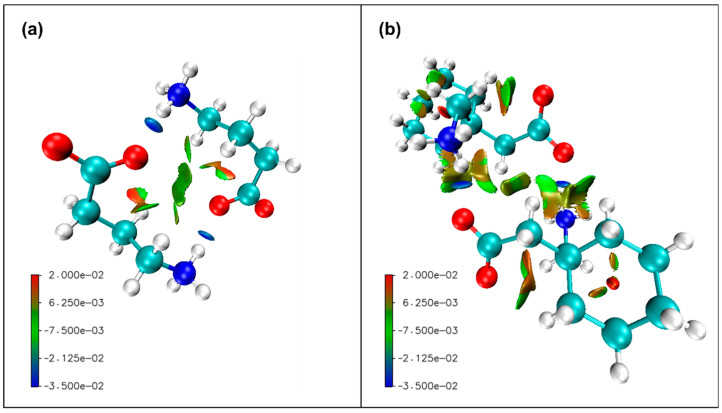
NCI visualization of a dimeric hydrogen bond interaction mode in **I**-**1** (**a**) and **II**-**2** (**b**). In both dimers, weakly attractive van der Waals interactions (green) and a repulsive (red) component occur next to the strongly attractive HBs (blue).

Visualization of the interaction motif under consideration of the distribution function could explain this observation. The otherwise very beneficial dimeric interaction brings not only subgroups with attractive potential but the whole GABA chains into very close contact with each other (Figure 4). The molecular orientation that enables the strongly binding HB motif at the same time causes a larger degree of intermolecular repulsion in **I**-**1**, **II**-**2**, and **IV**-**2**. On the contrary, a catemer arrangement in **I**-**1** does not force GABA molecules’ proximity; hence, the repulsion is minimized. This leads to the assumption that additives like acetic acid, on the one hand, hinder a tetramer formation to some degree; on the other hand, they induce less beneficial molecular conformation and act as a template for a cameter orientation, consequently allowing the formation of the less favored phase. Further, the minimization of the intermolecular repulsion for the observed conformation enables the formation of **II**-**1** under the accurately chosen conditions compared to **I**-**1** in the same environment.

### 3.2. Monohydrate of Gabapentin (***I-2***)

**Figure 5:**
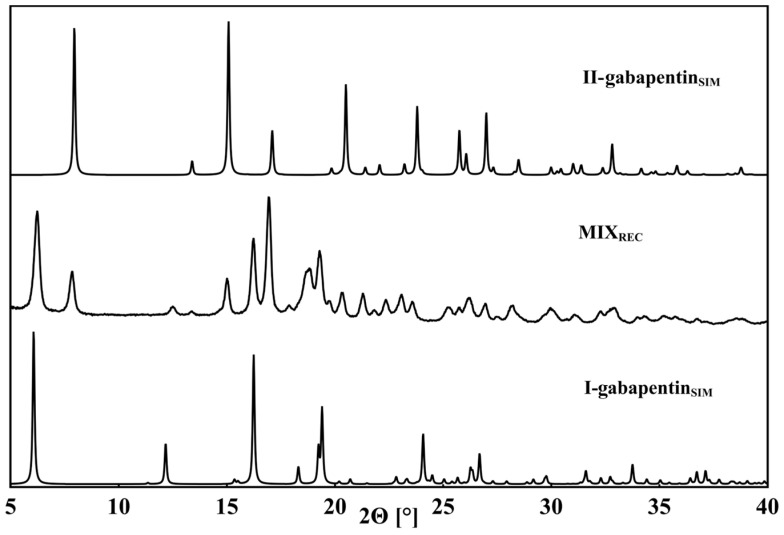
Powder pattern of a mixture of **I**- and **II**-gabapentin received after 30 min of neat grinding at 25 Hz compared to the simulated PXRDs of pure **II**-**2** and **I**-**2** phases.

The monohydrate of **2** shows a high propensity to form in the presence of water and is classified as a gabapentin **I** form [63]. While Lin et al. have reported that **I**-**2** can be converted to **II**-**2** under dry milling conditions for 2 h, we demonstrate that the opposite can also be the case. A neat mechanochemical grinding of **II**-**2** for 30 min at 25 Hz leads to the formation of a phase mixture of **II**-**2** and **I**-**2**, as confirmed by recorded PXRD (Figure 5). The milling vessel was not heat-dried prior to the experiment, and thus, adsorbed water from either the vessel walls or from the air must have enabled the hydrate formation. Similar behavior was observed for baclofen and phenibut in the past [48]. Solvent crystallization of **2** from aqueous solution without using the right additives also leads to the hydrate formation, which further implies its stability.

**Figure 6:**
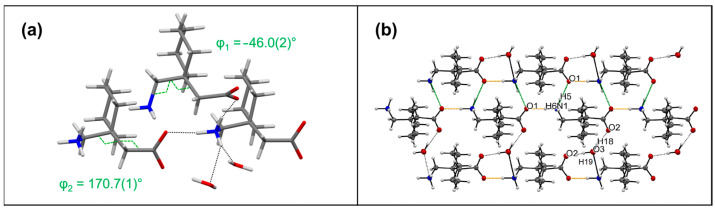
Some structural properties of **I**-**2**. (**a**) Selected hydrogen bonds for each symmetry inequivalent **2** molecule with corresponding torsions φ_1_ and φ_2_; (**b**) crystal packing of **I**-**2** with the strongest N1-H6…O1 HBs shown in orange, second-strongest interaction N1-H5…O1 shown in green, and HBs formed by water molecules shown in black dotted lines.

Investigation of the interaction motif shows the absence of any dimers that were deemed as a beneficial interaction motif in **I**-**1**, **II**-**2**, and **IV**-**2**, and the scatterplot indicates no noteworthy decrease in repulsion (Figure 6). Thus, taking into consideration the conducted structural observations on **1** and **2** polymorphs, the formation of **I**-**2** appears unlikely at first, converse to the experimental observations. At the same time, the distribution of intermolecular interactions on the NCI scatterplot points to the presence of stronger HBs than in the anhydrous polymorphs. Indeed, the quantification of the hydrogen bond strengths shows that the strongest occurring intermolecular interaction N1-H6…O1 with a binding energy of −66.71 kJ mol^−1^ in the hydrate surpasses any HB in anhydrous **1** or **2** forms by more than 10 kJ mol^−1^ (Table 1). It links gabapentin molecules to linear chains, which are further connected by two second-strongest N1-H5…O1 HBs of −51.46 kJ mol^−1^ each (Figure 6b) to give ribbons. Water molecules connect the ribbons over strong HBs, building a very stable hydrogen-bonded network with a higher number of attractive interactions compared to pure polymorphs.

As such, it seems likely that the multitude of strong HBs formed, including those involving water molecules and especially the strong N1-H6…O1 interaction, makes the formation of **I**-**2** energetically very beneficial and is the reason why it tends to easily form in the presence of water. The HB motif of **I**-**2** indicates that intermolecular attraction strength becomes the main force in dictating the structural makeup as no sufficiently strong competing influences take place.

### 3.3. Multi-Component Systems with Fumaric Acid (***3***)

**Figure 7:**
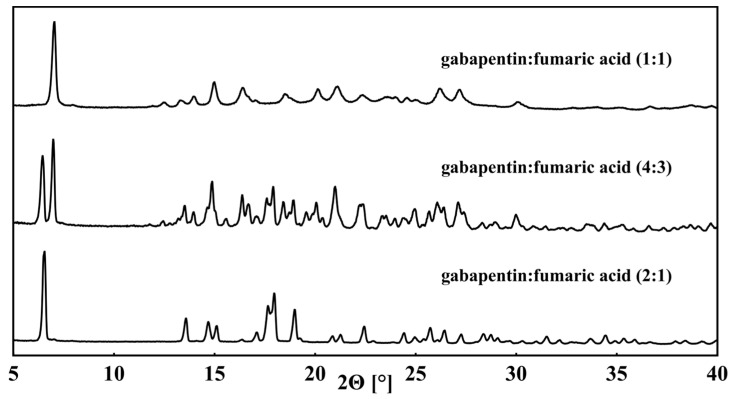
Selected PXRDs of neat grinding crystallizations of gabapentin and fumaric acid in different molar ratios: 1:1 (pure **2**-**3b**), 2:1 (pure **2**-**3a**), and 4:3 (phase mixture of **2**-**3a** and **2**-**3b**).

The introduction of a co-former with the potential to form strong hydrogen bonds appears to confirm the conducted observations regarding the crystal structural makeup for **1-** and **2**-based systems. GABA fumarate in a molar ratio of 2:1 (**1**-**3**), as well as two gabapentin fumarates in 2:1 (**2**-**3a**) and 1:1 (**2**-**3b**) ratios, were obtained from aqueous solution. In all presented fumarates, anhydrous salts are formed, where **3** is deprotonated once or twice, and carboxylate residues of **1** or **2** are protonated. The influence of the crystallization environment remains visible for gabapentin since the chosen amount of **3** can affect which salt of **2** is received. Depending on the amount of **3** introduced into the supramolecular crystallization, one or the other form is favored. Salt **2**-**3b** is formed once enough **3** is present, with **2**-**3a** only being produced when this amount is exceeded. A well-defined crystalline product can reproducibly be synthesized in a precursor ratio of 1:1 or 2:1, whereas, for example, a crystallization in 4:3 ratio results in a mixture of both phases (Figure 7). No solid–solid phase transition or degradation is observed under standard conditions over time for the reported **1** and **2** fumarates, which underlines their stability.

**Figure 8:**
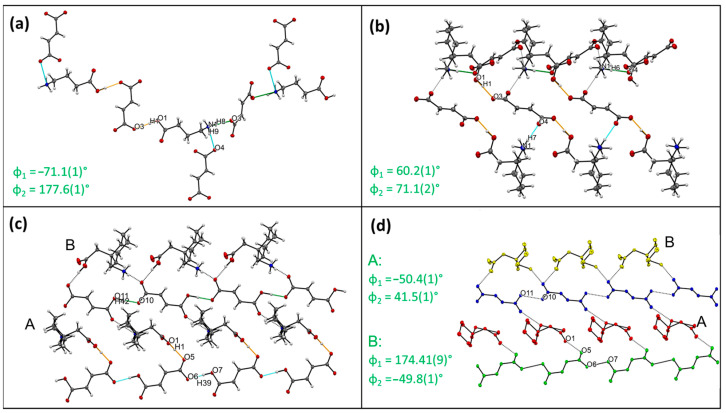
Torsion angles and basic structural motives in **1** and **2** fumarates based on the most attractive intermolecular interactions. Trimers in (**a**) **1**-**3** formed via O1-H1…O3 HBs and (**b**) **2**-**3a** via short O1-H1…O3; chains of fumarates in **2**-**3b** via O7-H39…O6 and O11-H42…O10 HBs (**c**) and the same motives with symmetry inequivalent **2** entities A and B shown in different colors (**d**). H-atoms are omitted in (**d**) for clarity. The strongest HBs are depicted in orange, the second-strongest in green, and the third-strongest in light blue.

Although the overall crystal architecture of **1**-**3** and **2**-**3b** differs, the interaction motifs are similar for both salt-like compounds in a 2:1 ratio. There are four distinctive HBs formed in each case, one via the **1** or **2** carboxyl to a **3** carboxylate and a further three for each ammonium hydrogen to a different **3** carboxylate oxygen. Additionally, **3** molecules become deprotonated from both sides, and strongly bonded adducts with either **1** or **2** molecules are formed. As shown in Figure 8, two strong carboxyl/carboxylate HB with an interaction energy of −65.80 kJ mol^−1^ (O1-H1…O3) in **1**-**3** and −73.09 kJ mol^−1^ (O1-H1…O3) in **2**-**3a** (Table 2) link one fumarate with two gabapentin moieties to give trimers of −259.06 kJ mol^−1^ and −135.60 kJ mol^−1^, respectively. In **1**-**3**, the second strongest HB (N1-H8…O3) is built between a GABA ammonium residue and a carboxylate group of the fumaric acid to give another trimeric motif. Both trimeric adducts propagate along the *b*-axis to 1D chains. In **2**-**3a**, the trimers are also connected via ammonium/carboxylate interactions, which, however, results in a zig-zag chain formation orthogonal to the trimeric motif. In both compounds, the ammonium residue further enables a three-dimensional connection to energetically stable networks.

Contrary to the previously discussed 2:1 fumarates, the **2**-**3b** crystallizes with four symmetry inequivalent molecules in an asymmetric unit cell, consisting of two independent **2** and **3** entities each. Consequently, the number of HBs is remarkably higher, and the overall crystal packing is more complex than in **1**-**3** and **2**-**3a**. The 13 distinctive HBs allow higher variation in bond strengths, lengths, and angles than in any of the aforementioned systems. Especially, those HBs formed between carboxyl O-H and carboxylate oxygen stand out, with bond strengths between −65.75 kJ mol^−1^ and −111.08 kJ mol^−1^ (Table S24 in ESI). Further, **2**-**3b** features the two strongest hydrogen bonds of all reported systems. Hereby, the second- and third-strongest HBs (−110.63 kJ mol^−1^ and −76.81 kJ mol^−1^) link **3** entities of the same symmetry to form infinite chains, and the strongest O1-H1…O5 HB occurs between **2** carboxyl group and **3** carboxylate as a single interaction. Interestingly, two crystallographically independent **2** molecules do not interact with each other, and neither do the two independent **3** molecules. The connection between these two symmetrically unrelated motifs is realized via numerous N-H…O and O-H…O HBs between alternating **2** and **3** entities. 

IRI scatterplots further confirm the strength of hydrogen bonds for all three fumarates. Spikes occurring in the blue regions of highly attractive HBs visually highlight how strong the described carboxyl/carboxylate bonds are, particularly in **2**-**3b** (for more details, see ESI). Concerning the torsion angles, no clear relation to the conformational impact, as discussed for **1** and **2**, can be observed. This points out how the changed crystallization conditions and energetic benefits from the intermolecular interactions overshadow molecular conformations that are favorable for single-component phases.

### 3.4. Multi-Component Systems with Succinic Acid (***4***)

**Figure 9:**
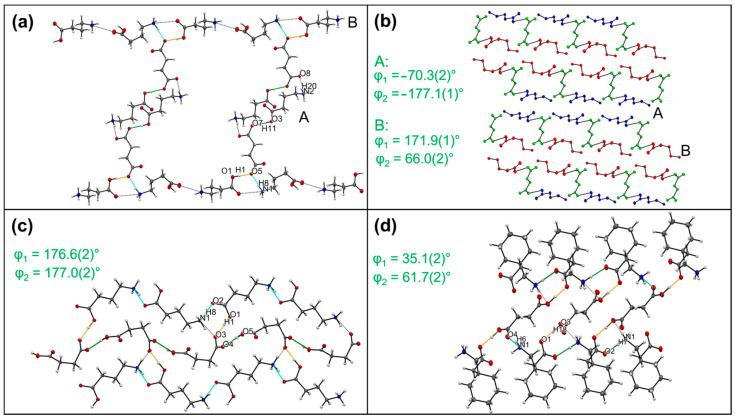
Torsion angles and basic structural motives in **1** succinates and **2** succinic acid co-crystal based on the most attractive intermolecular interactions: (**a**) inverting trimers in **1**-**4a**; (**b**) overall packing of trimers in **1**-**4a** along *a*-axis with symmetry inequivalent entities highlighted in different colors (GABA in blue and red also marked as A and B, succinic acid in green); (**c**) alternating chains of **1** and **4** in **1**-**4b**; (**d**) trimers in **2**-**4** further connected via ammonium/carboxylate HB between **2** molecules. H-atoms are omitted in (**b**) for clarity. The strongest HBs are depicted in orange, the second-strongest in green, and third-strongest in light blue.

Multi-component crystalline systems formed with **4** show some similarities to the described fumarates. Three compounds were reproducibly synthesized and showed comparable phase stability to the fumarates over time. Hydrogen bonds remain the driving force in **4**-based as well as in **3**-based systems, all crystallized in monoclinic space groups (Table 2, ESI). However, contrary to **3**-based salts, two products of GABA in different ratios are obtained, the 2:1 phase (**1**-**4a**) and its 1:1 modification **1**-**4b**; **4** is deprotonated twice in both compounds. The 2:1 ratio of **1**-**4a** is reflected in three crystallographically distinct molecules, while **1**-**4b** consists of one independent **1** and **4** molecule each. Accordingly, in **1**-**4a**, one fumarate entity links two GABA molecules over strong carboxyl/carboxylate O1-H1…O5 and O3-H11…O7 HBs to trimers with the binding energy of −149.54 kJ mol^−1^ (Figure 9) comparable to **2**-**3a**. The same **4** carboxylate undergoes a further interaction with the ammonium group of another **1** (N1-H8…O5). Trimers are inverted with respect to each other, with two GABA molecules of the same symmetry being stabilized by N-H…O HBs. The **1**-**4b** aggregates differently. The second- and third-strongest interactions (O5-H11…O4 and N1-H8…O2) form alternating chains of whether **1** or **4** molecules. O1-H1…O3 HB, the strongest interaction in this structure with −104.67 kJ mol^−1^, connect the chains into 2D layers, resulting in a similar arrangement discussed for **2**-**3b**.

In the case of **2**-**4**, a co-crystal instead of a salt is received. Even though this is less common for the discussed compounds and similar entities, exceptions exist, for example, in homochiral pregabalin and mandelic acid co-crystals [50,54]. As with all other 2:1 systems, **1**-**4** is also driven by the formation of an energetically strong trimeric motif where one succinic acid links two gabapentin molecules via two equal O3-H18…O1 HBs. The binding energy of this trimer accounts for −223.00 kJ mol^−1^, being in the same range as **1**-**3**. Trimers are directly connected over ammonium/carboxylate HB to form chains of gabapentin molecules.

NCI scatterplots and the dihedral angles of GABA moiety coincide with those for fumarates, emphasizing the strength of HBs on the one hand and, then again, showing no clear conformational influence on the phase formation compared to that in polymorphs of **1** or **2**. Further, all **3**- and **4**-based compounds have in common at least one highly attractive hydrogen bond and a secondary network of additional connections that form around the directional carboxyl/carboxylate interaction. In addition, the calculated binding energies of these interactions are in the same range for both fumarates and succinates. The weakest of them occurs in **1**-**4b** (O3-H11…O7) with −64.86 kJ mol^−1^ and the most attractive in **2**-**4** with −111.50 kJ mol^−1^ (O3-H18…O1). The latter shows that HB of similar strength can occur even in co-crystals due to the ionic character of subgroups in zwitterionic systems.

Strong HBs remain the most important force of attraction in the crystal structures of all the discussed compounds. Calculations of their strengths indicate how strong HBs favor the formation of the salts and co-crystals over the single-component polymorphs. Although being strong and dominant in **1** and **2** polymorphs, ammonium/carboxylate N-H…O HBs are energetically significantly inferior to carboxyl/carboxylate O-H…O, a phenomenon observed in all analyzed fumarates and succinates. Gabapentin hydrate exhibits both kinds of interactions. Together with a high overall number of strong HBs, the formation of **I**-**2** is energetically beneficial compared to the anhydrous forms. Still, carboxyl/carboxylate interactions clearly dominate the aggregation process, which further explains a favored formation of multi-component systems over **I**-**2** or **1** and **2** polymorphs from the aqueous solution as well as their stability. Conformation of the GABA moiety plays an important role in polymorphic crystallization and has no noticeable impact in the case of multi-component systems, including hydrate, since it cannot compete with strong directional HBs.

## 4. Conclusions

Polymorphs of GABA and gabapentin, as well as gabapentin hydrate, are structurally revisited, five salts and one co-crystal are synthesized and investigated. A delicate balance of influences of the crystallization environment favors the formation of one form or the other polymorphic form. Small calculated lattice energy differences between the polymorphs presume their similar stability. This is confirmed by quantification of the HB interaction strengths and motives. Although the calculated energy values for the occurring HBs are comparable in all polymorphs, the formation of strongly bonded ammonium/carboxylate dimeric and further tetrameric HB motifs of around −95 kJ mol^−1^ and −205 kJ mol^−1^, respectively, promotes easy crystallization of **I**-**1**, **II**-**2**, and forms. However, if the crystallization environment favors the growth of seeds not incorporating said motif, the result will disregard the slight gain in energy. Other past contributions have shown that there appears to be a connection with the molecular conformation of the GABA chain, changing under such environmental influences. We show that it is not only a beneficial crystallization environment, like the presence of additives but its combination with the minimization of the intermolecular repulsion for the observed molecular orientation that makes the otherwise elusive polymorph **II**-**1** accessible. The formation of **I**-gabapentin indicates how easily this polymorphic equilibrium can be disturbed. The introduction of water molecules into the crystal lattice raises the number, strength, and available types of HBs, which results in a phase transition of gabapentin polymorphs to its hydrate even in the presence of trace amounts of water. This becomes even more prevalent if dicarboxylic acids capable of being strong hydrogen bond donors or acceptors are introduced. Carboxyl/carboxylate HBs occurring in all salts and a co-crystal (the highest in a gabapentin:succinic acid co-crystal **2**-**4** with −111.50 kJ mol^−1^) are remarkably stronger than ammonium/carboxylate N-H…O interactions in single component systems and lead to energetically beneficial trimeric or chain-like aggregates. It appears likely that during crystallization, these highly attractive interactions or motives occur first, and everything else subordinates accordingly. Any conformational impact is hereby overshadowed, visible in the high variations of the torsion angles. To conclude, although the crystallization environment still remains the superordinate force, strong intermolecular interactions commence and predefine the preferred molecular orientation in solution and, at the end, in the solid phase. The control over a desired phase formation and further insight into its driving forces can provide input for the understanding of the specific recognition modes of GABA and gabapentin in biological processes. Accurate choice of the crystallization conditions, ratios, and interactions involved allows to reliably obtain a targeted single- same as multi-component phase. Multi-component species like salts or co-crystals can further minimize phase transition potential by introducing exceedingly beneficial intermolecular interactions that make such transitions unlikely. Understanding the aggregation phenomena of salts or co-crystals has the potential for the development of novel GABA-based multi-component drugs.

## Figures and Tables

**Table 1:** Overview of the strongest hydrogen bonds and their main characteristics in the investigated GABA polymorphs, gabapentin polymorphs, and gabapentin monohydrate (**I**-**2**).

Compound	Interaction	H…A [Å]	D…A [Å]	D-H…A [°]	E_bond_ [kJ mol^−1^]
	N1-H8…O1	1.81 (1)	2.760 (3)	168 (5)	−55.37
**I**-**1**	N1-H7…O1	1.82 (0)	2.734 (2)	169 (3)	−54.83
	N1-H9…O2	1.85 (3)	2.755 (7)	163 (1)	−47.52
	N1-H8…O1	1.82 (2)	2.753 (3)	164 (8)	−56.37
**II**-**1**	N1-H9…O2	1.90 (2)	2.743 (9)	179 (4)	−46.88
	N1-H7…O1	1.94 (3)	2.790 (1)	172 (2)	−42.87
	N1-H5…O1	1.80 (2)	2.753 (5)	168 (0)	−55.48
**II**-**2**	N1-H7…O2	1.83 (8)	2.746 (7)	168 (2)	−52.97
	N1-H6…O1	1.88 (2)	2.778 (3)	158 (6)	−48.73
	N1-H7…O2	1.81 (6)	2.733 (8)	164 (4)	−54.65
**IV**-**2**	N1-H5…O1	1.83 (0)	2.769 (2)	168 (2)	−53.87
	N1-H6…O1	1.87 (9)	2.796 (2)	164 (1)	−47.05
	N1-H6…O1	1.73 (2)	2.752 (2)	173 (2)	−66.71
**I**-**2**	N1-H5…O1	1.88 (3)	2.842 (2)	166 (2)	−51.46
	O3-H19…O2	1.82 (3)	2.752 (2)	176 (2)	−49.12

**Table 2:** Overview of the three strongest hydrogen bonds in the fumarates of GABA and gabapentin, succinates of GABA, and the gabapentin:succinic acid co-crystal. The shortest contacts and lowest energy values in each compound are highlighted in bold.

Compound	Interaction	H…A [Å]	D…A [Å]	D-H…A [°]	E_bond_ [kJ mol^−1^]
	O1-H1…O3	**1.72 (2)**	2.606 (7)	179 (2)	**−65.80**
**1**-**3**	N1-H8…O3	1.89 (9)	2.785 (9)	163 (6)	−48.84
	N1-H9…O4	1.91 (0)	2.785 (1)	164 (3)	−49.63
	O1-H1…O3	**1.68 (3)**	2.644 (3)	174 (2)	**−73.09**
**2**-**3a**	N1-H6…O4	1.81 (2)	2.761 (0)	174 (6)	−58.11
	N1-H7…O4	1.84 (2)	2.746 (4)	162 (2)	−53.54
	O11-H42…O10	**1.50 (2)**	2.525 (5)	178 (3)	**−110.63**
**2**-**3b**	O1-H1…O5	**1.51 (2)**	2.507 (0)	175 (2)	**−111.08**
	O7-H39…O6	1.65 (2)	2.559 (0)	170 (5)	−76.81
	O1-H1…O5	1.62 (3)	2.543 (6)	173 (2)	**−84.68**
**1**-**4a**	O3-H11…O7	1.72 (3)	2.581 (3)	178 (2)	−64.86
	N1-H8…O5	1.84 (2)	2.783 (8)	173 (2)	−52.26
	O1-H1…O3	**1.52 (3)**	2.455 (2)	175 (4)	**−104.67**
**1**-**4b**	O5-H11…O4	1.78 (4)	2.583 (2)	163 (3)	−52.74
	N1-H8…O2	1.95 (3)	2.861 (3)	161 (3)	−38.57
	O3-H18…O1	**1.50 (3)**	2.522 (0)	175 (2)	**−111.50**
**2**-**4**	N1-H7…O2	1.79 (3)	2.734 (3)	175 (2)	−56.03
	N1-H6…O4	1.94 (2)	2.776 (0)	155 (2)	−38.62

## Data Availability

Not applicable.

## Supplementary Materials

The following supporting information can be downloaded at https://www.mdpi.com/article/10.3390/pharmaceutics15092299/s1, Table S1: Crystallographic data for **I**-GABA; Table S2: Distinctive energy values for the occurring HB obtained by AIM analysis via multiwfn conducted as assumed charged HB for two molecules (E_1_), and complete interaction sphere of distinctive HB around one molecule (E_2_), and as assumed neutral HB under the same conditions for E_1_*, E_2_*; Figure S1: Powder pattern comparison of **I**-GABA. Simulated from single crystal data (top), recorded substance (bottom). A range between 5° 2Θ and 40° 2Θ is depicted; Figure S2: IR spectrum of **I**-GABA, shown in a range between 4000 cm^−1^ and 400 cm^−1^. Broad ammonium hydrogen bond network and C-H stretch band between 3200 cm^−1^ and 2270 cm^−1^, carboxylate stretch band at 1610 cm^−1^; Figure S3: Interaction Region Indicator surfaces and related scatter plots of the distinctive hydrogen bonds in **I**-GABA: N1-H7…O1 (a), N1-H8…O1 (b), and N1-H9…O2 (c). Blue regions signify strong attraction, green regions weak attraction, and red regions repulsion; Table S3: Crystallographic data for **II**-GABA; Table S4: Distinctive energy values for the occurring HB obtained by AIM analysis via multiwfn conducted as assumed charged HB for two molecules (E_1_), and complete interaction sphere of distinctive HB around one molecule (E_2_), and as assumed neutral HB under the same conditions for E_1_*, E_2_*; Figure S4: Powder pattern comparison of **II**-GABA. Simulated from single crystal data (top), recorded substance (bottom). A range between 5° 2Θ and 40° 2Θ is depicted; Figure S5: IR spectrum of **II**-GABA, shown in a range between 4000 cm^−1^ and 400 cm^−1^. Broad ammonium hydrogen bond network and C-H stretch band between 3700 cm^−1^ and 2270 cm^−1^, carboxylate stretch band at 1649 cm^−1^; Figure S6: Interaction Region Indicator surfaces and related scatter plots of the distinctive hydrogen bonds in **II**-GABA: N1-H7…O1 (a), N1-H8…O1 (b), and N1-H9…O2 (c). Blue regions signify strong attraction, green regions weak attraction, and red regions repulsion; Table S5: Crystallographic data for **II**-gabapentin; Table S6: Distinctive energy values for the occurring HB obtained by AIM analysis via multiwfn conducted as assumed charged HB for two molecules (E_1_), and complete interaction sphere of distinctive HB around one molecule (E_2_), and as assumed neutral HB under the same conditions for E_1_*, E_2_*; Figure S7: Powder pattern comparison of **II**-gabapentin. Simulated from single crystal data (top), recorded substance (bottom). A range between 5° 2Θ and 40° 2Θ is depicted; Figure S8: IR spectrum of **II**-gabapentin, shown in a range between 4000 cm^−1^ and 400 cm^−1^. Broad ammonium hydrogen bond network and C-H stretch band between 3200 cm^−1^ and 2240 cm^−1^, carboxylate band at 1611 cm^−1^; Figure S9: Interaction Region Indicator surfaces and related scatter plots of the distinctive hydrogen bonds in **II**-gabapentin: HB N1-H5…O1 (a), N1-H6…O1 (b), and N1-H7…O2 (c). Blue regions signify strong attraction, green regions weak attraction, and red regions repulsion; Table S7: Crystallographic data for **IV**-gabapentin; Table S8: Distinctive energy values for the occurring HB obtained by AIM analysis via multiwfn conducted as assumed charged HB for two molecules (E_1_), and complete interaction sphere of distinctive HB around one molecule (E_2_), and as assumed neutral HB under the same conditions for E_1_*, E_2_*; Figure S10: Powder pattern comparison of **IV**-gabapentin. Simulated from single crystal data (top), recorded substance (bottom). A range between 5° 2Θ and 40° 2Θ is depicted; Figure S11: IR spectrum of **IV**-gabapentin, shown in a range between 4000 cm^−1^ and 400 cm^−1^. Broad ammonium hydrogen bond network and C-H stretch band between 3500 cm^−1^ and 2270 cm^−1^, carboxylate band at 1641 cm^−1^; Figure S12: Interaction Region Indicator surfaces and related scatter plots of the distinctive hydrogen bonds in **IV**-gabapentin: N1-H5…O1 (a), N1-H6…O1 (b), and N1-H7…O2 (c). Blue regions signify strong attraction, green regions weak attraction, and red regions repulsion; Table S9: Crystallographic data for gabapentin • H_2_O (**I**-gabapentin); Table S10: Distinctive energy values for the occurring HB obtained by AIM analysis via multiwfn conducted as assumed charged HB for two molecules (E_1_), and complete interaction sphere of distinctive HB around one molecule (E_2_), and as assumed neutral HB under the same conditions for E_1_*, E_2_*; Table S11: Overview of the strong, distinctive hydrogen bonds in gabapentin monohydrate. The proton acceptor distance, the donor acceptor distance, the bond angle, and the binding energy calculated for charged HBs are shown.; Figure S13: Powder pattern comparison of **I**-gabapentin. Simulated from single crystal data (top), recorded substance (bottom). A range between 5° 2Θ and 40° 2Θ is depicted; Figure S14: IR spectrum of **I**-gabapentin, shown in a range between 4000 cm^−1^ and 400 cm^−1^. Broad ammonium hydrogen bond network and C-H stretch band between 3625 cm^−1^ and 2240 cm^−1^, water band at 3270 cm^−1,^ and carboxylate band at 1659 cm^−1^; Figure S15: Interaction Region Indicator surfaces and related scatter plots of three distinctive hydrogen bonds in **I**-gabapentin: HB N1-H5…O1 (a), N1-H6…O1 (b), and N1-H7…O3 (c). Blue regions signify strong attraction, green regions weak attraction, and red regions repulsion; Figure S16: Interaction Region Indicator surfaces and related scatter plots of three distinctive hydrogen bonds in **I**-gabapentin: HB N1-H7…O3 (a), O3-H18…O2 (b), and O3-H19…O2 (c). Blue regions signify strong attraction, green regions weak attraction, and red regions repulsion; Table S12: Crystallographic data for GABA fumarate (2:1); Table S13: Distinctive energy values for the occurring HB obtained by AIM analysis via multiwfn conducted as assumed charged HB for two molecules (E_1_), and complete interaction sphere of distinctive HB around one molecule (E_2_), and as assumed neutral HB under the same conditions for E_1_*, E_2_*; Figure S17. Powder pattern comparison of GABA fumarate (2:1). Simulated from single crystal data (top), recorded substance (bottom). A range between 5° 2Θ and 40° 2Θ is depicted; Figure S18: IR spectrum of GABA fumarate (2:1), shown in a range between 4000 cm^−1^ and 400 cm^−1^. Broad ammonium hydrogen bond network and C-H stretch band between 3640 cm^−1^ and 2240 cm^−1^, carboxyl band at 1724 cm^−1,^ carboxylate band at 1626 cm^−1^; Figure S19: Interaction Region Indicator surfaces and related scatter plots of two distinctive hydrogen bonds in GABA fumarate (2:1): N1-H8…O3 (a), and N1-H9…O4 (b). Blue regions signify strong attraction, green regions weak attraction, and red regions repulsion; Figure S20: Interaction Region Indicator surfaces and related scatter plots of two distinctive hydrogen bonds in GABA fumarate (2:1): N1-H10…O4 (a), and O1-H1…O3 (b). Blue regions signify strong attraction, green regions weak attraction, and red regions repulsion; Table S14: Crystallographic data for gabapentin fumarate (2:1); Table S15: Distinctive energy values for the occurring HB obtained by AIM analysis via multiwfn conducted as assumed charged HB for two molecules (E_1_), and complete interaction sphere of distinctive HB around one molecule (E_2_), and as assumed neutral HB under the same conditions for E_1_*, E_2_*; Figure S21: Powder pattern comparison of gabapentin fumarate (2:1). Simulated from single crystal data (top), recorded substance (bottom). A range between 5° 2Θ and 40° 2Θ is depicted; Figure S22: IR spectrum of gabapentin fumarate (2:1), shown in a range between 4000 cm^−1^ and 400 cm^−1^. Broad ammonium hydrogen bond network and C-H stretch band between 3435 cm^−1^ and 2170 cm^−1^, carboxyl band at 1708 cm^−1,^ carboxylate band at 1627 cm^−1^; Figure S23: Interaction Region Indicator surfaces and related scatter plots of two distinctive hydrogen bonds in gabapentin fumarate (2:1): N1-H6…O4 (a), and N1-H7…O4 (b). Blue regions signify strong attraction, green regions weak attraction, and red regions repulsion; Figure S24: Interaction Region Indicator surfaces and related scatter plots of two distinctive hydrogen bonds in gabapentin fumarate (2:1): N1-H8…O3 (a), and O1-H1…O3 (b). Blue regions signify strong attraction, green regions weak attraction, and red regions repulsion; Table S16: Crystallographic data for gabapentin fumarate (1:1); Table S17: Distinctive energy values for the occurring HB obtained by AIM analysis via multiwfn conducted as assumed charged HB for two molecules (E_1_), and complete interaction sphere of distinctive HB around one molecule (E_2_), and as assumed neutral HB under the same conditions for E_1_*, E_2_*; Figure S25: Powder pattern comparison of gabapentin fumarate (1:1). Simulated from single crystal data (top), recorded substance (bottom). A range between 5° 2Θ and 40° 2Θ is depicted; Figure S26. IR spectrum of gabapentin fumarate (1:1), shown in a range between 4000 cm^−1^ and 400 cm^−1^. Broad ammonium hydrogen bond network and C-H stretch band between 3400 cm^−1^ and 2195 cm^−1^, carboxyl band at 1704 cm^−1,^ carboxylate band at 1636 cm^−1^; Figure S27: Interaction Region Indicator surfaces and related scatter plots of three distinctive hydrogen bonds in gabapentin fumarate (1:1): N1-H7…O10 (a), N1-H7…O12 (b), and N1-H8…O2 (c). The intramolecular hydrogen bond N1-H6…O2 is best visible in (c) but present in each depiction. Blue regions signify strong attraction, green regions weak attraction, and red regions repulsion; Figure S28: Interaction Region Indicator surfaces and related scatter plots of three distinctive hydrogen bonds in gabapentin fumarate (1:1): N1-H8…O12 (a), N2-H24…O8 (b), and N2-H25…O9 (c). The intramolecular hydrogen bond N1-H6…O2 is best visible in a). Blue regions signify strong attraction, green regions weak attraction, and red regions repulsion; Figure S29: Interaction Region Indicator surfaces and related scatter plots of two distinctive hydrogen bonds in gabapentin fumarate (1:1): O7-H39…O6 (a), and O11-H42…O10 (b). The hydrogen bond strength in b) crossed the threshold for weak covalent interactions. Blue regions signify strong attraction, green regions weak attraction, and red regions repulsion; Table S18: Crystallographic data for GABA succinate (2:1); Table S19: Distinctive energy values for the occurring HB obtained by AIM analysis via multiwfn conducted as assumed charged HB for two molecules (E_1_), and complete interaction sphere of distinctive HB around one molecule (E_2_), and as assumed neutral HB under the same conditions for E_1_*, E_2_*; Figure S30: Powder pattern comparison of GABA succinate (2:1). Simulated from single crystal data (top), recorded substance (bottom). A range between 5° 2Θ and 40° 2Θ is depicted; Figure S31: IR spectrum of GABA succinate (2:1), shown in a range between 4000 cm^−1^ and 400 cm^−1^. Broad ammonium hydrogen bond network and C-H stretch band between 3700 cm^−1^ and 2240 cm^−1^, carboxyl band at 1718 cm^−1,^ carboxylate band at 1620 cm^−1^; Figure S32: Interaction Region Indicator surfaces and related scatter plots of three distinctive hydrogen bonds in GABA succinate (2:1): N1-H8…O5 (a), N1-H9…O6 (b), and N1-H10…O6 (c). Blue regions signify strong attraction, green regions weak attraction, and red regions repulsion; Figure S33: Interaction Region Indicator surfaces and related scatter plots of three distinctive hydrogen bonds in GABA succinate (2:1): N2-H18…O7 (a), N2-H19…O8 (b), and N2-H20…O8 (c). Blue regions signify strong attraction, green regions weak attraction, and red regions repulsion; Figure S34: Interaction Region Indicator surfaces and related scatter plots of two distinctive hydrogen bonds in GABA succinate (2:1): O1-H1…O5 (a), and O3-H11…O7 (b). Blue regions signify strong attraction, green regions weak attraction, and red regions repulsion; Table S20: Crystallographic data GABA succinate (1:1); Table S21: Distinctive energy values for the occurring HB obtained by AIM analysis via multiwfn conducted as assumed charged HB for two molecules (E_1_), and complete interaction sphere of distinctive HB around one molecule (E_2_), and as assumed neutral HB under the same conditions for E_1_*, E_2_*; Figure S35: Powder pattern comparison of GABA succinate (1:1). Simulated from single crystal data (top), recorded substance (bottom). A range between 5° 2Θ and 40° 2Θ is depicted; Figure S36: IR spectrum of GABA succinate (1:1), shown in a range between 4000 cm^−1^ and 400 cm^−1^. Broad ammonium hydrogen bond network and C-H stretch band between 3690 cm^−1^ and 2210 cm^−1^, carboxyl band at 1680 cm^−1,^ carboxylate band at 1618 cm^−1^; Figure S37: Interaction Region Indicator surfaces and related scatter plots of three distinctive hydrogen bonds in GABA succinate (1:1): N1-H8…O2 (a), N1-H9…O2 (b), and N1-H10…O6 (c). Blue regions signify strong attraction, green regions weak attraction, and red regions repulsion; Figure S38: Interaction Region Indicator surfaces and related scatter plots of two distinctive hydrogen bonds in GABA succinate (1:1): O1-H1…O3 (a), and O5-H11…O4 (b). Blue regions signify strong attraction, green regions weak attraction, and red regions repulsion; Figure S39: Interaction Region Indicator surfaces and related scatter plots of two distinctive hydrogen bonds in GABA succinate (1:1): O1 H1…O3 (a), and O5-H11…O4 (b). Blue regions signify strong attraction, green regions weak attraction, and red regions repulsion; Table S22: Crystallographic data for gabapentin:succinic acid (2:1); Table S23: Distinctive energy values for the occurring HB obtained by AIM analysis via multiwfn conducted as assumed charged HB for two molecules (E_1_), and complete interaction sphere of distinctive HB around one molecule (E_2_), and as assumed neutral HB under the same conditions for E_1_*, E_2_*; Figure S40: Powder pattern comparison of gabapentin:succinic acid (2:1). Simulated from single crystal data (top), recorded substance (bottom). A range between 5° 2Θ and 40° 2Θ is depicted; Figure S41: IR spectrum of gabapentin:succinic acid (2:1), shown in a range between 4000 cm^−1^ and 400 cm^−1^. Broad ammonium hydrogen bond network and C-H stretch band between 3660 cm^−1^ and 2225 cm^−1^, carboxyl band at 1704 cm^−1,^ carboxylate band at 1676 cm^−1^; Figure S42: Interaction Region Indicator surfaces and related scatter plots of two distinctive hydrogen bonds in gabapentin:succinic acid (2:1): N1-H5…O3 (a), and N1-H6…O4 (b). The intramolecular hydrogen bond N1-H5…O1 is best visible in a) but present in both. Blue regions signify strong attraction, green regions weak attraction, and red regions repulsion; Figure S43: Interaction Region Indicator surfaces and related scatter plots of two distinctive hydrogen bonds in gabapentin:succinic acid (2:1): N1-H7…O2 (a), and O3-H18…O1 (b). The intramolecular hydrogen bond N1-H5…O1 is best visible in (a), but present in both. The hydrogen bond strength in (b) crossed the threshold for weak covalent interactions. Blue regions signify strong attraction, green regions weak attraction, and red regions repulsion; Figure S44: Torsion angles and selected hydrogen bonds for (a) **1**-**3**, (b) **2**-**3a**, (c) first independent gabapentin molecule in **2**-**3b**, and (d) second independent gabapentin molecule in **2**-**3b**. HBs are depicted as black dotted lines, torsion angles are only depicted as values for better visibility, oxygen atoms in red, nitrogen atoms in blue, carbon atoms in grey, and hydrogen atoms in white; Table S24: Overview of the strong, distinctive hydrogen bonds in the fumarates of GABA and gabapentin. The proton acceptor distance, the donor acceptor distance, the bond angle, and the binding energy calculated for charged HBs are shown; Figure S45: Scatterplots of HB-interaction spheres of **1**-**3** (a), **2**-**3a** (b), the interaction sphere around the first distinctive **2** molecule in **2**-**3b** (c), and the same for the second distinctive **2** molecule in **2**-**3b** (d). Colors indicate occurring interaction types: blue corresponds to HBs, green to van der Waals interactions, and red to intermolecular repulsion; Figure S46: Torsion angles and selected hydrogen bonds for (a) **2**-**4**, (b) **1**-**4b**, (c) first distinctive GABA molecule in **1**-**4b**, and (d) second distinctive GABA molecule in **1**-**4a**. Hydrogen bonds are depicted as black dotted lines, torsion angles are only depicted as values for better visibility, oxygen atoms in red, nitrogen atoms in blue, carbon atoms in grey, and hydrogen atoms in white; Table S25: Overview of the strong, distinctive hydrogen bonds in the succinates of GABA and the gabapentin:succinic acid co-crystal. The proton acceptor distance, the donor acceptor distance, the bond angle, and the binding energy calculated for charged HBs are shown; Figure S47: Scatterplots of HB-interaction spheres of **2**-**4** (a), **1**-**4a** (b), the interaction sphere around the first distinctive **1** molecule in **1**-**4b** (c), and the same for the second distinctive **1** molecule in **1**-**4b** (d). Colors indicate occurring interaction types: blue corresponds to HBs, green to van der Waals interactions, and red to intermolecular repulsion.
