# Supplementary material for: Polymorphism and Multi-Component Crystal Formation of GABA and Gabapentin
Source: Pharmaceutics. 2023 Sep 10;15(9):2299. doi: 10.3390/pharmaceutics15092299 (PMC10536459; doi:10.3390/pharmaceutics15092299)
Supplement: Supplementary file 1 [file pharmaceutics-15-02299-s001.zip › pharmaceutics-2519018-supplementary.pdf]

Supporting Information

Polymorphism and Multicomponent Crystal Formation of GABA and Gabapentin

*D. Komisarek, F. Demirbas, T. Haj Hassani Sohi, K. Merz, C. Schauerte and V. Vasylyeva**

Contents

I-GABA (**I-1**) 1

II-GABA (**II-1**) 4

II-Gabapentin (**II-2**) 7

IV-Gabapentin (**IV-2**) 10

Gabapentin • H_2O (I-Gabapentin) (**I-2**) 13

GABA Fumarate (2:1) (**1-3**) 18

Gabapentin Fumarate (2:1) (**2-3a**) 22

Gabapentin Fumarate (1:1) (**2-3b**) 26

GABA Succinate (2:1) (**1-4a**) 32

GABA Succinate (1:1) (**1-4b**) 37

Gabapentin:Succinic acid (2:1) (**2-4**) 41

Comparison of **1** and **2** multicomponent systems 45

I-GABA (I-1)

Table S1. *Crystallographic data for **I**-GABA.*

**Parameters**	**I-GABA**
**Formula**	$C_4 H_9 N O_2$
**Formula Moiety**	$C_4 H_9 N O_2$
**M_r [g mol^{-1}]**	103.12
**Temperature [K]**	100.00(1)
**System/space group**	monoclinic, *$P2_1/c$*
**a (Å)**	7.2196(8)
**b (Å)**	9.9985(7)
**c (Å)**	8.2154(8)
**α (°)**	90
**β (°)**	110.609(10)
**γ (°)**	90
**V (Å^3)**	555.08(10)
**Z/Z'**	4/1
**Density [g/cm^3]**	1.234
**μ [mm^{-1}]**	0.826
**T_{min}/T_{max}**	0.749/1.000
**F (000)**	224
**Crystal size [mm]**	0.05 · 0.11 · 0.17
**2θ range [°]**	6.6 – 77.2
**Completeness [%]**	99.6
**Recorded refl.**	3100
**Independent refl.**	1064
**Goodness-of-fit F^2**	1.112
**X-Ray Source**	Cu Kα (λ = 1.54184)
**R_1 [%] /wR_2 [%] /S**	3.47/ 8.70/ 1.11

Table S2. *Distinctive energy values for the occurring HB obtained by AIM analysis via multiwfn conducted as assumed charged HB for two molecules (E_1), and complete interaction sphere of distinctive HB around one molecule (E_2), and as assumed neutral HB under the same conditions for E_1*, E_2*.*

Hydrogen Bond	E_1 [kJ mol^{-1}]	E_2 [kJ mol^{-1}]
N1-H7…O1	-54.83	-53.05
N1-H8…O1	-55.37	-55.37
N1-H9…O2	-47.52	-46.32
	E_1* [kJ mol^{-1}]	E_2* [kJ mol^{-1}]
N1-H7…O1	-30.71	-29.51
N1-H8…O1	-31.07	-31.07
N1-H9…O2	-25.80	-25.00

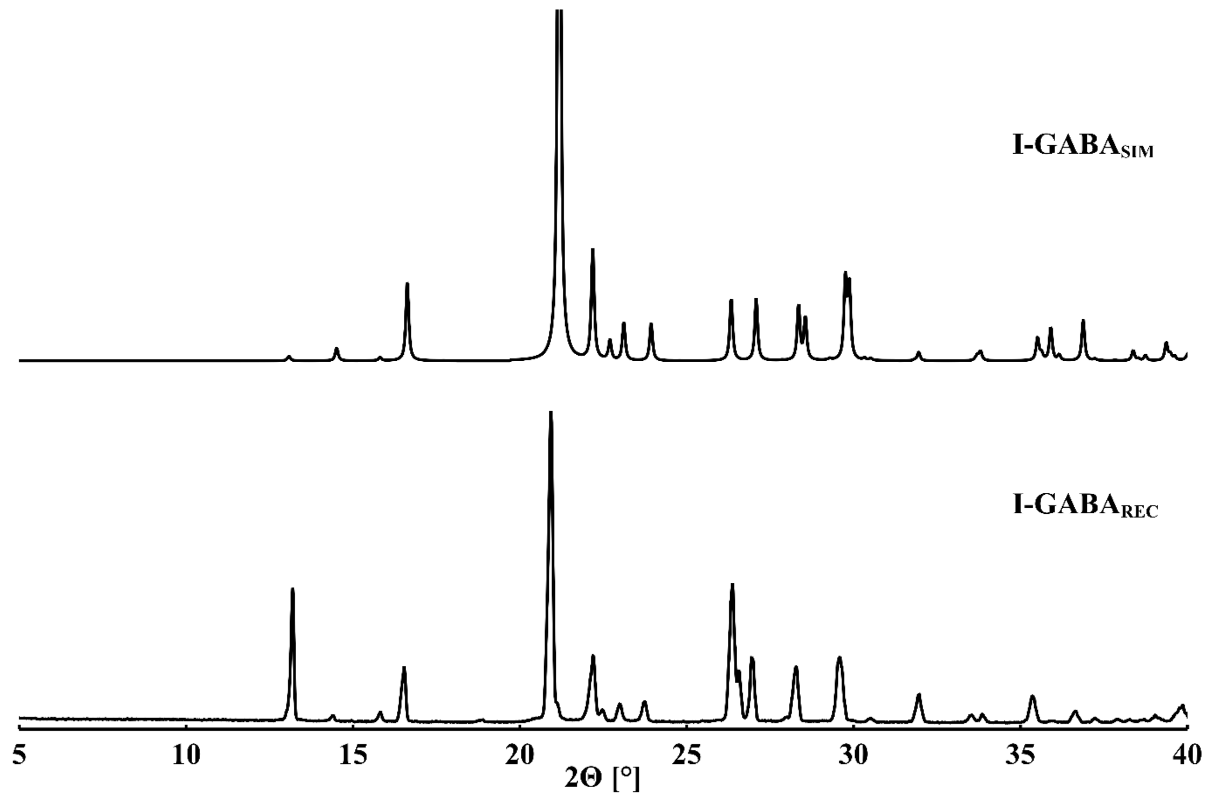

Figure S1. *Powder pattern comparison of **I**-GABA. Simulated from single crystal data (top), recorded substance (bottom). A range between 5° 2Θ and 40° 2Θ is depicted.*

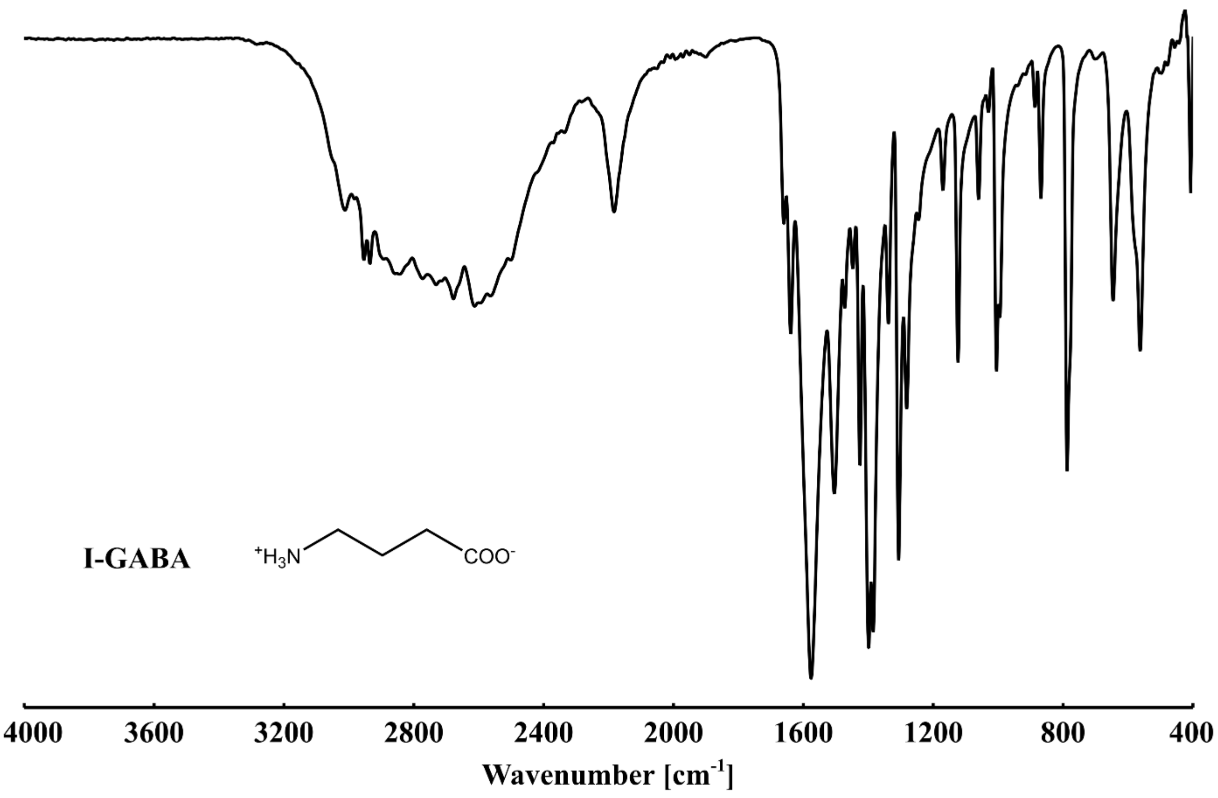

Figure S2. *IR spectrum of **I**-GABA, shown in a range between 4000 cm^{-1} – 400 cm^{-1}. Broad ammonium hydrogen bond network and C-H stretch band between 3200 cm^{-1} and 2270 cm^{-1}, carboxylate stretch band at 1610 cm^{-1}.*

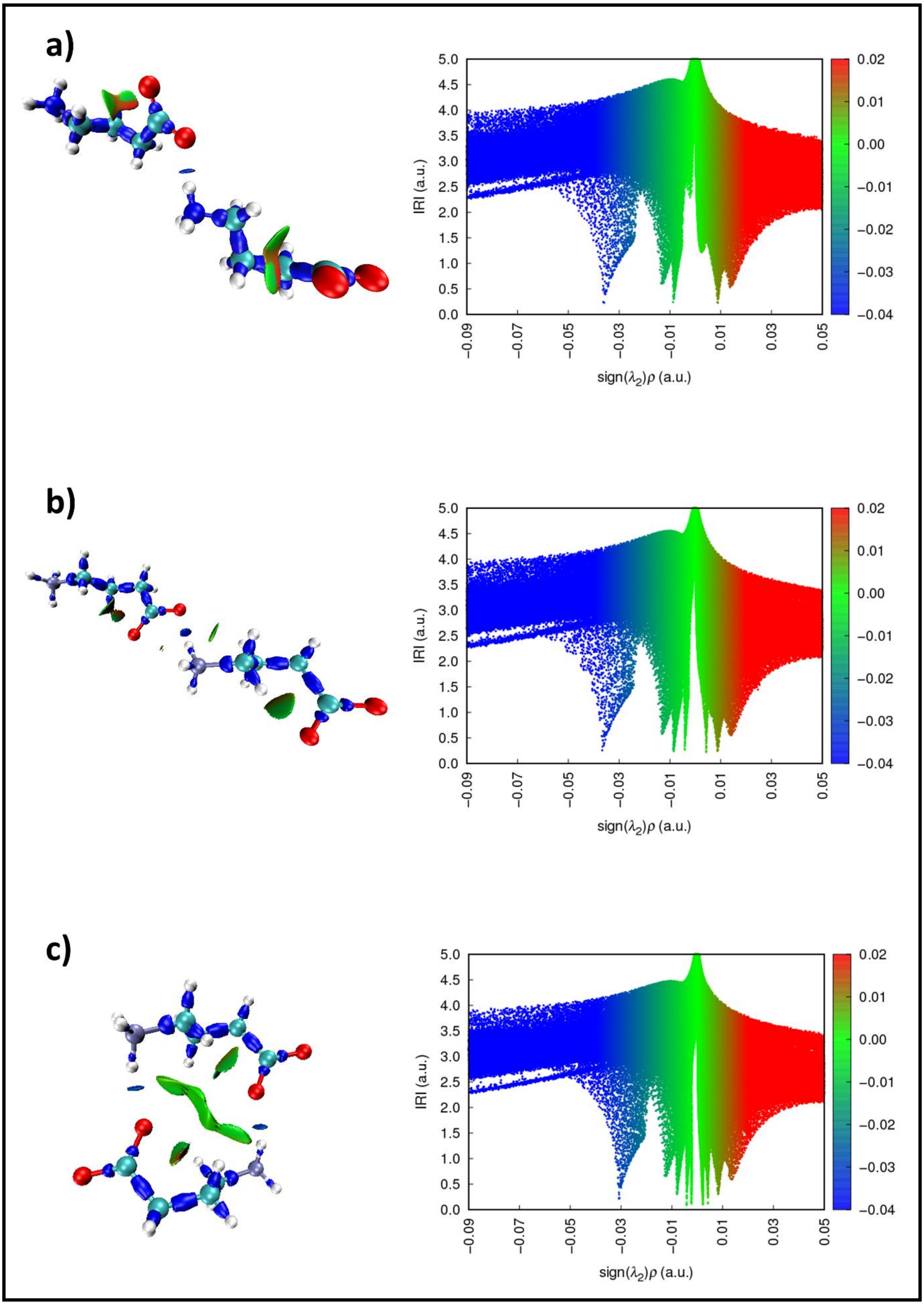

Figure S3. *Interaction Region Indicator surfaces and related scatter plots of the distinctive hydrogen bonds in* ***I****-GABA: N1-H7…O1 a), N1-H8…O1 b), and N1-H9…O2 c). Blue regions signify strong attraction, green regions weak attraction and red regions repulsion.*

II-GABA (II-1)

Table S3. *Crystallographic data for* ***II****-GABA.*

**Parameters**	**II-GABA**
**Formula**	$C_4 H_9 N O_2$
**Formula Moiety**	$C_4 H_9 N O_2$
**M_r [g mol^{-1}]**	103.12
**Temperature [K]**	140(2)
**System/space group**	tetragonal, *$I4_1cd$*
**a (Å)**	11.8658(4)
**b (Å)**	11.8658(4)
**c (Å)**	15.2642(6)
**α (°)**	90
**β (°)**	90
**γ (°)**	90
**V (Å^3)**	2149.16(17)
**Z/Z'**	16/1
**Density [g/cm^3]**	1.275
**μ [mm^{-1}]**	0.101
**T_{min}/T_{max}**	0.6874/0.7463
**F (000)**	896
**Crystal size [mm]**	0.12 · 0.17 · 0.26
**2θ range [°]**	3.4 – 31.8
**Completeness [%]**	99.2
**Recorded refl.**	6406
**Independent refl.**	1707
**Goodness-of-fit F^2**	1.058
**X-Ray Source**	Mo Kα (λ = 0.71073)
**Flack x**	-0.4(5)
**R_1 [%] /wR_2 [%] /S**	3.15/ 7.66/ 1.06

Table S4. *Distinctive energy values for the occurring HB obtained by AIM analysis via multiwfn conducted as assumed charged HB for two molecules (E_1), and complete interaction sphere of distinctive HB around one molecule (E_2), and as assumed neutral HB under the same conditions for E_1*, E_2*.*

Hydrogen Bond	E_1 [kJ mol^{-1}]	E_2 [kJ mol^{-1}]
N1-H7…O1	-42.87	-41.51
N1-H8…O1	-56.37	-55.02
N1-H9…O2	-48.88	-46.06
	E_1* [kJ mol^{-1}]	E_2* [kJ mol^{-1}]
N1-H7…O1	-22.67	-21.76
N1-H8…O1	-31.74	-30.83
N1-H9…O2	-25.37	-24.81

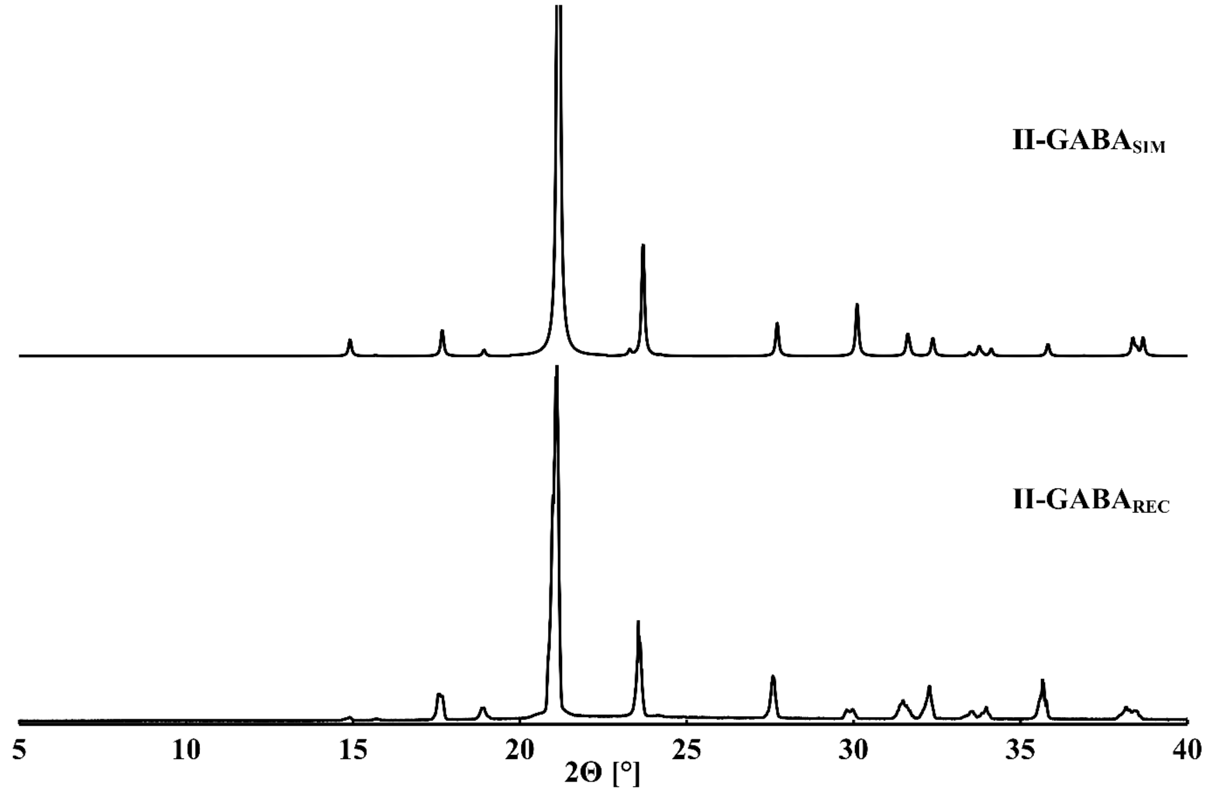

Figure S4. *Powder pattern comparison of* ***II****-GABA. Simulated from single crystal data (top), recorded substance (bottom). A range between 5° 2Θ and 40° 2Θ is depicted.*

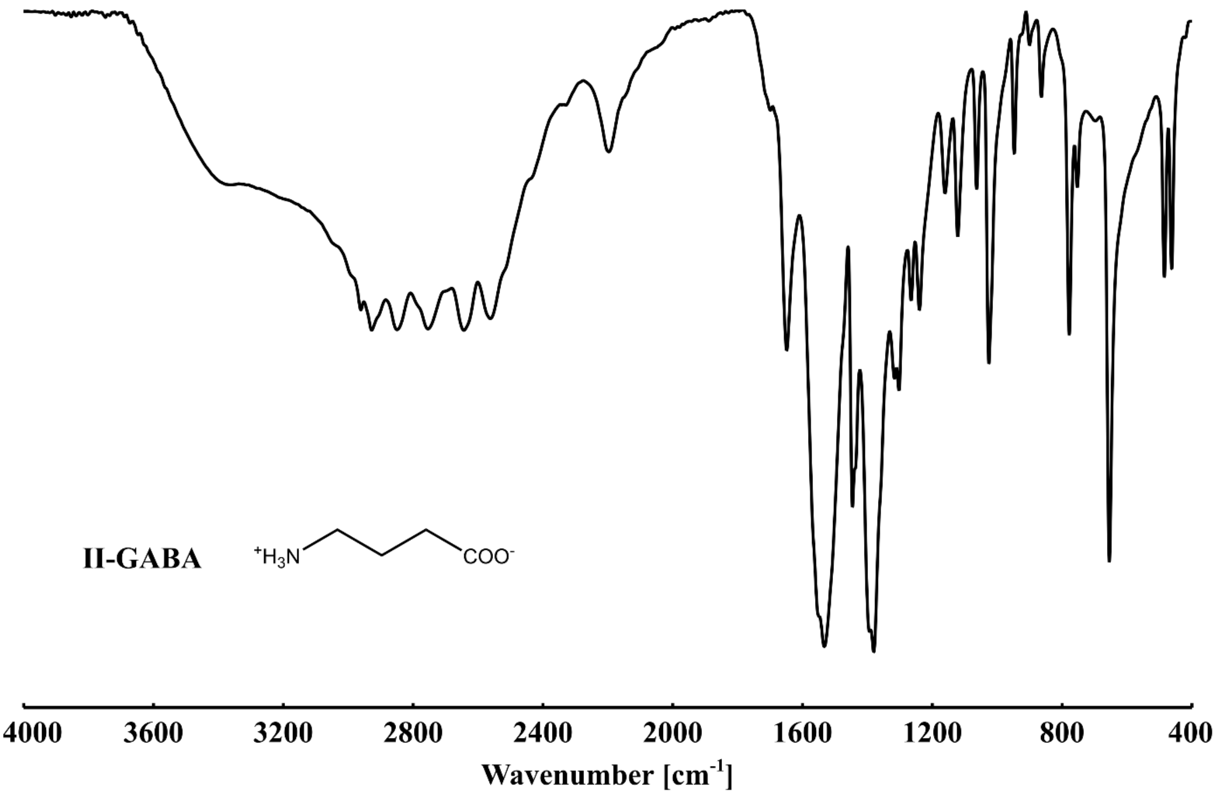

Figure S5. *IR spectrum of* ***II****-GABA, shown in a range between 4000 cm^{-1} – 400 cm^{-1}. Broad ammonium hydrogen bond network and C-H stretch band between 3700 cm^{-1} and 2270 cm^{-1}, carboxylate stretch band at 1649 cm^{-1}.*

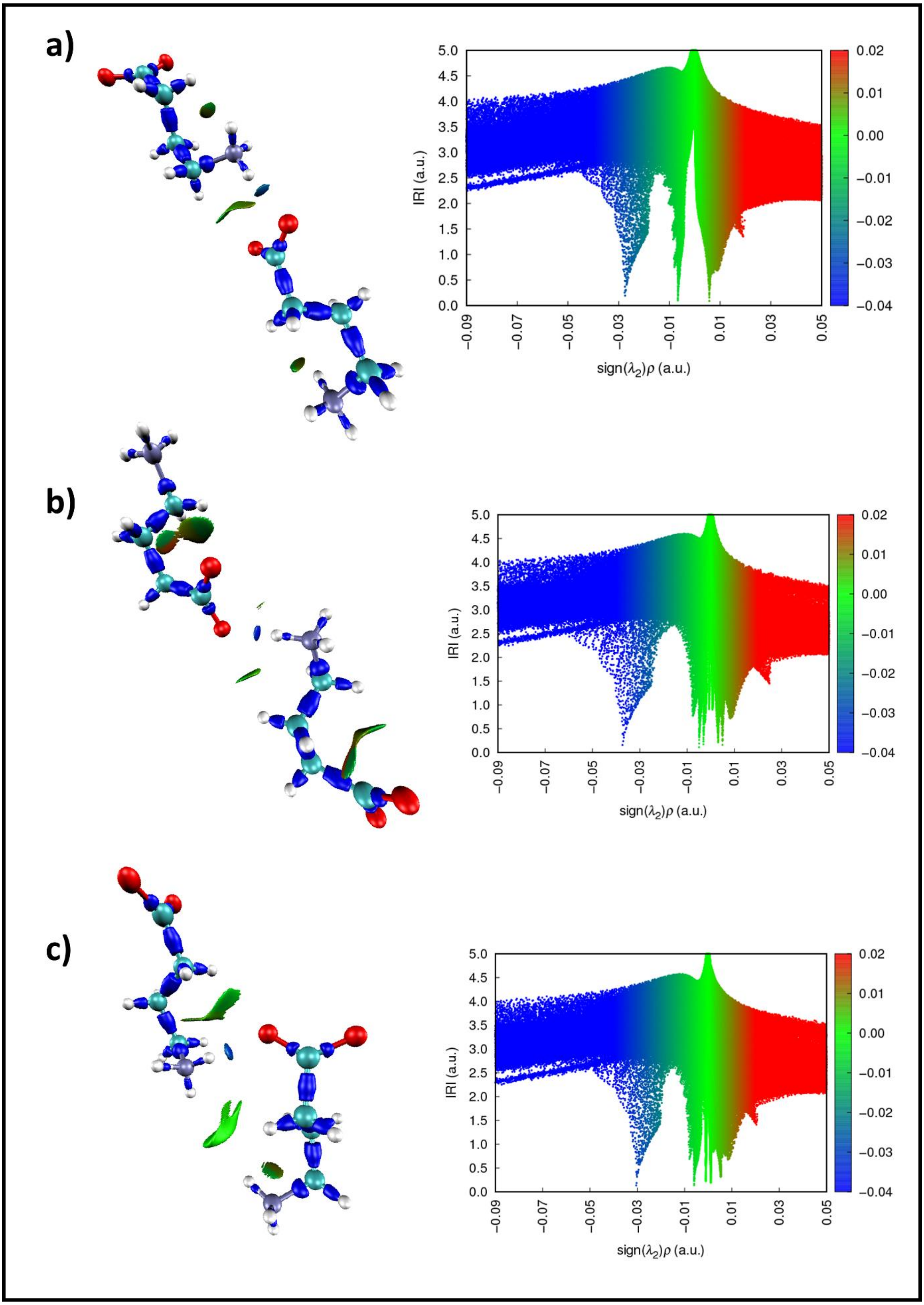

Figure S6. *Interaction Region Indicator surfaces and related scatter plots of the distinctive hydrogen bonds in* ***II****-GABA: N1-H7…O1 a), N1-H8…O1 b), and N1-H9…O2 c). Blue regions signify strong attraction, green regions weak attraction and red regions repulsion.*

II-Gabapentin (II-2)

Table S5. *Crystallographic data for **II**-gabapentin.*

**Parameters**	**II-gabapentin**
**Formula**	$C_9 H_{17} N O_2$
**Formula Moiety**	$C_9 H_{17} N O_2$
**M_r [g mol^{-1}]**	171.23
**Temperature [K]**	100.00(1)
**System/space group**	monoclinic, *$P2_1/c$*
**a (Å)**	5.8605(2)
**b (Å)**	6.9140(3)
**c (Å)**	22.1954(7)
**α (°)**	90
**β (°)**	89.888(3)
**γ (°)**	90
**V (Å^3)**	899.34(6)
**Z/Z'**	4/1
**Density [g/cm^3]**	1.265
**μ [mm^{-1}]**	0.712
**T_{min}/T_{max}**	0.745/0.865
**F (000)**	376
**Crystal size [mm]**	0.21 · 0.26 · 0.44
**2θ range [°]**	6.7 – 76.7
**Completeness [%]**	99.7
**Recorded refl.**	5300
**Independent refl.**	1591
**Goodness-of-fit F^2**	1.068
**X-Ray Source**	Cu Kα (λ = 1.54184)
**R_1 [%] /wR_2 [%] /S**	3.44/ 9.36/ 1.07

Table S6. *Distinctive energy values for the occurring HB obtained by AIM analysis via multiwfn conducted as assumed charged HB for two molecules (E_1), and complete interaction sphere of distinctive HB around one molecule (E_2), and as assumed neutral HB under the same conditions for E_1*, E_2*.*

Hydrogen Bond	E_1 [kJ mol^{-1}]	E_2 [kJ mol^{-1}]
N1-H5…O1	-55.48	-54.33
N1-H6…O1	-48.73	-47.52
N1-H7…O2	-52.97	-51.74
	E_1* [kJ mol^{-1}]	E_2* [kJ mol^{-1}]
N1-H5…O1	-31.14	-30.37
N1-H6…O1	-26.61	-25.79
N1-H7…O2	-29.45	-28.63

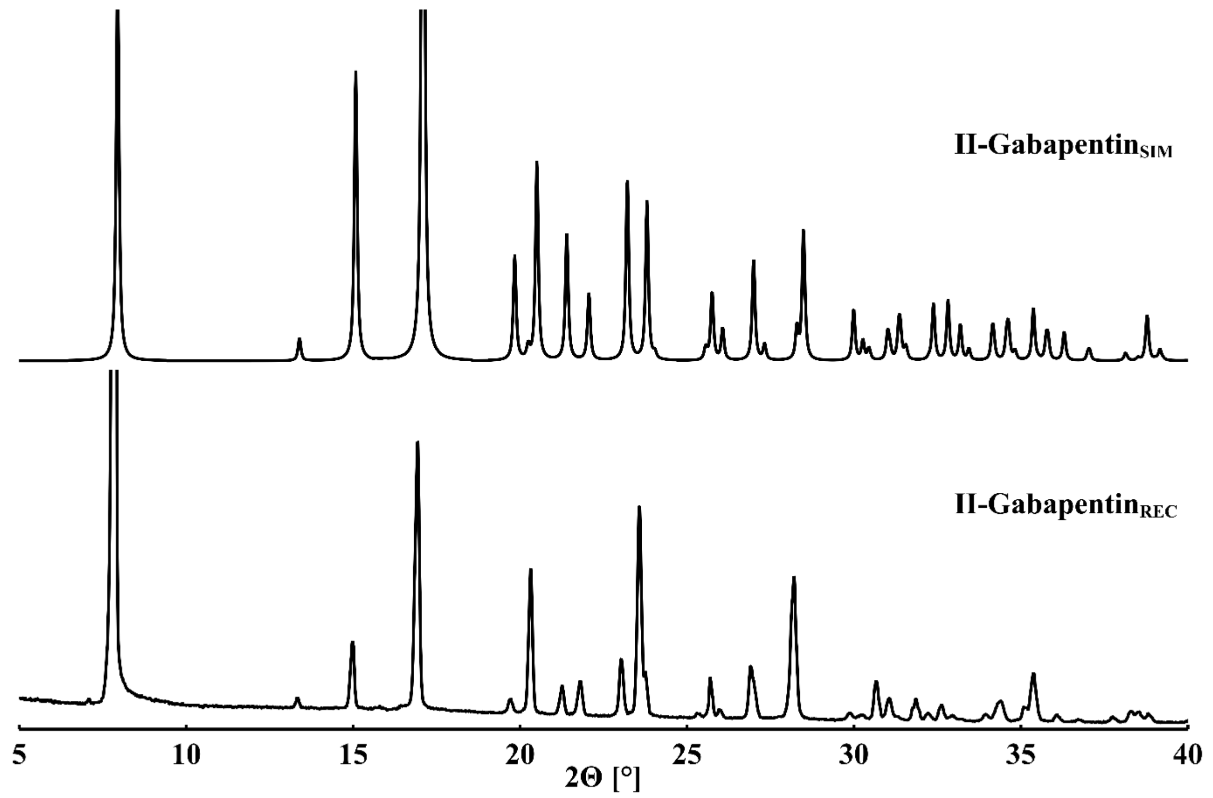

*Figure S7. Powder pattern comparison of **II**-gabapentin. Simulated from single crystal data (top), recorded substance (bottom). A range between 5° 2Θ and 40° 2Θ is depicted.*

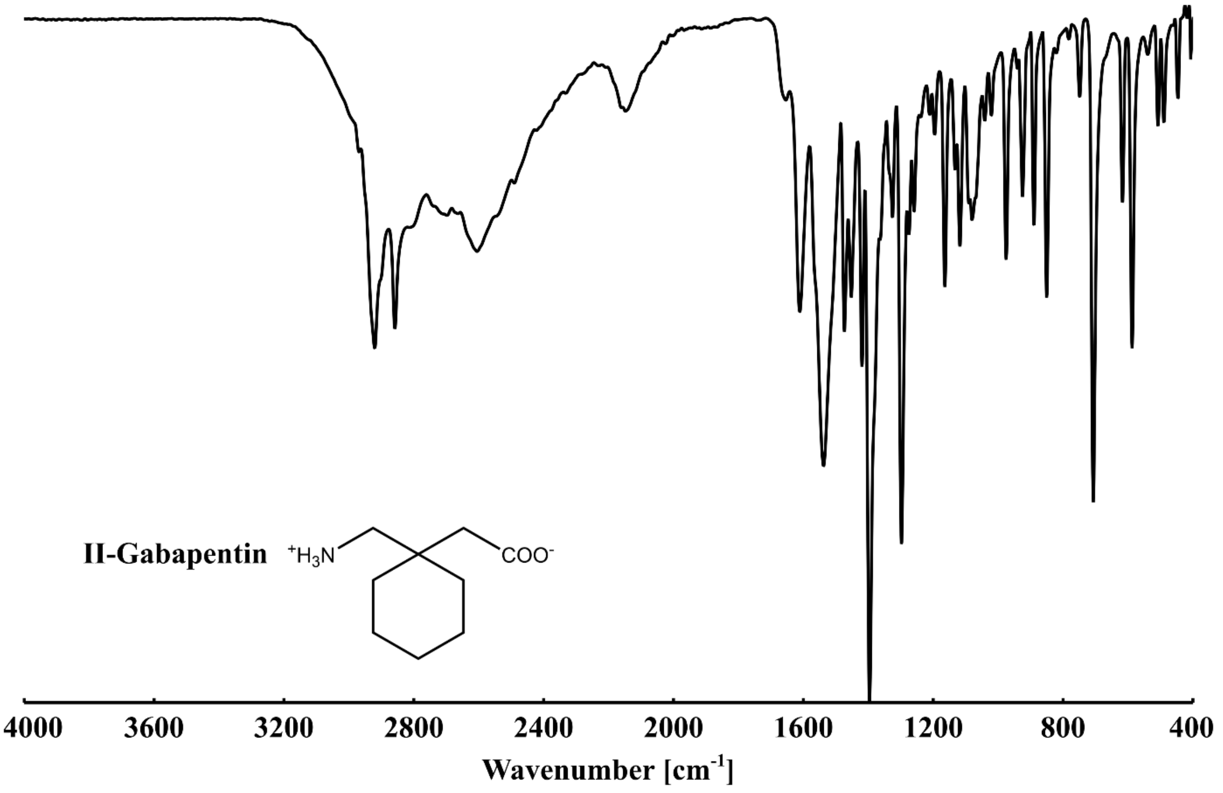

*Figure S8. IR spectrum of **II**-gabapentin, shown in a range between 4000 cm^{-1} – 400 cm^{-1}. Broad ammonium hydrogen bond network and C-H stretch band between 3200 cm^{-1} and 2240 cm^{-1}, carboxylate band at 1611 cm^{-1}.*

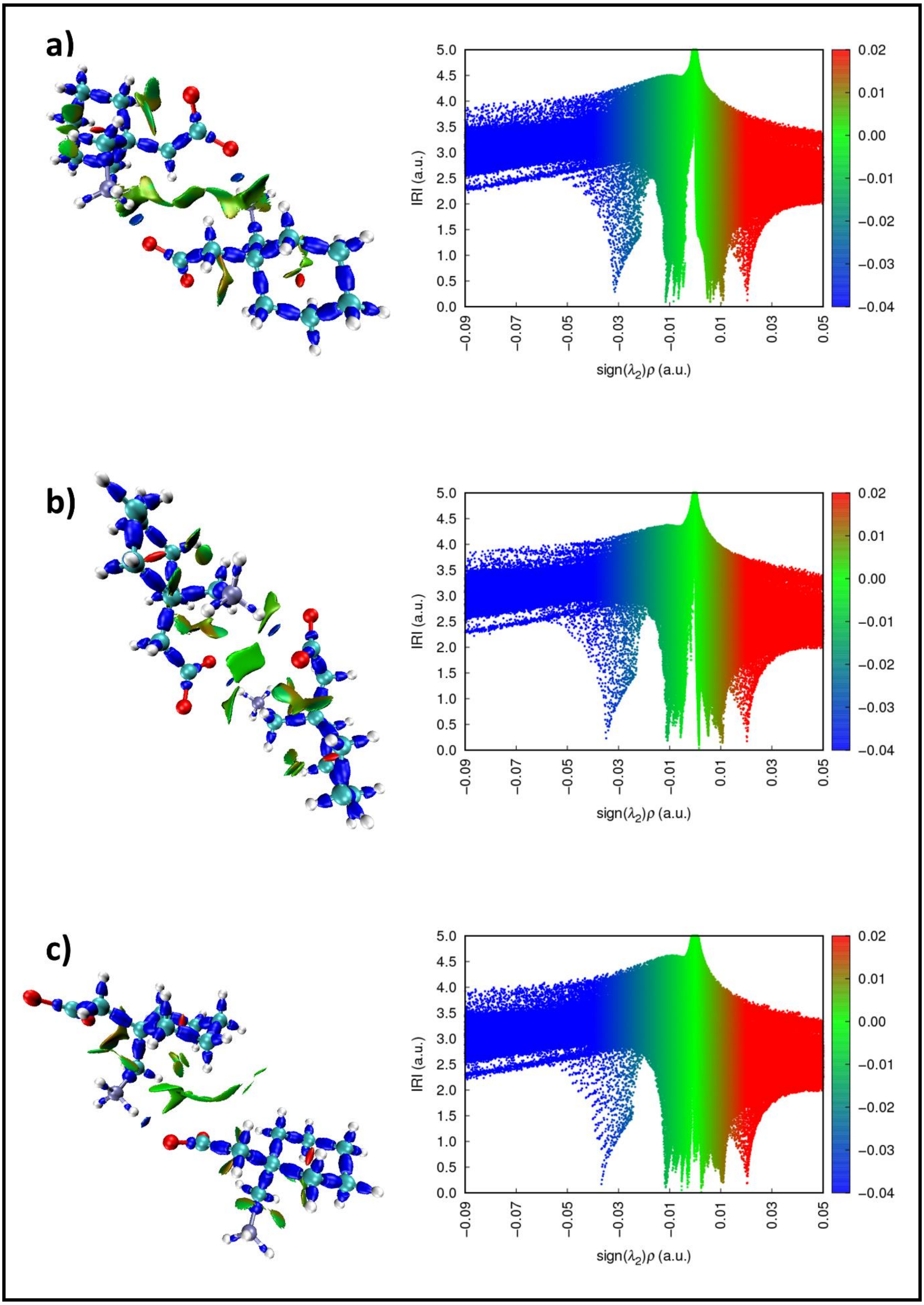

Figure S9. *Interaction Region Indicator surfaces and related scatter plots of the distinctive hydrogen bonds in **II**-gabapentin: HB N1-H5…O1 a), N1-H6…O1 b), and N1-H7…O2 c). Blue regions signify strong attraction, green regions weak attraction and red regions repulsion.*

IV-Gabapentin (IV-2)

Table S7. *Crystallographic data for **IV**-gabapentin.*

Parameters	IV-gabapentin
**Formula**	$C_9 H_{17} N O_2$
**Formula Moiety**	$C_9 H_{17} N O_2$
**M_r [g mol^{-1}]**	171.23
**Temperature [K]**	100.00(1)
**System/space group**	monoclinic, *C2/c*
**a (Å)**	31.3806(7)
**b (Å)**	5.8999(1)
**c (Å)**	10.7957(2)
**α (°)**	90
**β (°)**	113.041(3)
**γ (°)**	90
**V (Å^3)**	1839.29(7)
**Z/Z'**	8/1
**Density [g/cm^3]**	1.237
**μ [mm^{-1}]**	0.696
**T_{min}/T_{max}**	0.921/0.979
**F (000)**	752
**Crystal size [mm]**	0.03 · 0.08 · 0.12
**2θ range [°]**	3.1 – 77.3
**Completeness [%]**	100
**Recorded refl.**	10650
**Independent refl.**	1641
**Goodness-of-fit F^2**	1.084
**X-Ray Source**	Cu Kα (λ = 1.54184)
**R_1 [%] /wR_2 [%] /S**	2.81/ 6.97/ 1.08

Table S8. *Distinctive energy values for the occurring HB obtained by AIM analysis via multiwfn conducted as assumed charged HB for two molecules (E_1), and complete interaction sphere of distinctive HB around one molecule (E_2), and as assumed neutral HB under the same conditions for E_1*, E_2*.*

Hydrogen Bond	E_1 [kJ mol^{-1}]	E_2 [kJ mol^{-1}]
N1-H5…O1	-53.87	-52.67
N1-H6…O1	-47.05	-45.83
N1-H7…O2	-54.65	-53.62
	E_1* [kJ mol^{-1}]	E_2* [kJ mol^{-1}]
N1-H5…O1	-30.06	-29.26
N1-H6…O1	-25.48	-24.66
N1-H7…O2	-30.58	-29.89

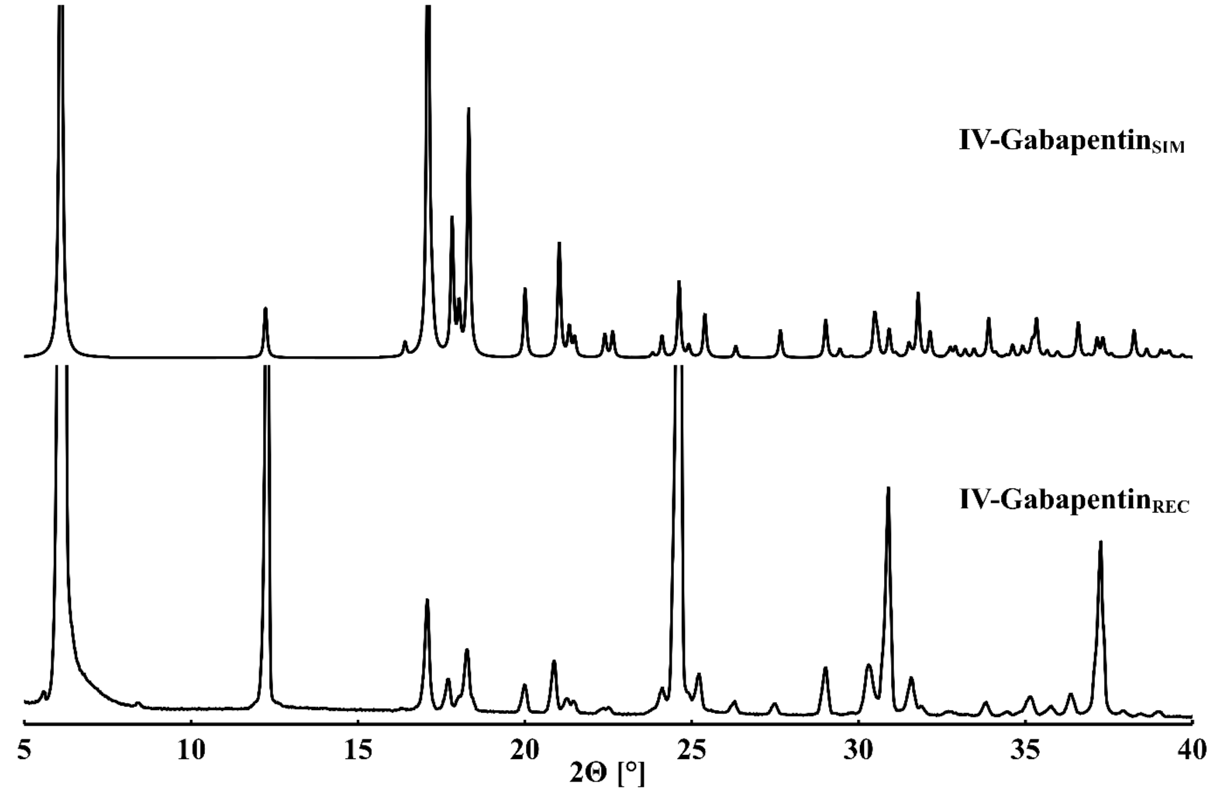

Figure S10. *Powder pattern comparison of **IV**-gabapentin. Simulated from single crystal data (top), recorded substance (bottom). A range between 5° 2Θ and 40° 2Θ is depicted.*

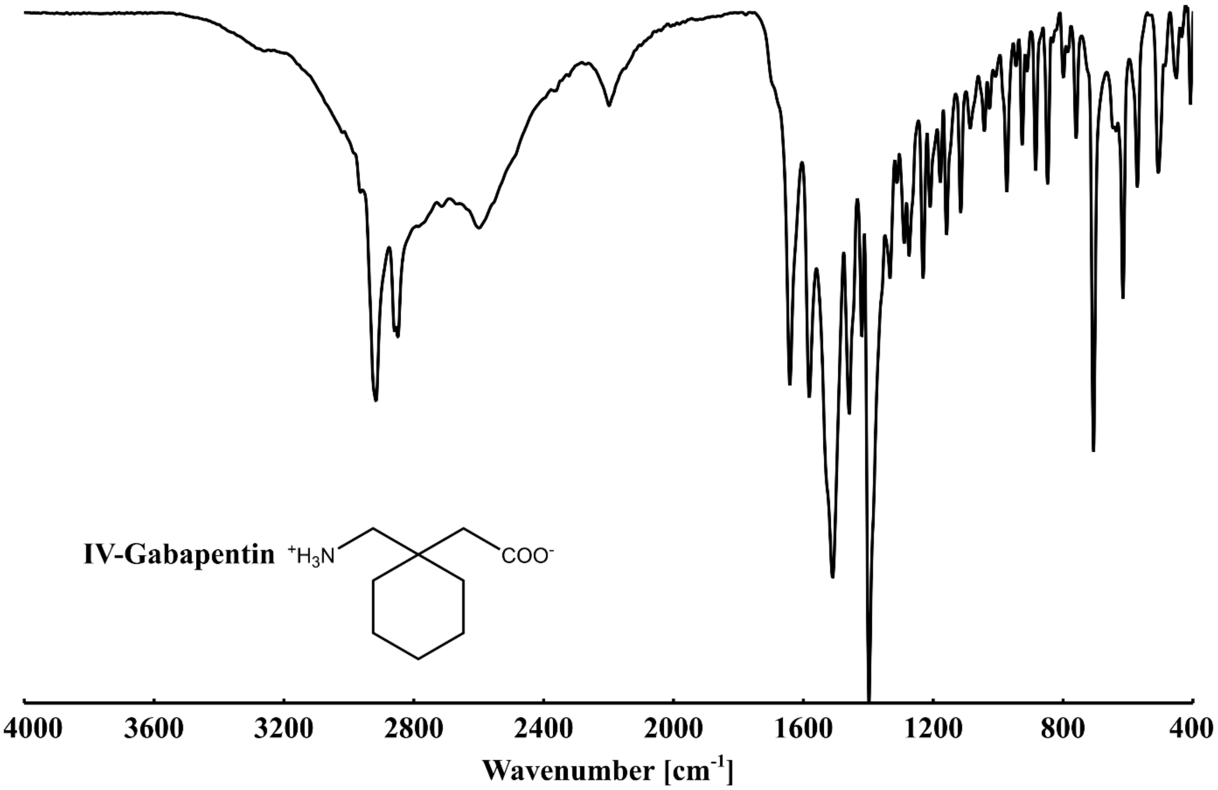

Figure S11. *IR spectrum of **IV**-gabapentin, shown in a range between 4000 cm^{-1} – 400 cm^{-1}. Broad ammonium hydrogen bond network and C-H stretch band between 3500 cm^{-1} and 2270 cm^{-1}, carboxylate band at 1641 cm^{-1}.*

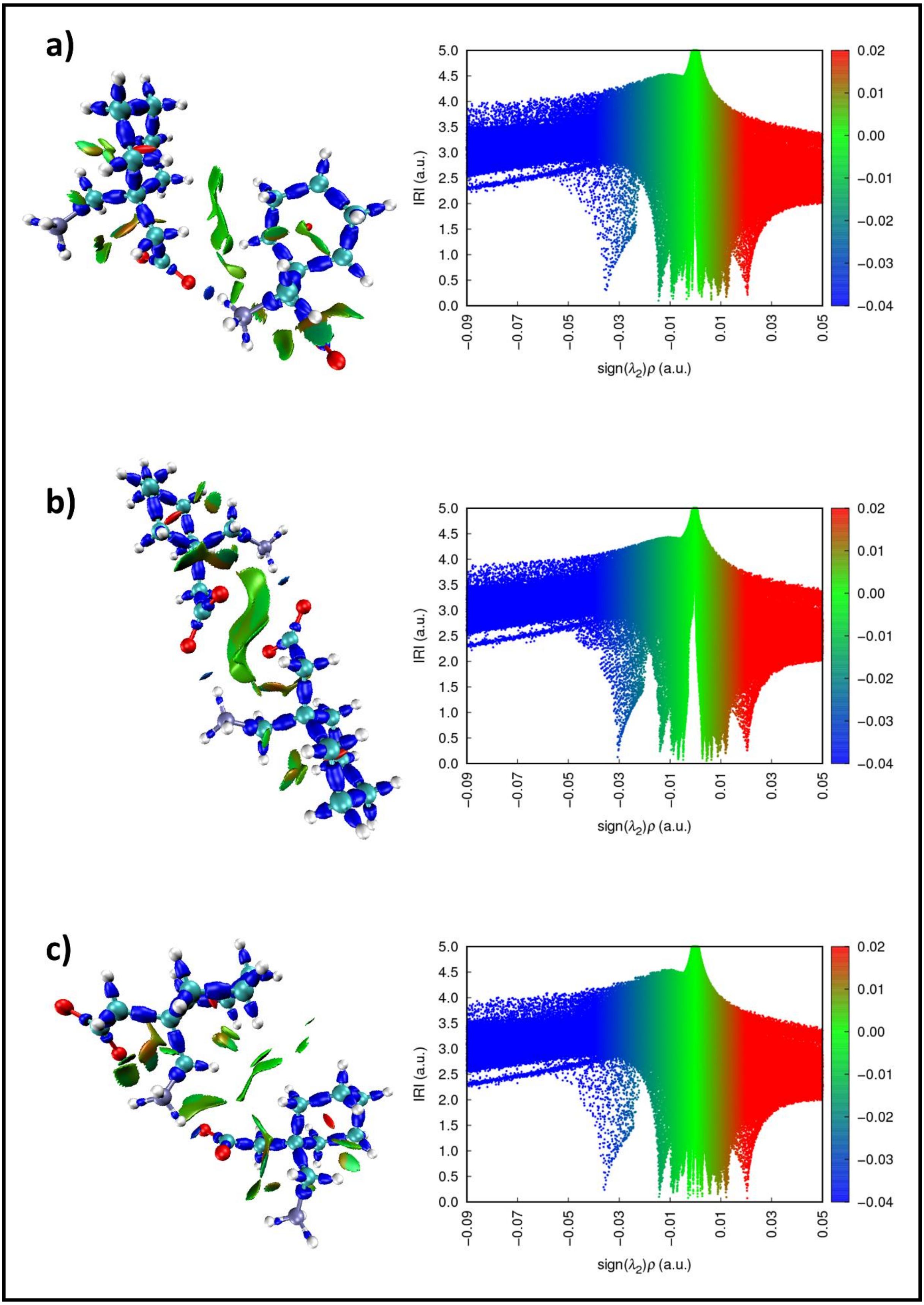

Figure S12. *Interaction Region Indicator surfaces and related scatter plots of the distinctive hydrogen bonds in* ***IV****-gabapentin: N1-H5…O1 a), N1-H6…O1 b), and N1-H7…O2 c). Blue regions signify strong attraction, green regions weak attraction and red regions repulsion.*

Gabapentin • H_2O (I-Gabapentin) (I-2)

Table S9. *Crystallographic data for gabapentin • H_2O (**I**-gabapentin).*

Parameters	gabapentin • H_2O (I-gabapentin)
**Formula**	$C_9 H_{19} N O_3$
**Formula Moiety**	$C_9 H_{17} N O_2$, $H_2 O$
**M_r [g mol^{-1}]**	189.25
**Temperature [K]**	100.01(1)
**System/space group**	orthorhombic, *Pbca*
**a (Å)**	9.2188(3)
**b (Å)**	7.6384(3)
**c (Å)**	29.0462(12)
**α (°)**	90
**β (°)**	90
**γ (°)**	90
**V (Å^3)**	2045.34(13)
**Z/Z'**	8/1
**Density [g/cm^3]**	1.229
**μ [mm^{-1}]**	0.746
**T_{min}/T_{max}**	0.902/1.000
**F (000)**	832
**Crystal size [mm]**	0.02 · 0.06 · 0.08
**2θ range [°]**	3.0 – 77.9
**Completeness [%]**	99.8
**Recorded refl.**	7406
**Independent refl.**	1982
**Goodness-of-fit F^2**	1.062
**X-Ray Source**	Cu Kα (λ = 1.54184)
**R_1 [%] /wR_2 [%] /S**	4.91/ 13.85/ 1.06

Table S10. *Distinctive energy values for the occurring HB obtained by AIM analysis via multiwfn conducted as assumed charged HB for two molecules (E_1), and complete interaction sphere of distinctive HB around one molecule (E_2), and as assumed neutral HB under the same conditions for E_1*, E_2*.*

Hydrogen Bond	E_1 [kJ mol^{-1}]	E_2 [kJ mol^{-1}]
N1-H5…O1	-66.71	-66.55
N1-H6…O1	-51.46	-41.46
N1-H7…O3	-37.01	-37.86
N1-H7…O3	-16.67	-16.81
O3-H18…O2	-48.70	-49.10
O3-H19…O2	-49.12	-49.78
	E_1* [kJ mol^{-1}]	E_2* [kJ mol^{-1}]
N1-H5…O1	-38.68	-38.57
N1-H6…O1	-28.44	-21.73
N1-H7…O3	-18.75	-19.31
N1-H7…O3	-5.09	-5.18
O3-H18…O2	-26.59	-26.86
O3-H19…O2	-26.87	-27.31

Table S11. *Overview on the strong, distinctive hydrogen bonds in gabapentin monohydrate. The proton acceptor distance, the donor acceptor distance, the bond angle and the binding energy calculated for charged HBs are shown.*

**I-2**	H…A [Å]	D…A [Å]	D - H…A [°]	E_{bond} [kJ mol^{-1}]
N1-H6…O1	1.73(2)	2.752(2)	173(2)	-66.71
N1-H5…O1	1.88(3)	2.842(2)	166(2)	-51.46
O3-H19…O2	1.82(3)	2.752(2)	176(2)	-49.12
O3-H18…O2	1.85(3)	2.746(2)	170(3)	-48.70
N1-H7…O3	1.99(3)	2.802(2)	152(3)	-37.01
N1-H7…O3	2.53(3)	3.051(2)	118(2)	-16.67

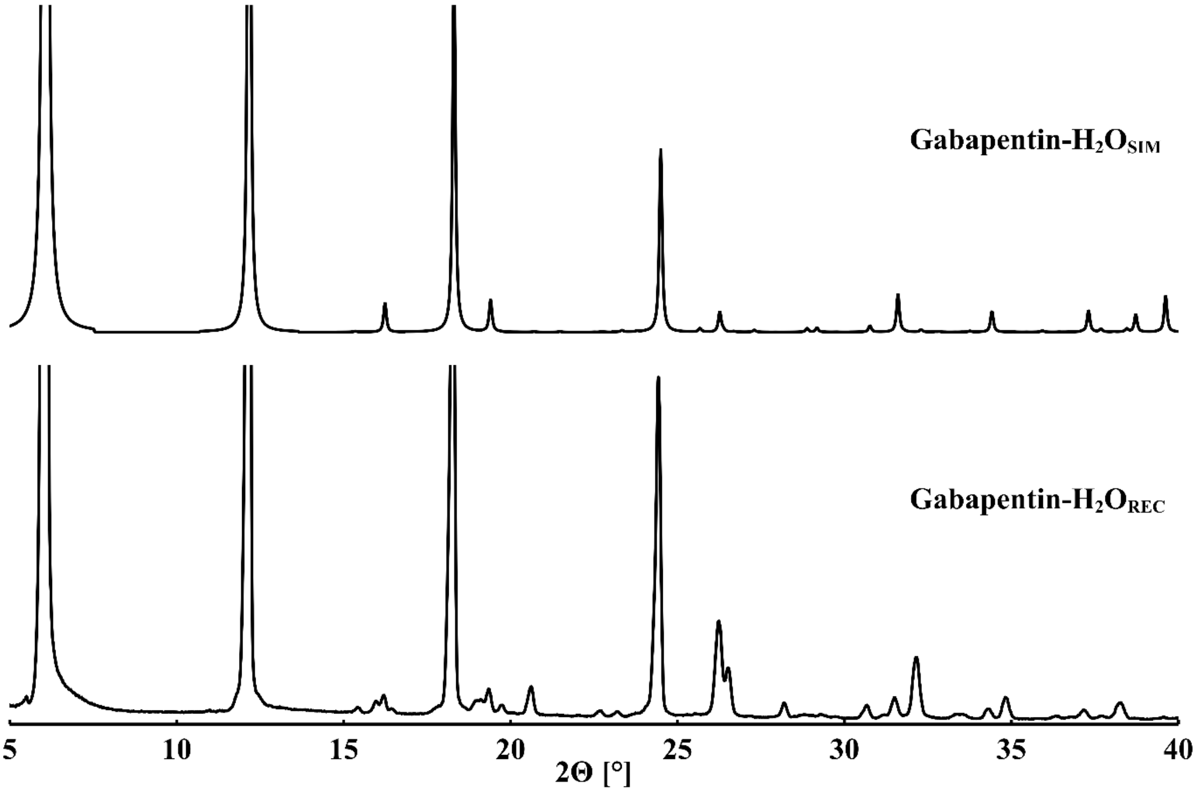

Figure S13. *Powder pattern comparison of **I**-gabapentin. Simulated from single crystal data (top), recorded substance (bottom). A range between 5° 2Θ and 40° 2Θ is depicted.*

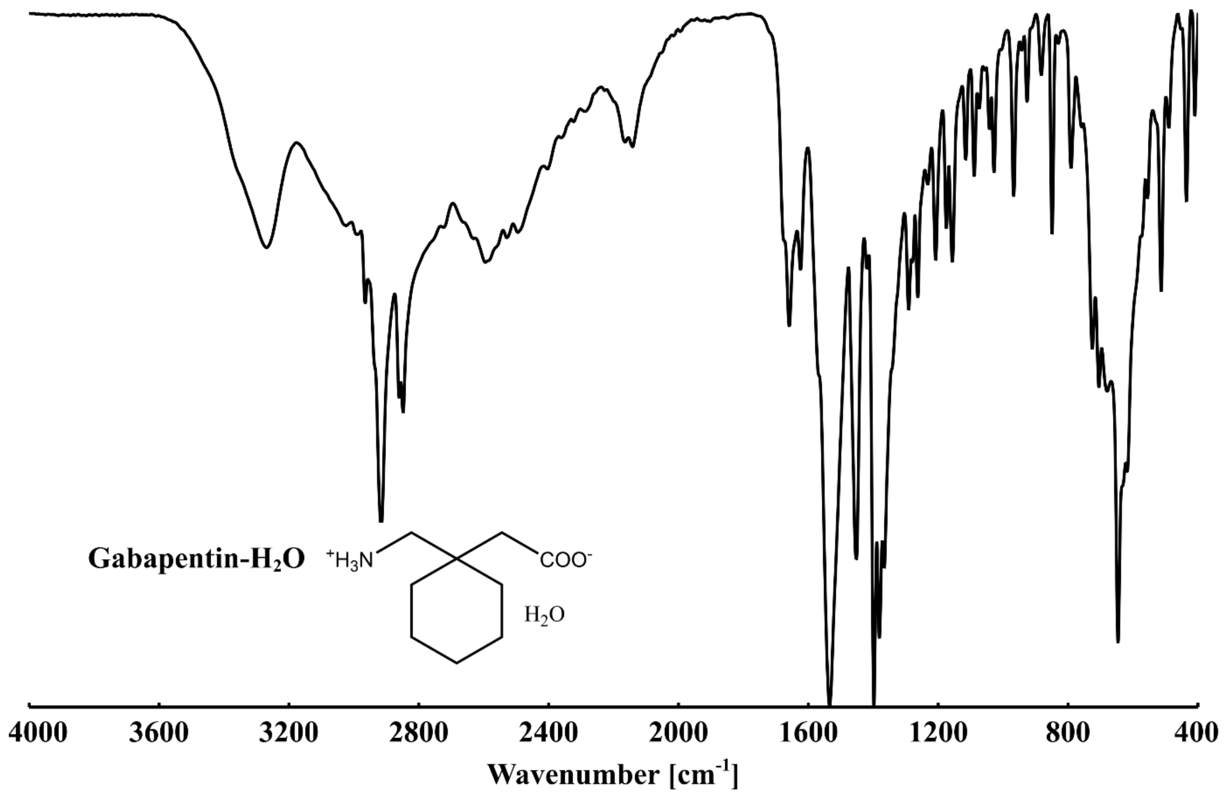

Figure S14. *IR spectrum of **I**-gabapentin, shown in a range between 4000 cm^{-1} – 400 cm^{-1}. Broad ammonium hydrogen bond network and C-H stretch band between 3625 cm^{-1} and 2240 cm^{-1}, water band at 3270 cm^{-1} and carboxylate band at 1659 cm^{-1}.*

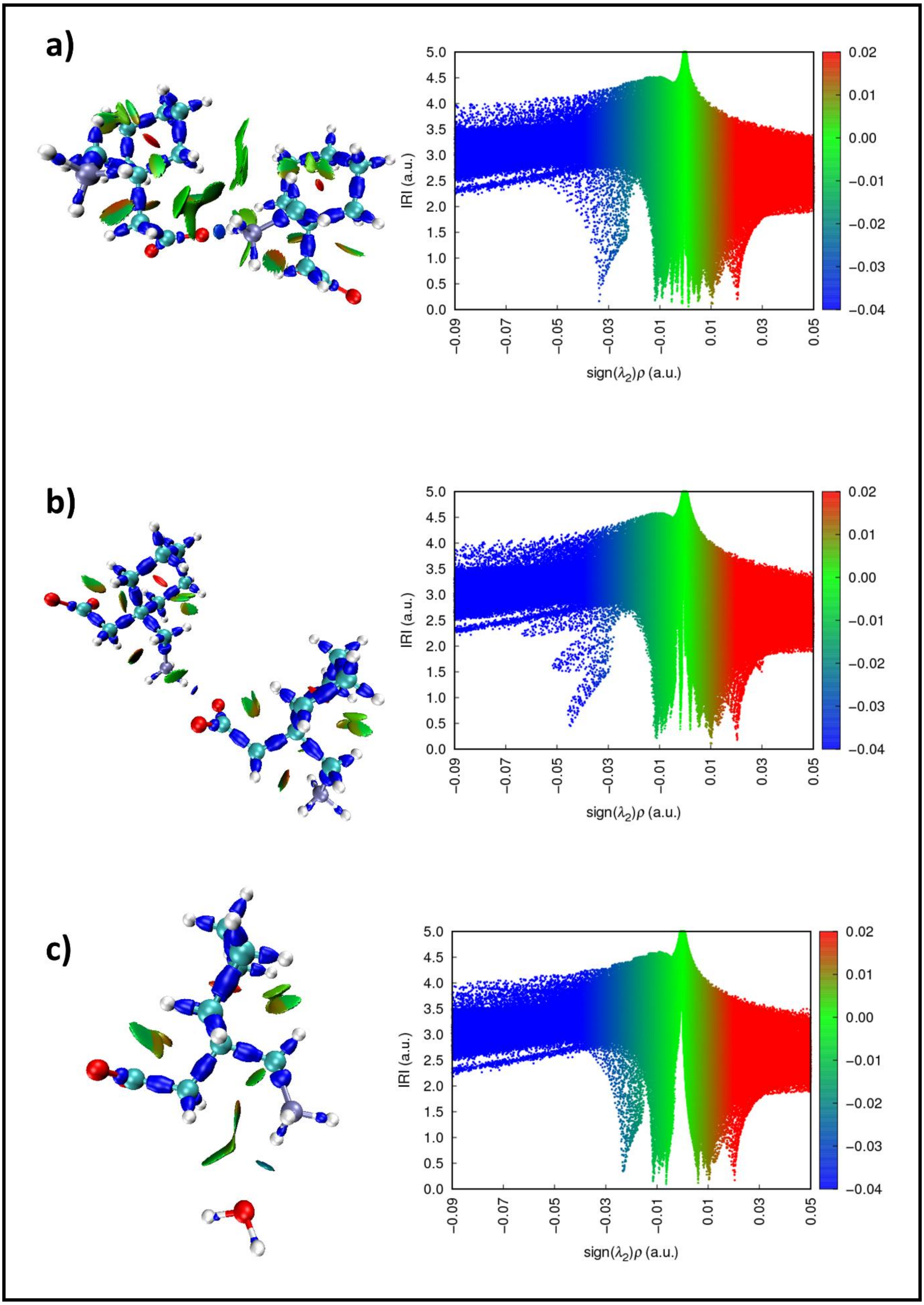

Figure S15. *Interaction Region Indicator surfaces and related scatter plots of three distinctive hydrogen bonds in* ***I****-gabapentin: HB N1-H5…O1 a), N1-H6…O1 b), and N1-H7…O3 c). Blue regions signify strong attraction, green regions weak attraction and red regions repulsion.*

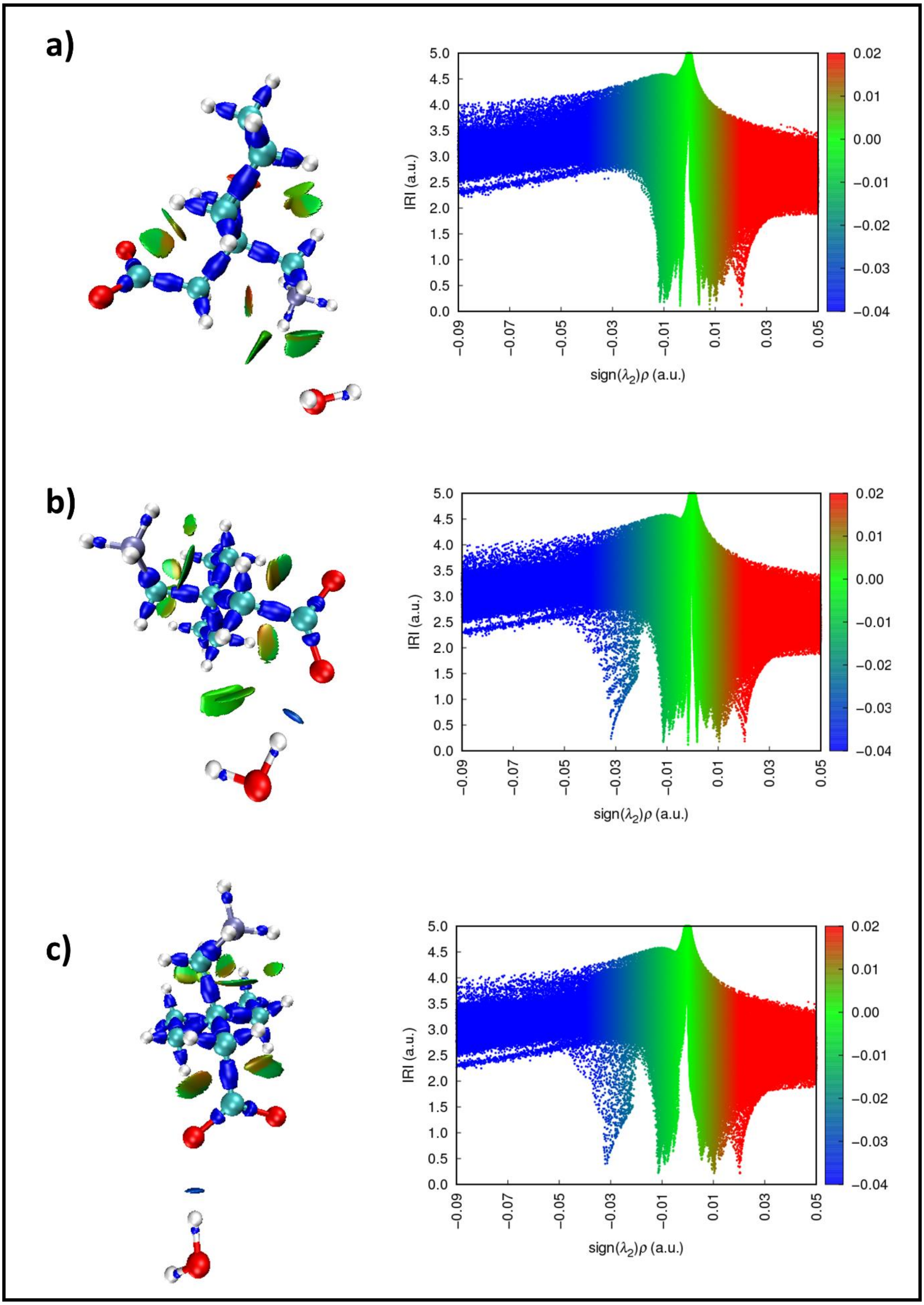

***Figure S16**. Interaction Region Indicator surfaces and related scatter plots of three distinctive hydrogen bonds in **I**-gabapentin: HB N1-H7…O3 a), O3-H18…O2 b), and O3-H19…O2 c). Blue regions signify strong attraction, green regions weak attraction and red regions repulsion.*

GABA Fumarate (2:1) (1-3)

***Table S11.** Crystallographic data for GABA fumarate (2:1).*

**Parameters**	**GABA fumarate (2:1)**
**Formula**	$C_{12} H_{22} N_2 O_8$
**Formula Moiety**	$2(C_4 H_{10} N O_2), C_4 H_2 O_4$
**M_r [g mol^{-1}]**	322.31
**Temperature [K]**	100.00(1)
**System/space group**	monoclinic, *$P2_1/n$*
**a (Å)**	7.2196(8)
**b (Å)**	6.5760(1)
**c (Å)**	14.4204(2)
**α (°)**	90
**β (°)**	98.617(1)
**γ (°)**	90
**V (Å^3)**	785.562(19)
**Z/Z'**	2/1
**Density [g/cm^3]**	1.363
**μ [mm^{-1}]**	0.986
**T_{min}/T_{max}**	0.558/0.866
**F (000)**	344
**Crystal size [mm]**	0.15 · 0.39 · 0.67
**2θ range [°]**	5.3 – 77.7
**Completeness [%]**	99.7
**Recorded refl.**	4875
**Independent refl.**	1504
**Goodness-of-fit F^2**	1.067
**X-Ray Source**	Cu Kα (λ = 1.54184)
**R_1 [%] /wR_2 [%] /S**	3.23/ 8.32/ 1.07

***Table S12.** Distinctive energy values for the occurring HB obtained by AIM analysis via multiwfn conducted as assumed charged HB for two molecules (E_1), and complete interaction sphere of distinctive HB around one molecule (E_2), and as assumed neutral HB under the same conditions for E_1*, E_2*.*

Hydrogen Bond	E_1 [kJ mol^{-1}]	E_2 [kJ mol^{-1}]
N1-H8…O3	-48.84	-43.92
N1-H9…O4	-49.63	-44.40
N1-H10…O4	-44.02	-41.46
O1-H1…O3	-65.80	-62.72
	E_1* [kJ mol^{-1}]	E_2* [kJ mol^{-1}]
N1-H8…O3	-26.69	-23.38
N1-H9…O4	-27.21	-23.70
N1-H10…O4	-23.45	-21.73
O1-H1…O3	-38.07	-36.00

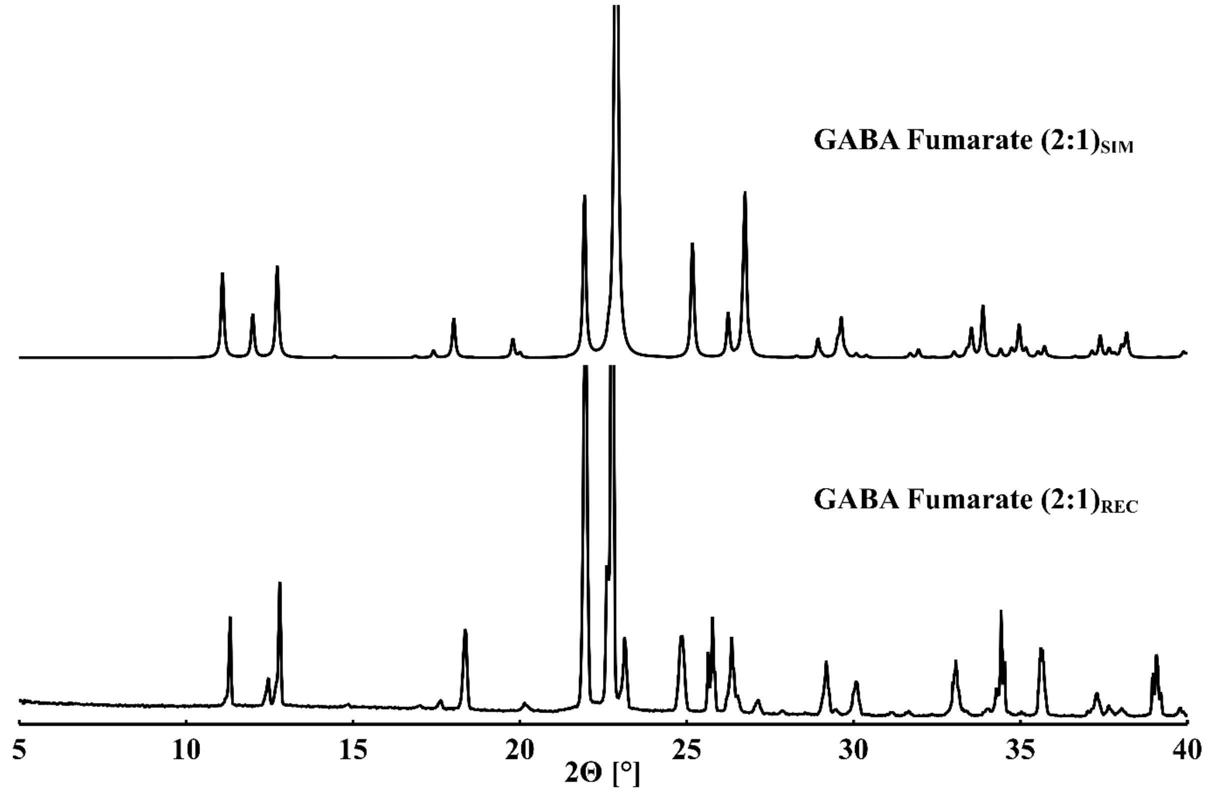

Figure S17. *Powder pattern comparison of GABA fumarate (2:1). Simulated from single crystal data (top), recorded substance (bottom). A range between 5° 2Θ and 40° 2Θ is depicted.*

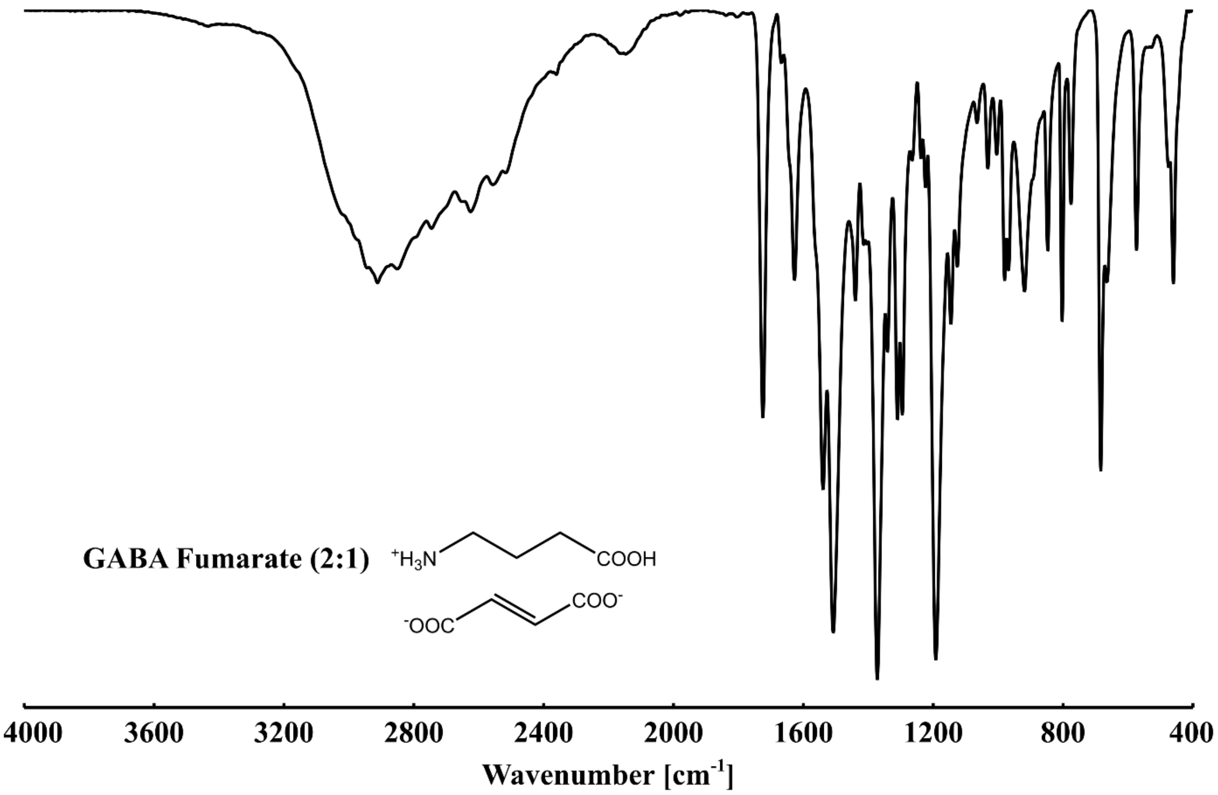

Figure S18. *IR spectrum of GABA fumarate (2:1), shown in a range between 4000 cm^{-1} – 400 cm^{-1}. Broad ammonium hydrogen bond network and C-H stretch band between 3640 cm^{-1} and 2240 cm^{-1}, carboxyl band at 1724 cm^{-1} carboxylate band at 1626 cm^{-1}.*

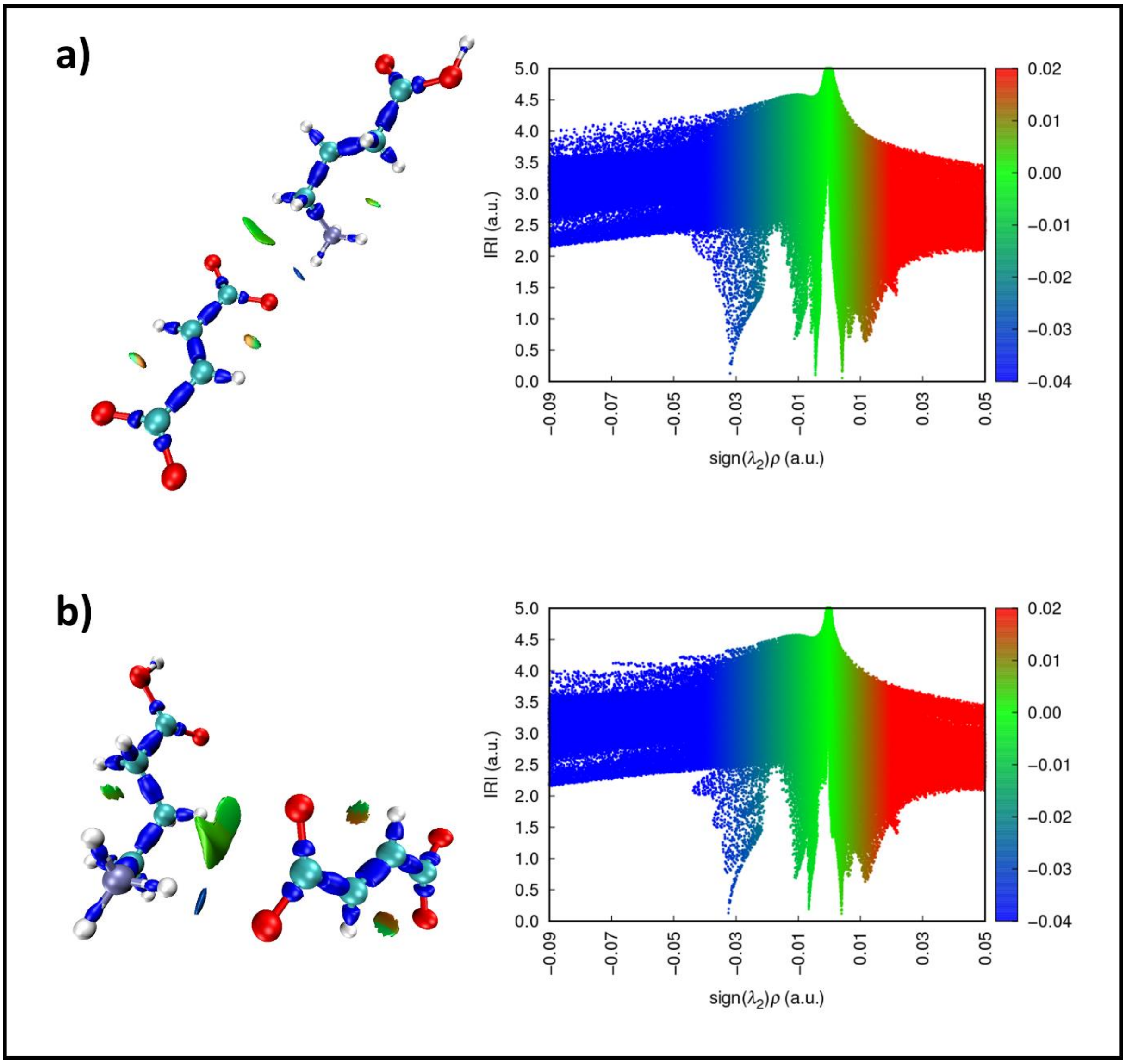

Figure S19. *Interaction Region Indicator surfaces and related scatter plots of two distinctive hydrogen bonds in GABA fumarate (2:1): N1-H8…O3 a), and N1-H9…O4 b). Blue regions signify strong attraction, green regions weak attraction and red regions repulsion.*

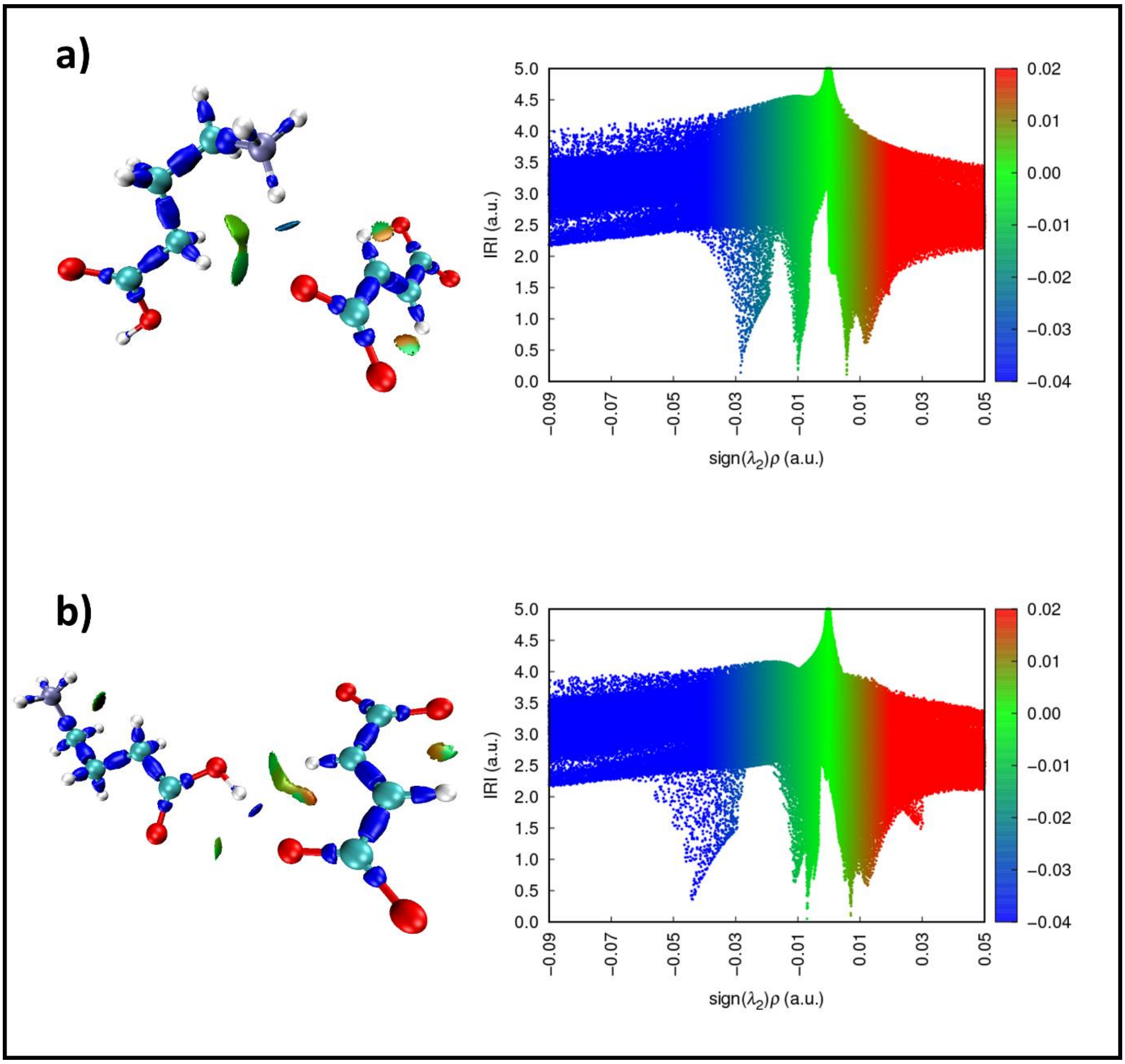

Figure S20. *Interaction Region Indicator surfaces and related scatter plots of two distinctive hydrogen bonds in GABA fumarate (2:1): N1-H10…O4 a), and O1-H1…O3 b). Blue regions signify strong attraction, green regions weak attraction and red regions repulsion.*

Gabapentin Fumarate (2:1) (2-3a)

Table S14. *Crystallographic data for gabapentin fumarate (2:1).*

Parameters	gabapentin fumarate (2:1)
**Formula**	$C_{11} H_{19} N O_4$
**Formula Moiety**	$C_9 H_{17} N O_2$, 0.5($C_4 H_2 O_4$)
**M_r [g mol^{-1}]**	229.27
**Temperature [K]**	100.00(1)
**System/space group**	monoclinic, *$P2_1/c$*
**a (Å)**	14.1264(6)
**b (Å)**	7.4699(2)
**c (Å)**	12.2122(4)
**α (°)**	90
**β (°)**	105.751(4)
**γ (°)**	90
**V (Å^3)**	1240.28(8)
**Z/Z'**	4/1
**Density [g/cm^3]**	1.228
**μ [mm^{-1}]**	0.771
**T_{min}/T_{max}**	0.920/0.970
**F (000)**	496
**Crystal size [mm]**	0.04 · 0.04 · 0.11
**2θ range [°]**	3.2 – 76.3
**Completeness [%]**	99.7
**Recorded refl.**	8403
**Independent refl.**	2200
**Goodness-of-fit F^2**	1.067
**X-Ray Source**	Cu Kα (λ = 1.54184)
**R_1 [%] /wR_2 [%] /S**	3.57/ 9.04/ 1.07

Table S13. *Distinctive energy values for the occurring HB obtained by AIM analysis via multiwfn conducted as assumed charged HB for two molecules (E_1), and complete interaction sphere of distinctive HB around one molecule (E_2), and as assumed neutral HB under the same conditions for E_1*, E_2*.*

Hydrogen Bond	E_1 [kJ mol^{-1}]	E_2 [kJ mol^{-1}]
N1-H6…O4	-58.11	-55.86
N1-H7…O4	-53.54	-50.50
N1-H8…O3	-45.58	-42.41
O1-H1…O3	-73.09	-71.59
	E_1* [kJ mol^{-1}]	E_2* [kJ mol^{-1}]
N1-H6…O4	-32.91	-31.39
N1-H7…O4	-29.84	-27.80
N1-H8…O3	-24.50	-22.37
O1-H1…O3	-42.96	-41.95

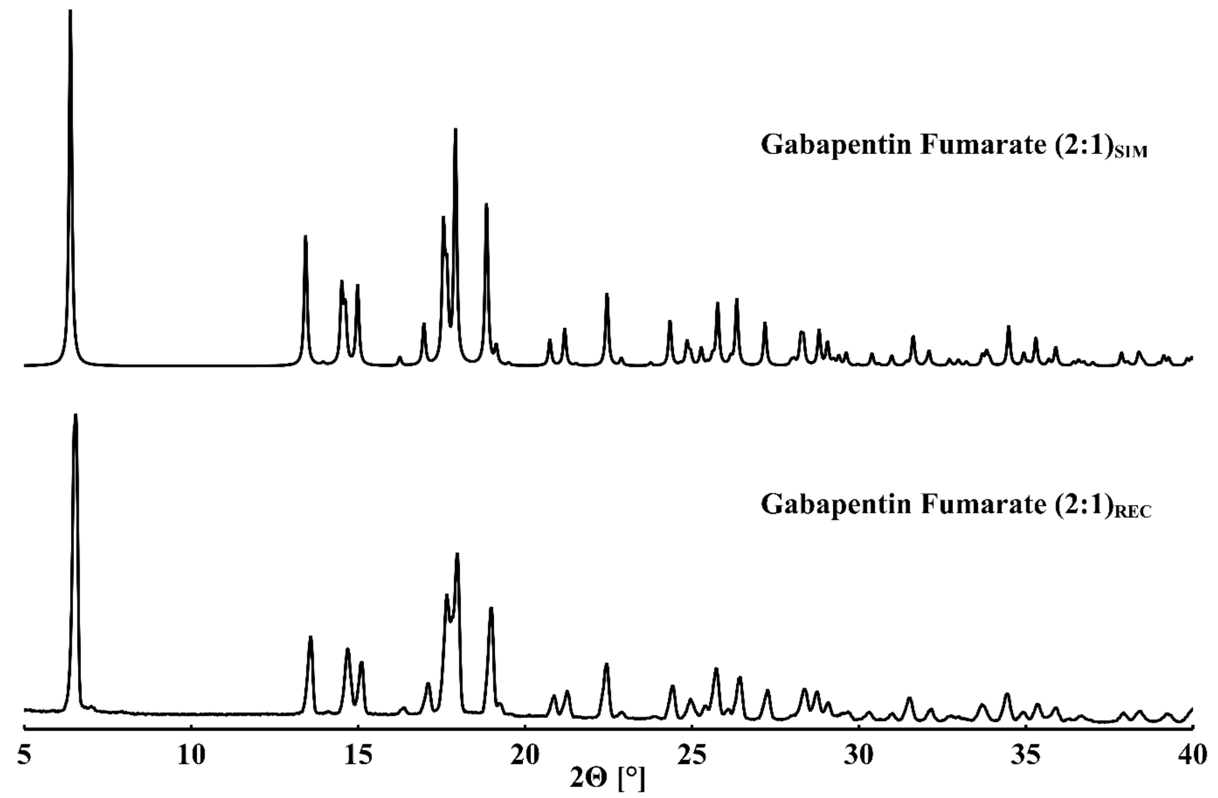

Figure S21. Powder pattern comparison of gabapentin fumarate (2:1). Simulated from single crystal data (top), recorded substance (bottom). A range between 5° 2Θ and 40° 2Θ is depicted.

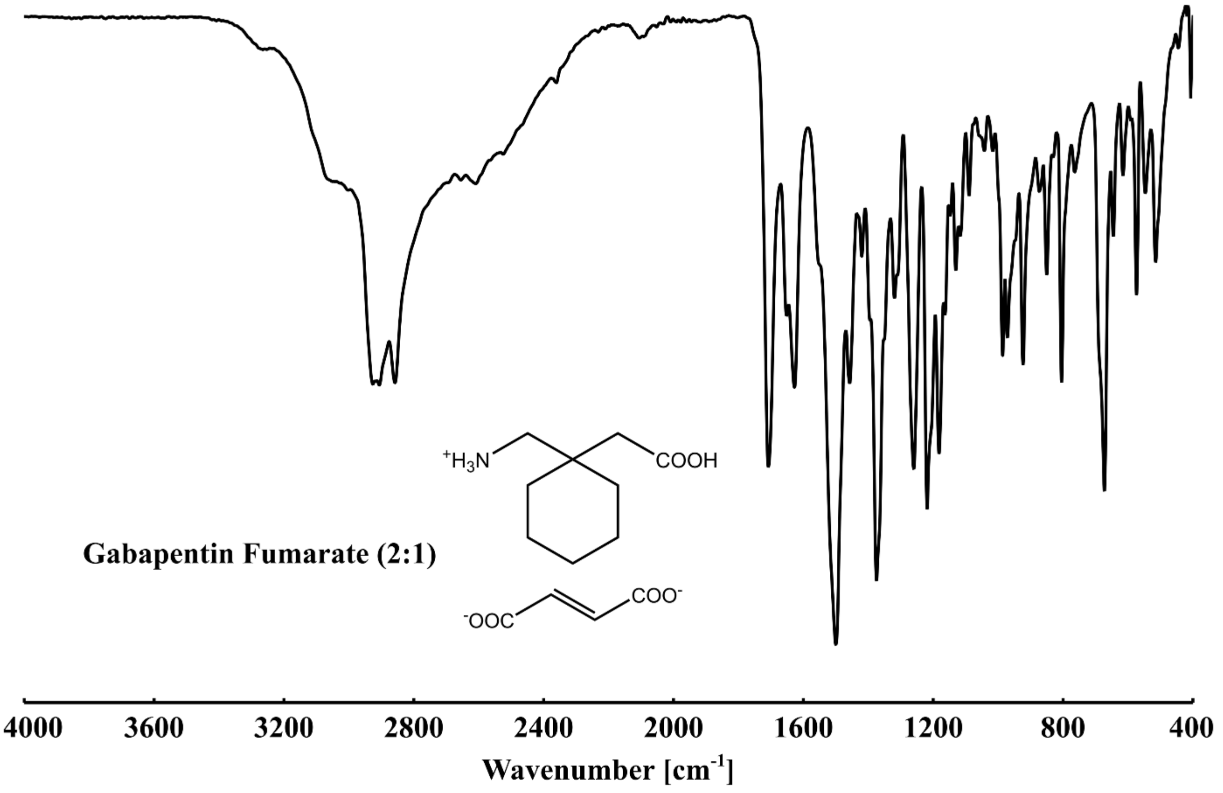

Figure S22. IR spectrum of gabapentin fumarate (2:1), shown in a range between 4000 cm^{-1} – 400 cm^{-1}. Broad ammonium hydrogen bond network and C-H stretch band between 3435 cm^{-1} and 2170 cm^{-1}, carboxyl band at 1708 cm^{-1} carboxylate band at 1627 cm^{-1}.

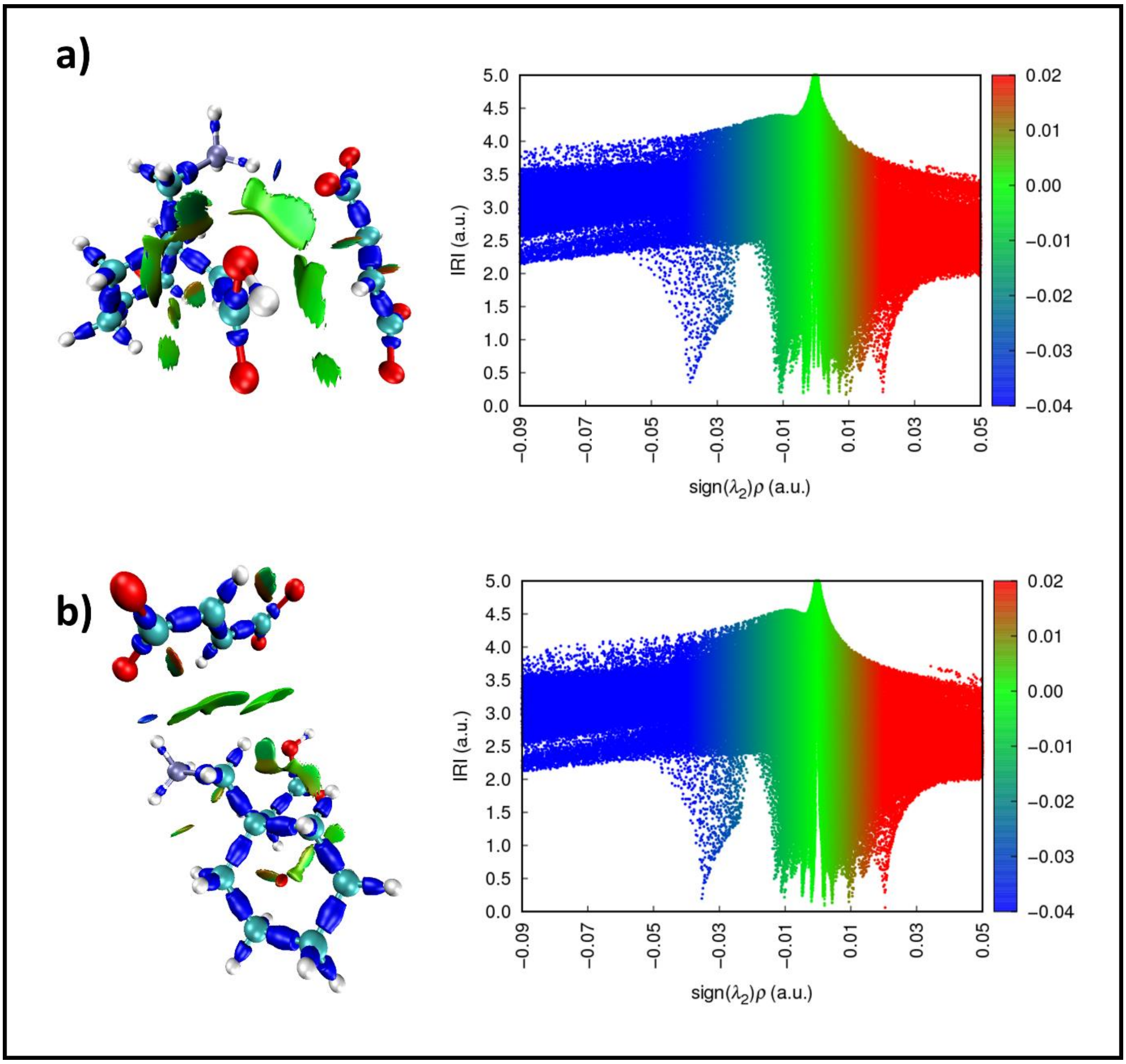

Figure S23. *Interaction Region Indicator surfaces and related scatter plots of two distinctive hydrogen bonds in gabapentin fumarate (2:1): N1-H6…O4 a), and N1-H7…O4 b). Blue regions signify strong attraction, green regions weak attraction and red regions repulsion.*

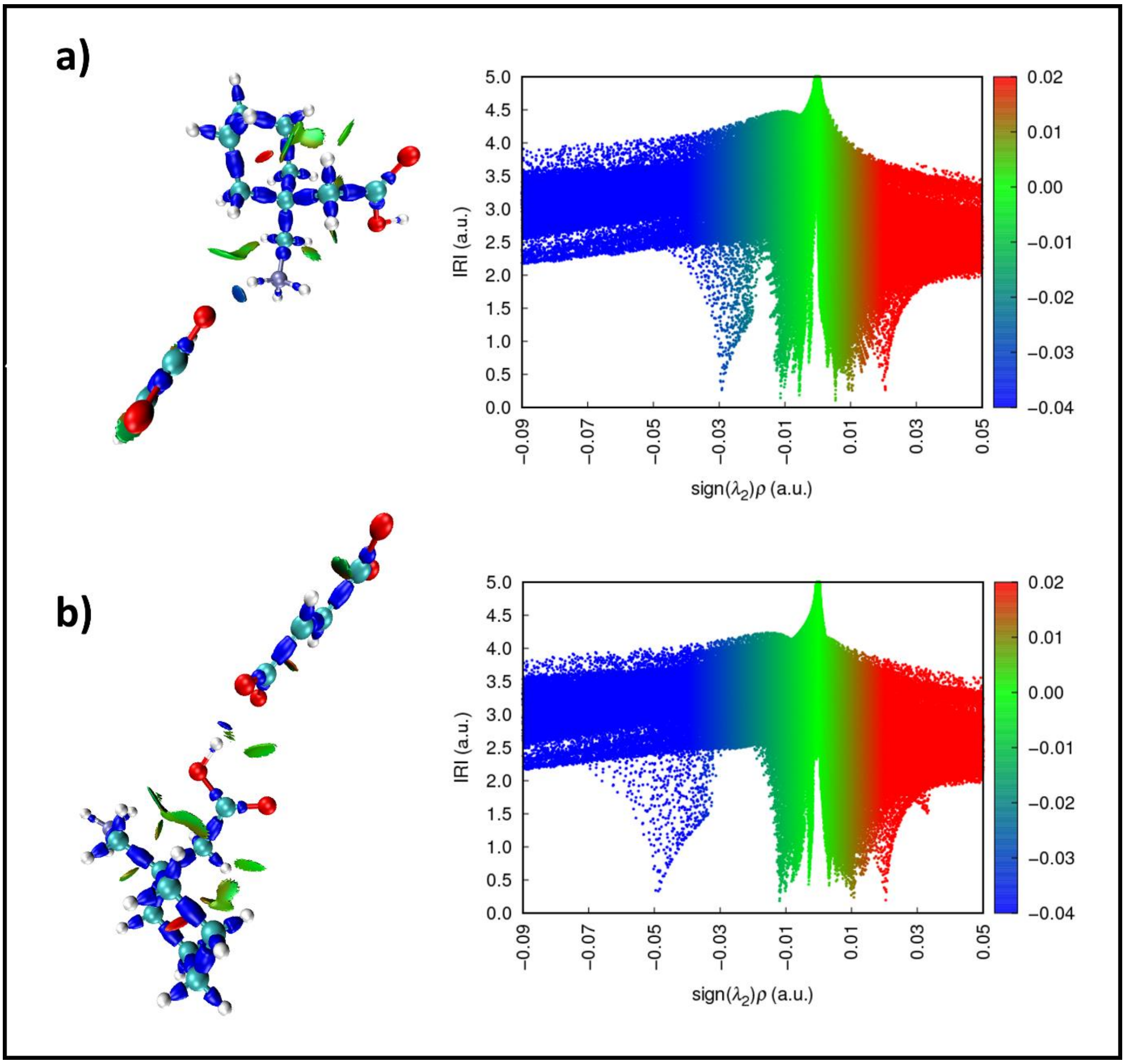

Figure S24. *Interaction Region Indicator surfaces and related scatter plots of two distinctive hydrogen bonds in gabapentin fumarate (2:1): N1-H8…O3 a), and O1-H1…O3 b). Blue regions signify strong attraction, green regions weak attraction and red regions repulsion.*

Gabapentin Fumarate (1:1) (2-3b)

Table S16. *Crystallographic data for gabapentin fumarate (1:1).*

**Parameters**	**gabapentin fumarate (1:1)**
**Formula**	$C_{13} H_{21} N O_6$
**Formula Moiety**	$C_9 H_{18} N O_2$, ($C_4 H_3 O_4$)
**M_r [g mol^{-1}]**	287.31
**Temperature [K]**	100.00(1)
**System/space group**	monoclinic, *$P2_1/n$*
**a (Å)**	15.1587(2)
**b (Å)**	7.4724(1)
**c (Å)**	25.7692(3)
**α (°)**	90
**β (°)**	102.285(1)
**γ (°)**	90
**V (Å^3)**	2852.09(6)
**Z/Z'**	8/2
**Density [g/cm^3]**	1.338
**μ [mm^{-1}]**	0. 892
**T_{min}/T_{max}**	0.835/0.957
**F (000)**	1232
**Crystal size [mm]**	0.05 · 0.15 · 0.21
**2θ range [°]**	3.1 – 77.8
**Completeness [%]**	100
**Recorded refl.**	35528
**Independent refl.**	5073
**Goodness-of-fit F^2**	1.038
**X-Ray Source**	Cu Kα (λ = 1.54184)
**R_1 [%] /wR_2 [%] /S**	2.85/ 7.59/ 1.04

Table S17. *Distinctive energy values for the occurring HB obtained by AIM analysis via multiwfn conducted as assumed charged HB for two molecules (E_1), and complete interaction sphere of distinctive HB around one molecule (E_2), and as assumed neutral HB under the same conditions for E_1*, E_2*.*

Hydrogen Bond	E_1 [kJ mol^{-1}]	E_2 [kJ mol^{-1}]
N1-H6…O2	-35.31	-35.95
N1-H7…O10	-14.97	-14.28
N1-H7…O12	-37.22	-36.26
N1-H8…O2	-17.06	-16.46
N1-H8…O12	-38.97	-37.89
N2-H24…O4	-14.74	-14.74
N2-H24…O8	-33.00	-31.73
N2-H25…O9	-41.64	-39.53
N2-H26…O6	-48.08	-46.56
O1-H1…O5	-111.08	-111.02
O3-H19…O9	-65.75	-64.82
O7-H39…O6	-76.81	-79.08
O11-H42…O10	-110.63	-110.63
	E_1* [kJ mol^{-1}]	E_2* [kJ mol^{-1}]
N1-H6…O2	-17.60	-18.03
N1-H7…O10	-3.95	-3.49
N1-H7…O12	-18.89	-18.24
N1-H8…O2	-5.35	-4.95
N1-H8…O12	-20.06	-19.34
N2-H24…O4	-3.80	-3.80
N2-H24…O8	-16.05	-15.20
N2-H25…O9	-21.85	-20.43
N2-H26…O6	-26.17	-25.16
O1-H1…O5	-68.46	-68.42
O3-H19…O9	-38.04	-37.41
O7-H39…O6	-45.46	-46.98
O11-H42…O10	-68.16	-68.16

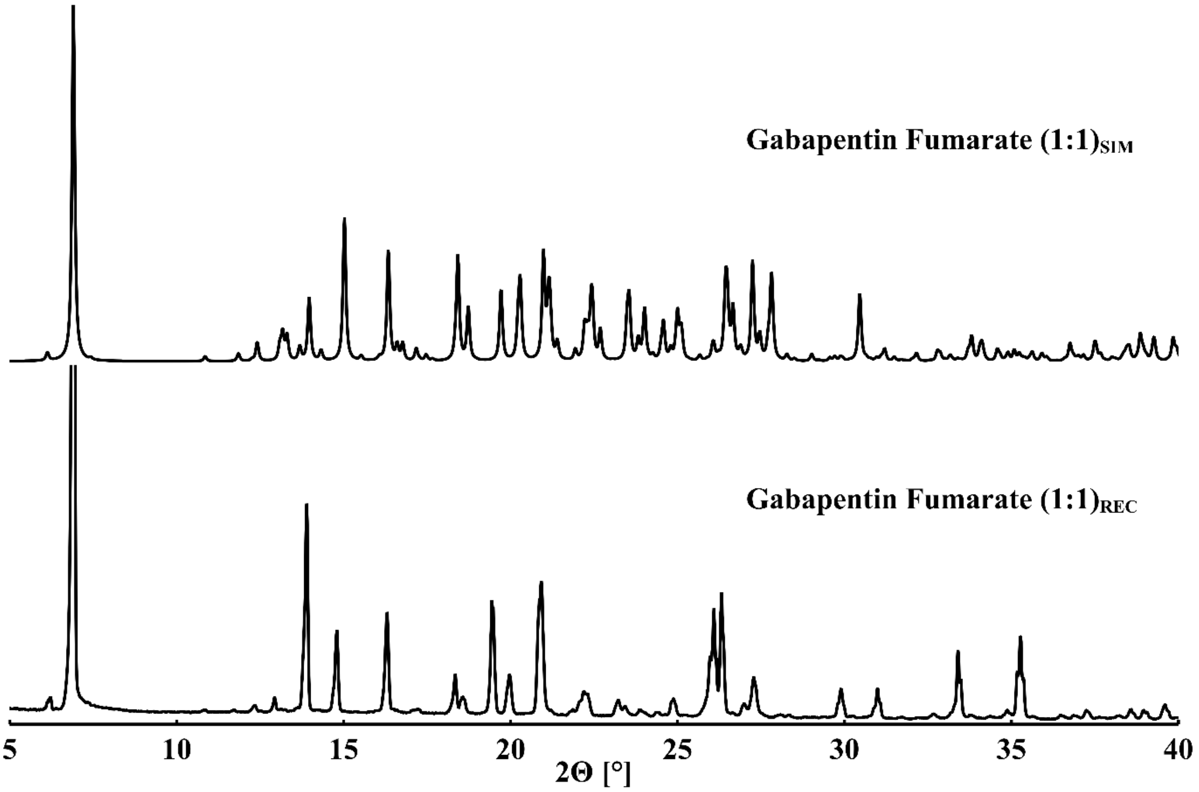

Figure S25. *Powder pattern comparison of gabapentin fumarate (1:1). Simulated from single crystal data (top), recorded substance (bottom). A range between 5° 2Θ and 40° 2Θ is depicted.*

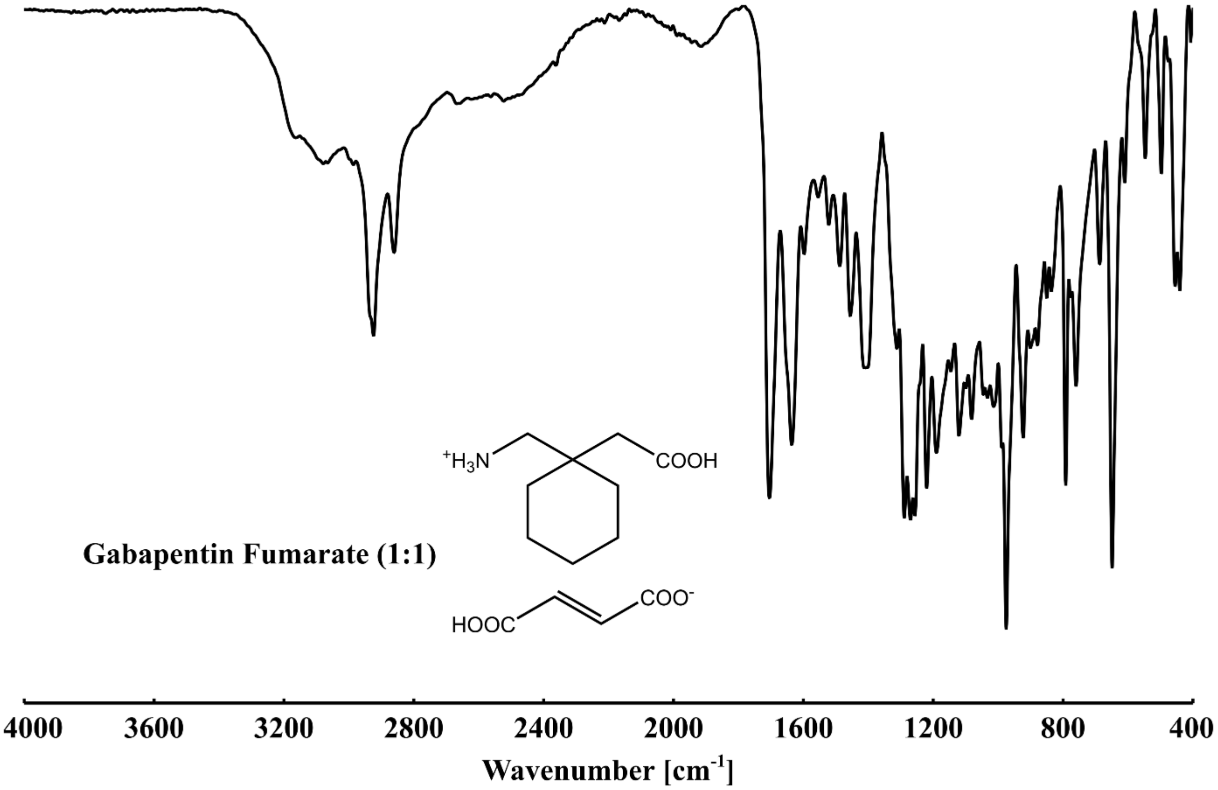

Figure S26. *IR spectrum of gabapentin fumarate (1:1), shown in a range between 4000 cm^{-1} – 400 cm^{-1}. Broad ammonium hydrogen bond network and C-H stretch band between 3400 cm^{-1} and 2195 cm^{-1}, carboxyl band at 1704 cm^{-1} carboxylate band at 1636 cm^{-1}.*

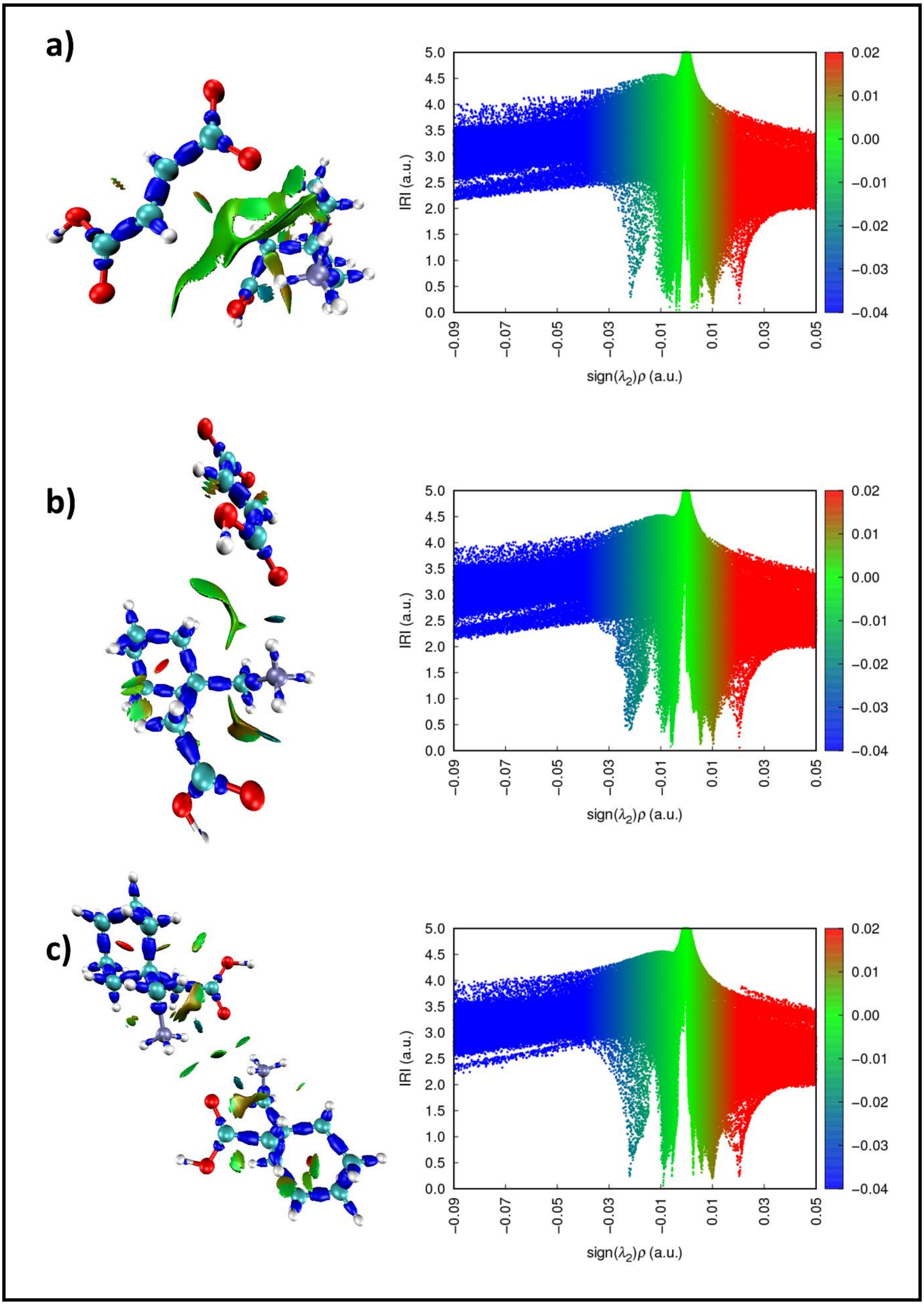

Figure S27. Interaction Region Indicator surfaces and related scatter plots of three distinctive hydrogen bonds in gabapentin fumarate (1:1): N1-H7…O10 a), N1-H7…O12 b), and N1-H8…O2 c). The intramolecular hydrogen bond N1-H6…O2 is best visible in c), but present in each depiction. Blue regions signify strong attraction, green regions weak attraction and red regions repulsion.

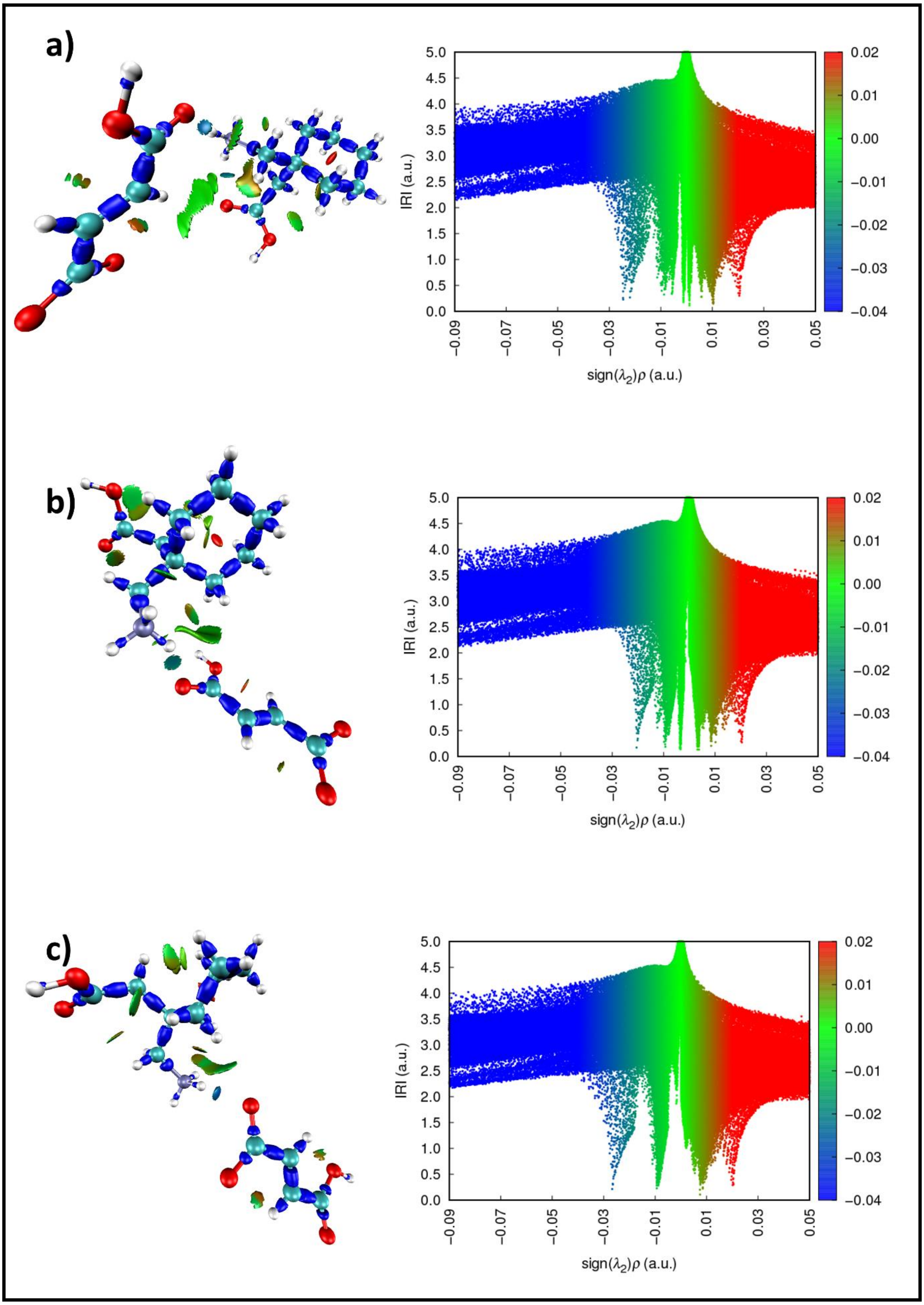

Figure S28. *Interaction Region Indicator surfaces and related scatter plots of three distinctive hydrogen bonds in gabapentin fumarate (1:1): N1-H8…O12 a), N2-H24…O8 b), and N2-H25…O9 c). The intramolecular hydrogen bond N1-H6…O2 is best visible in a). Blue regions signify strong attraction, green regions weak attraction and red regions repulsion.*

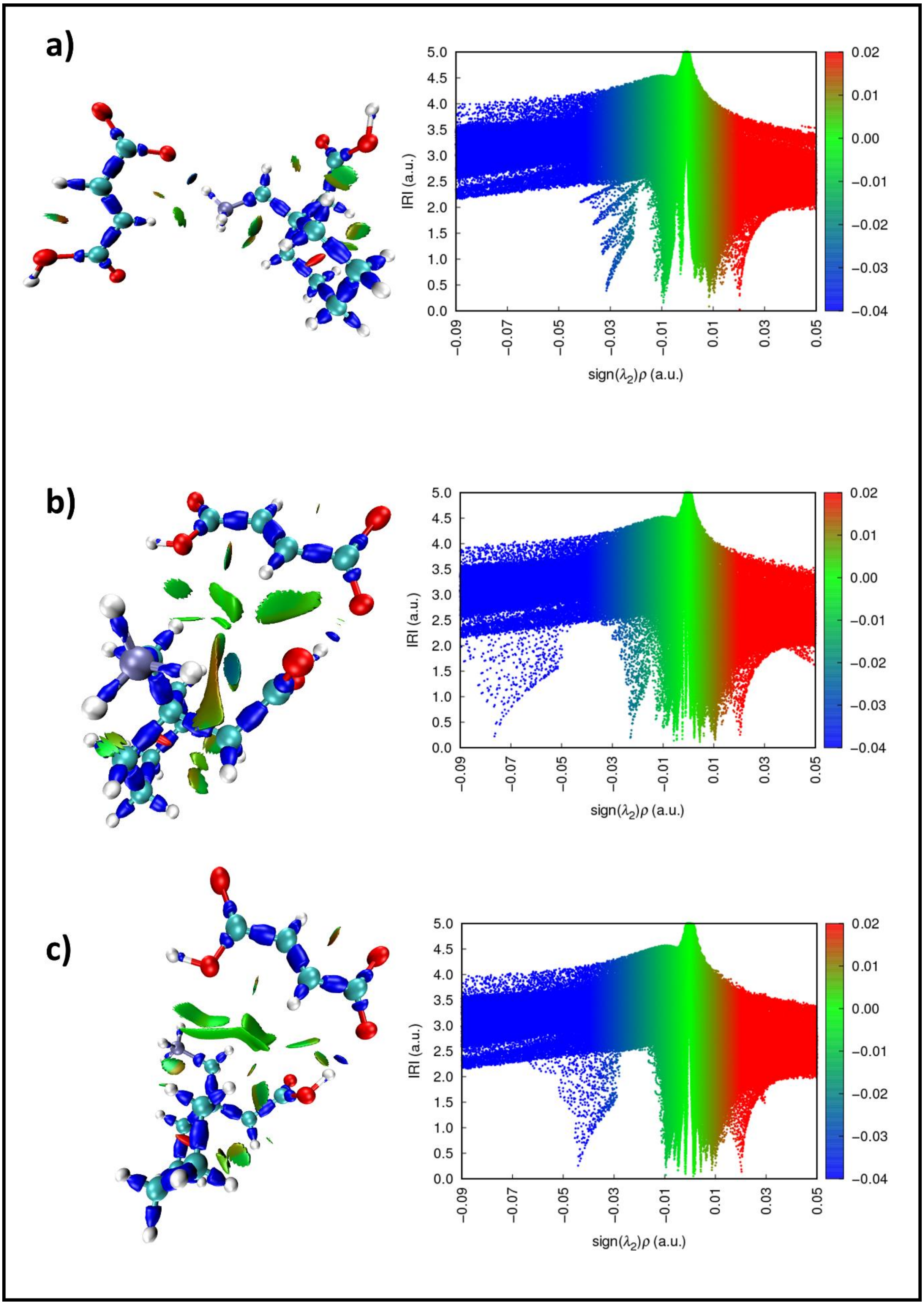

Figure S29. *Interaction Region Indicator surfaces and related scatter plots of three distinctive hydrogen bonds in gabapentin fumarate (1:1): N2-H26…O6 a), O1-H1…O5 b), and O3-H19…O9 c). The weak hydrogen bond N2-H24…O4 is visible in c). Blue regions signify strong attraction, green regions weak attraction and red regions repulsion.*

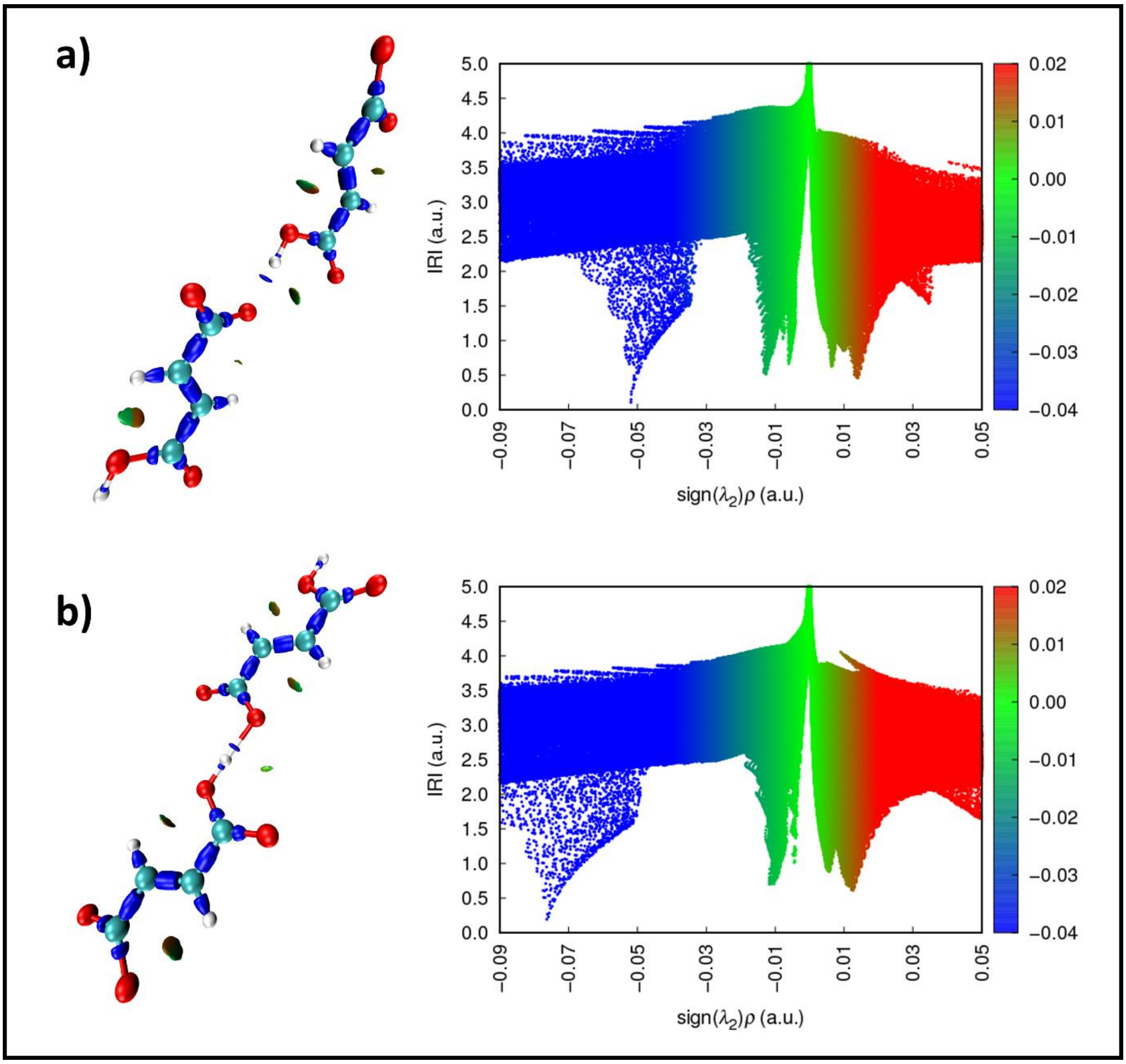

__Figure S30.__ Interaction Region Indicator surfaces and related scatter plots of two distinctive hydrogen bonds in gabapentin fumarate (1:1): O7-H39…O6 a), and O11-H42…O10 b). The hydrogen bond strength in b) crossed the threshold for weak covalent interactions. Blue regions signify strong attraction, green regions weak attraction and red regions repulsion.

GABA Succinate (2:1) (1-4a)

Table S18. *Crystallographic data for GABA succinate (2:1).*

**Parameters**	**GABA succinate (2:1)**
**Formula**	C_{12} H_{24} N_2 O_8
**Formula Moiety**	2(C_4 H_{10} N O_2), C_4 H_4 O_4
**M_r [g mol^{-1}]**	324.33
**Temperature [K]**	100(1)
**System/space group**	monoclinic, *P2₁/c*
**a (Å)**	16.2100(6)
**b (Å)**	6.3619(2)
**c (Å)**	15.9402(5)
**α (°)**	90
**β (°)**	101.211(4)
**γ (°)**	90
**V (Å^3)**	1612.49(10)
**Z/Z'**	4/1
**Density [g/cm^3]**	1.336
**μ [mm^{-1}]**	0.961
**T_{min}/T_{max}**	0.877/ 0.981
**F (000)**	696
**Crystal size [mm]**	0.02 · 0.11 · 0.14
**2θ range [°]**	2.7 – 67.0
**Completeness [%]**	99.0
**Recorded refl.**	10163
**Independent refl.**	2864
**Goodness-of-fit F^2**	1.072
**X-Ray Source**	Cu Kα (λ = 1.54184)
**R_1 [%] /wR_2 [%] /S**	3.89/ 9.35/ 1.07

Table S19. *Distinctive energy values for the occurring HB obtained by AIM analysis via multiwfn conducted as assumed charged HB for two molecules (E_1), and complete interaction sphere of distinctive HB around one molecule (E_2), and as assumed neutral HB under the same conditions for E_1*, E_2*.*

Hydrogen Bond	E_1 [kJ mol^{-1}]	E_2 [kJ mol^{-1}]
N1-H8…O5	-52.26	-49.35
N1-H9…O6	-41.14	-38.06
N1-H10…O6	-43.73	-42.08
N2-H18…O7	-49.47	-46.97
N2-H19…O8	-41.51	-39.58
N2-H20…O8	-51.29	-47.73
O1-H1…O5	-84.68	-83.81
O3-H11…O4	-64.86	-64.37
	E_1* [kJ mol^{-1}]	E_2* [kJ mol^{-1}]
N1-H8…O5	-28.98	-27.02
N1-H9…O6	-21.52	-19.44
N1-H10…O6	-23.26	-22.15
N2-H18…O7	-27.11	-25.43
N2-H19…O8	-21.76	-20.47
N2-H20…O8	-28.32	-25.94
O1-H1…O5	-50.74	-50.16
O3-H11…O4	-37.44	-37.11

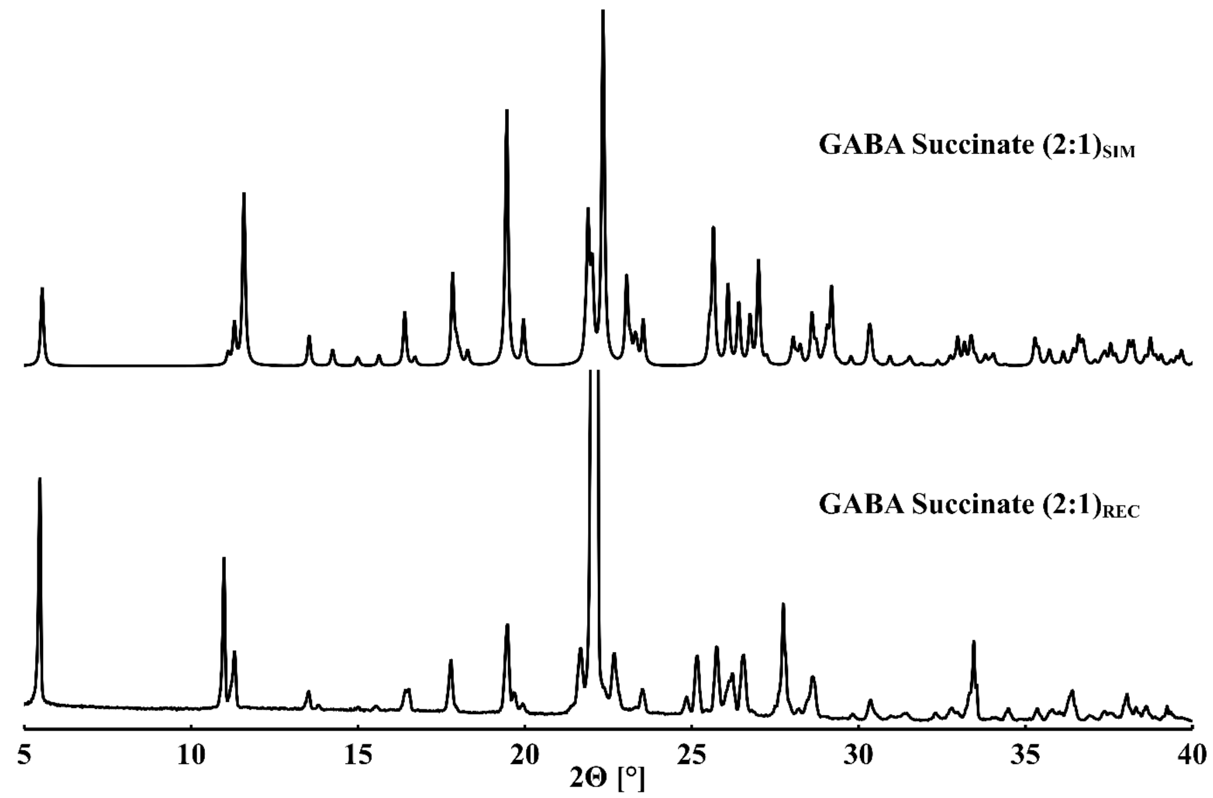

Figure S31. *Powder pattern comparison of GABA succinate (2:1). Simulated from single crystal data (top), recorded substance (bottom). A range between 5° 2Θ and 40° 2Θ is depicted.*

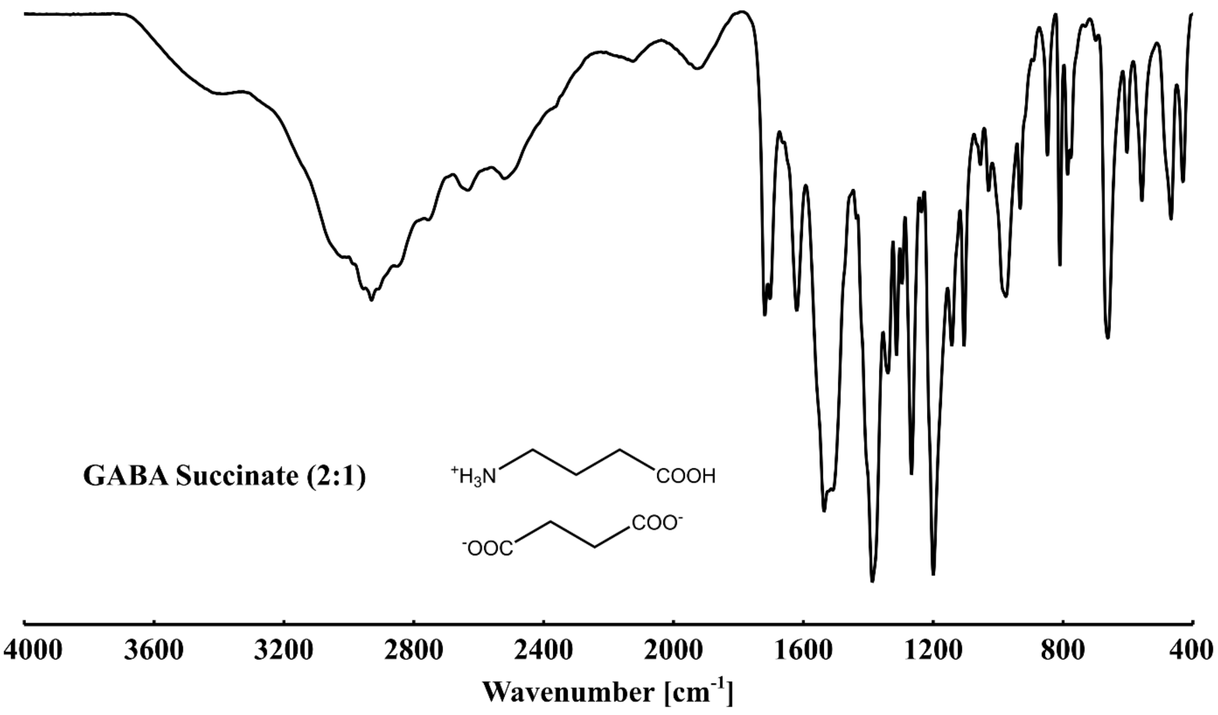

Figure S32. *IR spectrum of GABA succinate (2:1), shown in a range between 4000 cm^{-1} – 400 cm^{-1}. Broad ammonium hydrogen bond network and C-H stretch band between 3700 cm^{-1} and 2240 cm^{-1}, carboxyl band at 1718 cm^{-1} carboxylate band at 1620 cm^{-1}.*

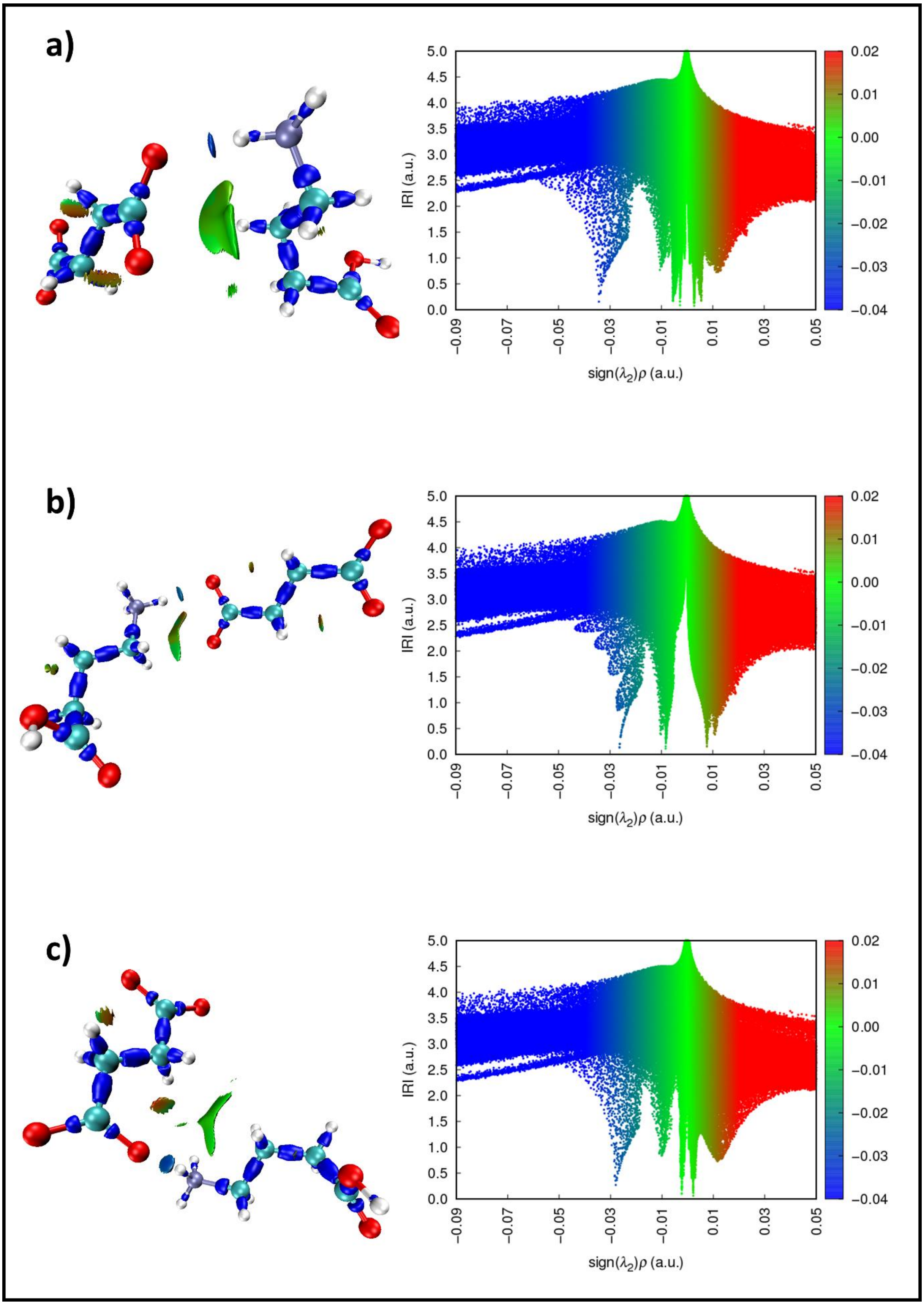

Figure S33. *Interaction Region Indicator surfaces and related scatter plots of three distinctive hydrogen bonds in GABA succinate (2:1): N1-H8…O5 a), N1-H9…O6 b), and N1-H10…O6 c). Blue regions signify strong attraction, green regions weak attraction and red regions repulsion.*

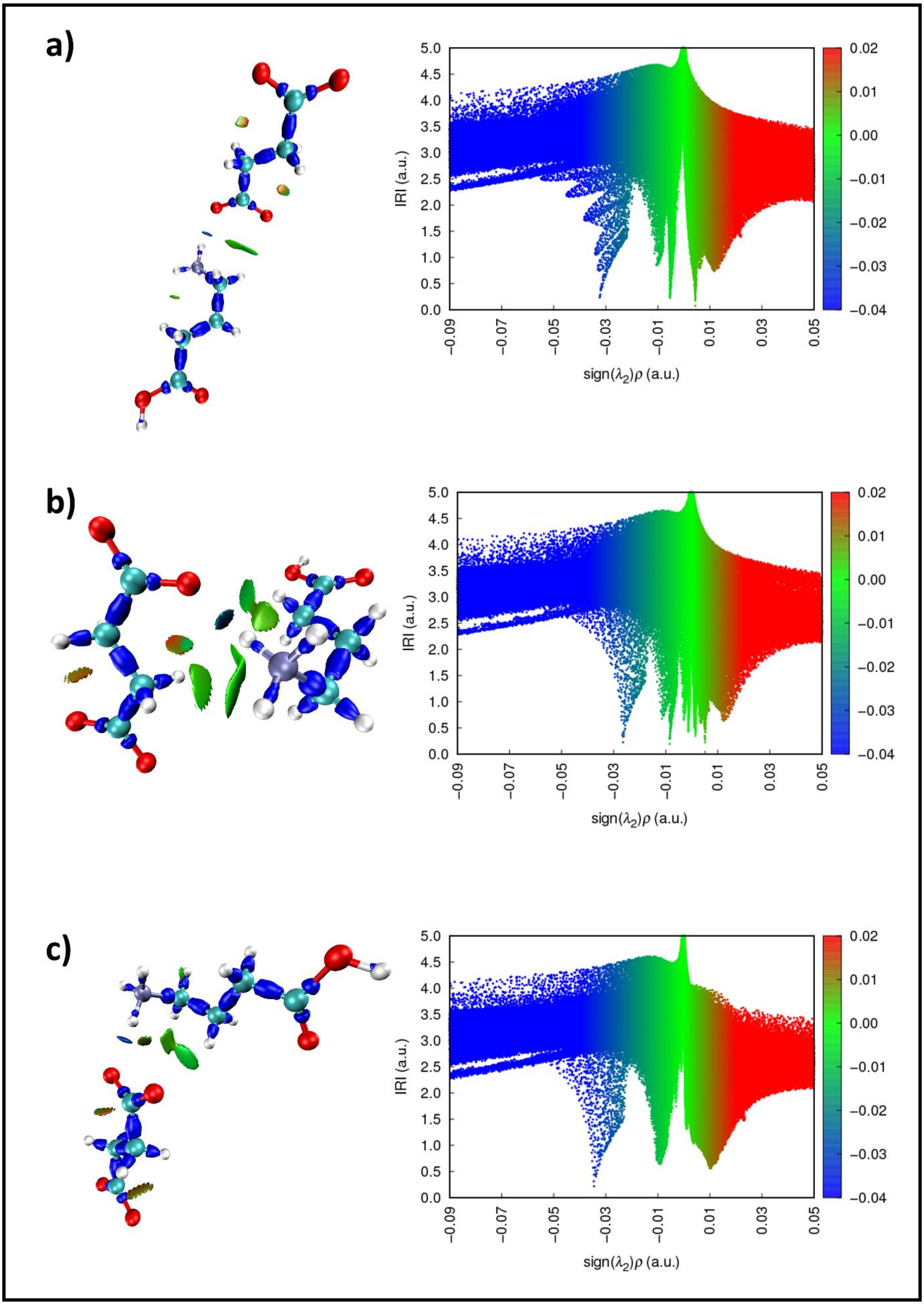

Figure S34. *Interaction Region Indicator surfaces and related scatter plots of three distinctive hydrogen bonds in GABA succinate (2:1): N2-H18…O7 a), N2-H19…O8 b), and N2-H20…O8 c). Blue regions signify strong attraction, green regions weak attraction and red regions repulsion.*

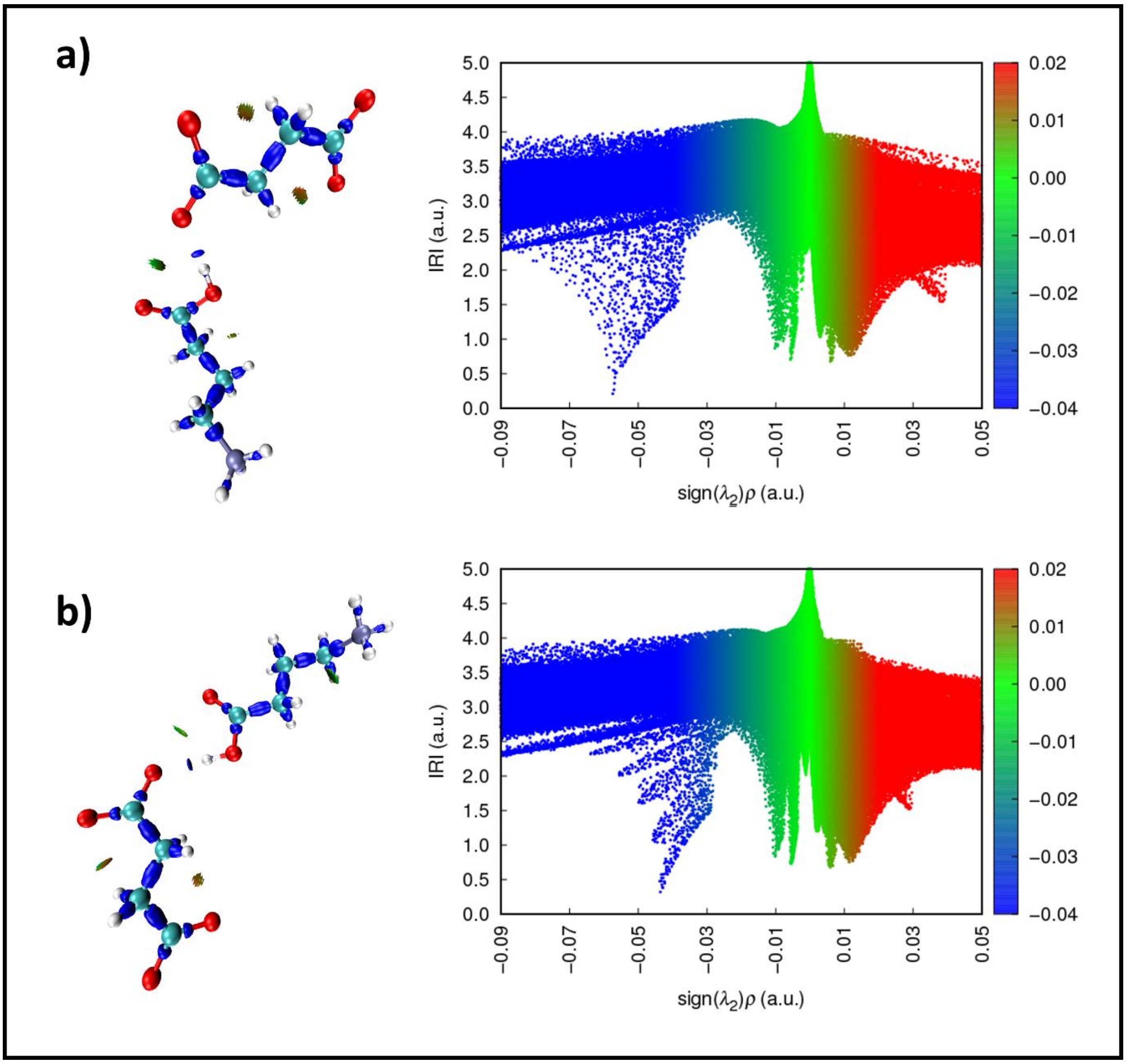

Figure S35. *Interaction Region Indicator surfaces and related scatter plots of two distinctive hydrogen bonds in GABA succinate (2:1): O1-H1…O5 a), and O3-H11…O7 b). Blue regions signify strong attraction, green regions weak attraction and red regions repulsion.*

GABA Succinate (1:1) (1-4b)

Table S20. *Crystallographic data GABA succinate (1:1).*

**Parameters**	**GABA succinate (1:1)**
**Formula**	$C_8 H_{15} N O_6$
**Formula Moiety**	$C_4 H_{10} N O_2$, $C_4 H_5 O_4$
**M_r [g mol^{-1}]**	221.21
**Temperature [K]**	99.8(3)
**System/space group**	monoclinic, $P2_1$
**a (Å)**	5.1129(1)
**b (Å)**	13.3267(2)
**c (Å)**	7.1747(1)
**α (°)**	90
**β (°)**	95.396(1)
**γ (°)**	90
**V (Å^3)**	486.704(14)
**Z/Z'**	2/1
**Density [g/cm^3]**	1.509
**μ [mm^{-1}]**	1.120
**T_{min}/T_{max}**	0.558/0.824
**F (000)**	236
**Crystal size [mm]**	0.18 · 0.22 · 0.59
**2θ range [°]**	3.3 – 77.5
**Completeness [%]**	99.8
**Recorded refl.**	4662
**Independent refl.**	1730
**Goodness-of-fit F^2**	1.108
**X-Ray Source**	Cu Kα (λ = 1.54184)
**Flack x**	0.07(7)
**R_1 [%] /wR_2 [%] /S**	2.32/ 5.93/ 1.11

Table S14. *Distinctive energy values for the occurring HB obtained by AIM analysis via multiwfn conducted as assumed charged HB for two molecules (E_1), and complete interaction sphere of distinctive HB around one molecule (E_2), and as assumed neutral HB under the same conditions for E_1*, E_2*.*

Hydrogen Bond	E_1 [kJ mol^{-1}]	E_2 [kJ mol^{-1}]
N1-H8…O2	-38.57	-38.24
N1-H9…O2	-33.56	-33.86
N1-H10…O6	-30.40	-30.26
O1-H1…O3	-104.67	-104.44
O5-H11…O4	-52.74	-53.37
	E_1* [kJ mol^{-1}]	E_2* [kJ mol^{-1}]
N1-H8…O2	-19.79	-19.57
N1-H9…O2	-16.43	-16.63
N1-H10…O6	-14.30	-14.21
O1-H1…O3	-64.16	-64.01
O5-H11…O4	-29.30	-29.73

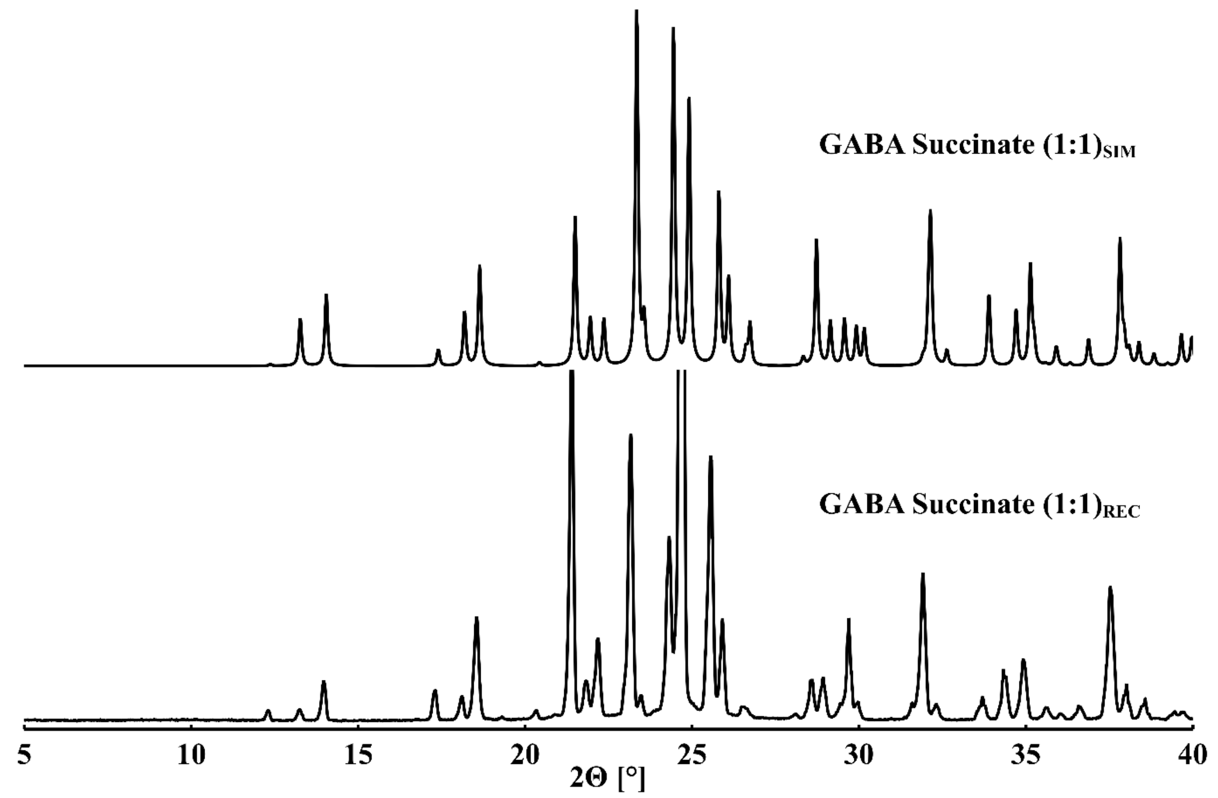

Figure S36. *Powder pattern comparison of GABA succinate (1:1). Simulated from single crystal data (top), recorded substance (bottom). A range between 5° 2Θ and 40° 2Θ is depicted.*

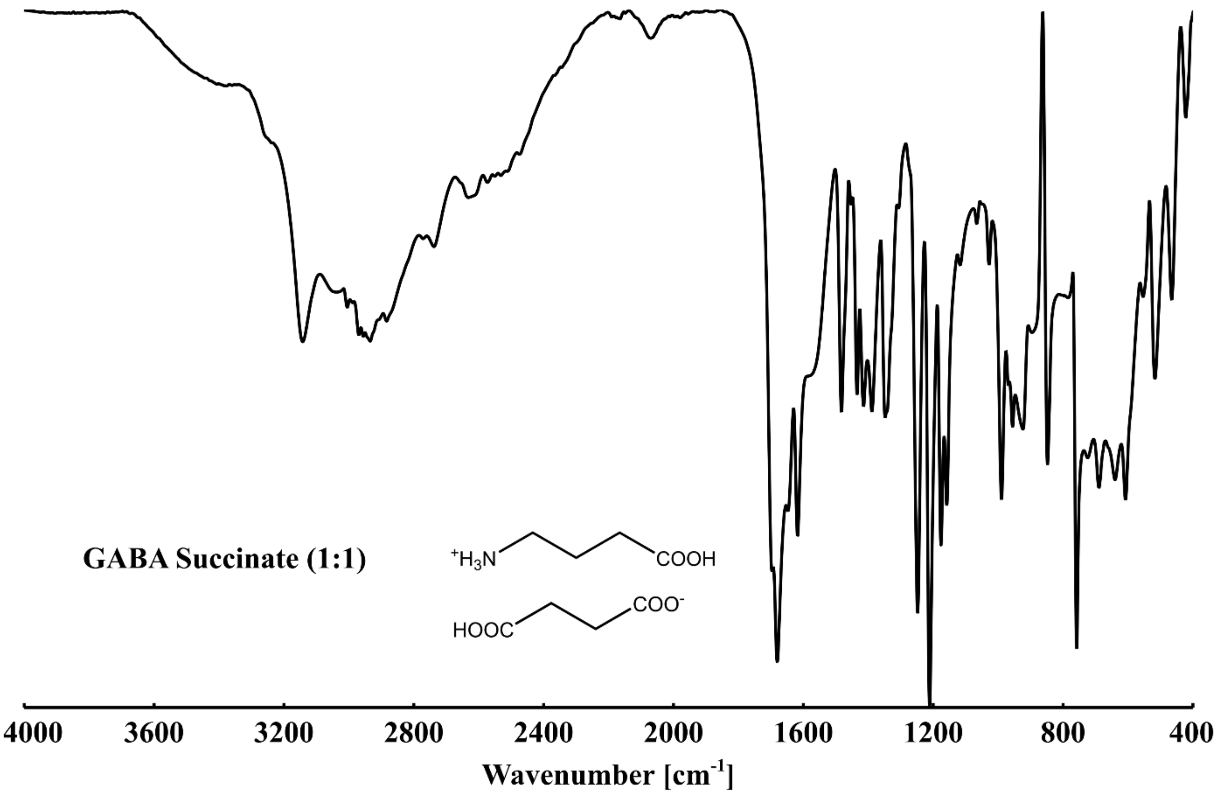

Figure S37. *IR spectrum of GABA succinate (1:1), shown in a range between 4000 cm^{-1} – 400 cm^{-1}. Broad ammonium hydrogen bond network and C-H stretch band between 3690 cm^{-1} and 2210 cm^{-1}, carboxyl band at 1680 cm^{-1} carboxylate band at 1618 cm^{-1}.*

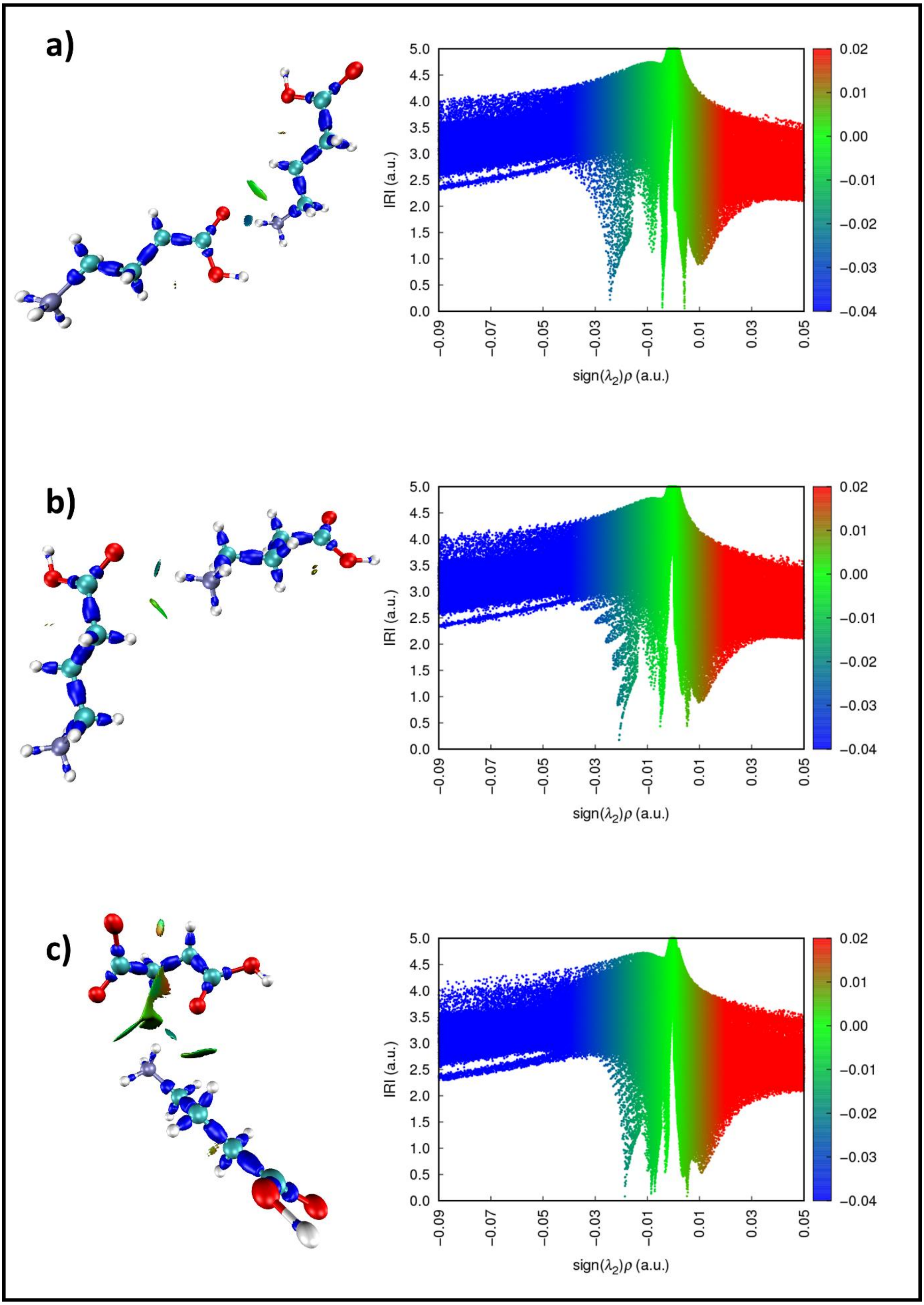

Figure S38. *Interaction Region Indicator surfaces and related scatter plots of three distinctive hydrogen bonds in GABA succinate (1:1): N1-H8…O2 a), N1-H9…O2 b), and N1-H10…O6 c). Blue regions signify strong attraction, green regions weak attraction and red regions repulsion.*

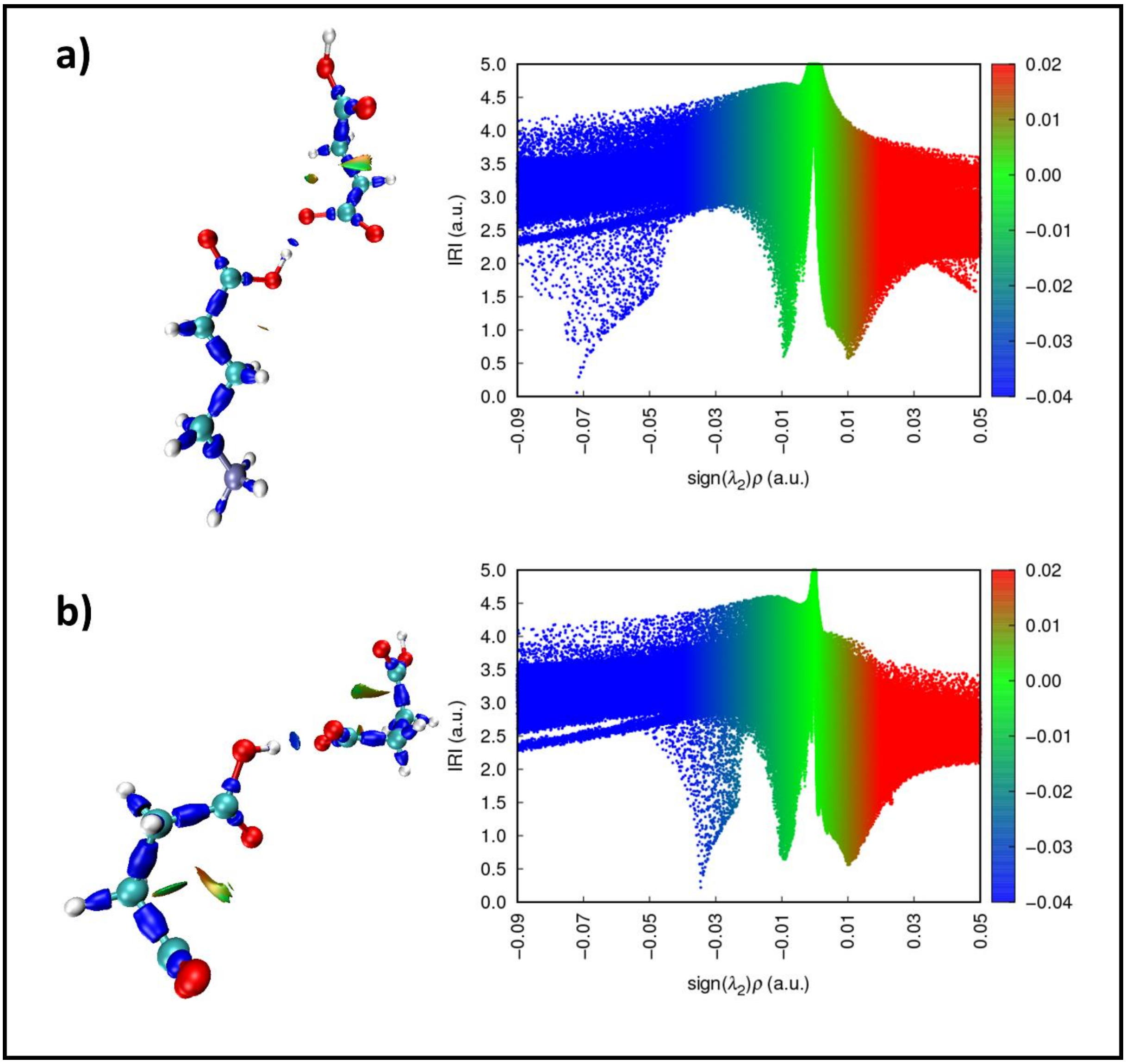

Figure S39. *Interaction Region Indicator surfaces and related scatter plots of two distinctive hydrogen bonds in GABA succinate (1:1): O1-H1…O3 a), and O5-H11…O4 b). Blue regions signify strong attraction, green regions weak attraction and red regions repulsion.*

Gabapentin:Succinic acid (2:1) (2-4)

Table S22. *Crystallographic data for gabapentin:succinic acid (2:1).*

**Parameters**	**gabapentin:succinic acid (2:1)**
**Formula**	$C_{11} H_{20} N O_8$
**Formula Moiety**	$C_9 H_{17} N O_2$, 0.5($C_4 H_6 O_4$)
**M_r [g mol^{-1}]**	230.28
**Temperature [K]**	100.01(1)
**System/space group**	monoclinic, *P2₁/c*
**a (Å)**	18.4497(5)
**b (Å)**	6.4446(1)
**c (Å)**	10.3289(3)
**α (°)**	90
**β (°)**	103.111(3)
**γ (°)**	90
**V (Å^3)**	1196.10(5)
**Z/Z'**	4/1
**Density [g/cm^3]**	1.279
**μ [mm^{-1}]**	0.800
**T_{min}/T_{max}**	1.000/1.000
**F (000)**	500
**Crystal size [mm]**	0.06 · 0.10 · 0.22
**2θ range [°]**	2.4 – 77.8
**Completeness [%]**	99.7
**Recorded refl.**	12538
**Independent refl.**	2134
**Goodness-of-fit F^2**	1.077
**X-Ray Source**	Cu Kα (λ = 1.54184)
**R_1 [%] /wR_2 [%] /S**	5.32/ 13.55/ 1.08

Table S23. *Distinctive energy values for the occurring HB obtained by AIM analysis via multiwfn conducted as assumed charged HB for two molecules (E_1), and complete interaction sphere of distinctive HB around one molecule (E_2), and as assumed neutral HB under the same conditions for E_1*, E_2*.*

Hydrogen Bond	E_1 [kJ mol^{-1}]	E_2 [kJ mol^{-1}]
N1-H5…O1	-36.47	-36.02
N1-H5…O3	-16.71	-16.67
N1-H6…O4	-38.62	-38.37
N1-H7…O2	-56.03	-55.59
O3-H18…O1	-111.50	-112.78
	E_1* [kJ mol^{-1}]	E_2* [kJ mol^{-1}]
N1-H5…O1	-18.38	-18.08
N1-H5…O3	-5.12	-5.09
N1-H6…O4	-19.82	-19.66
N1-H7…O2	-31.51	-31.22
O3-H18…O1	-68.74	-69.60

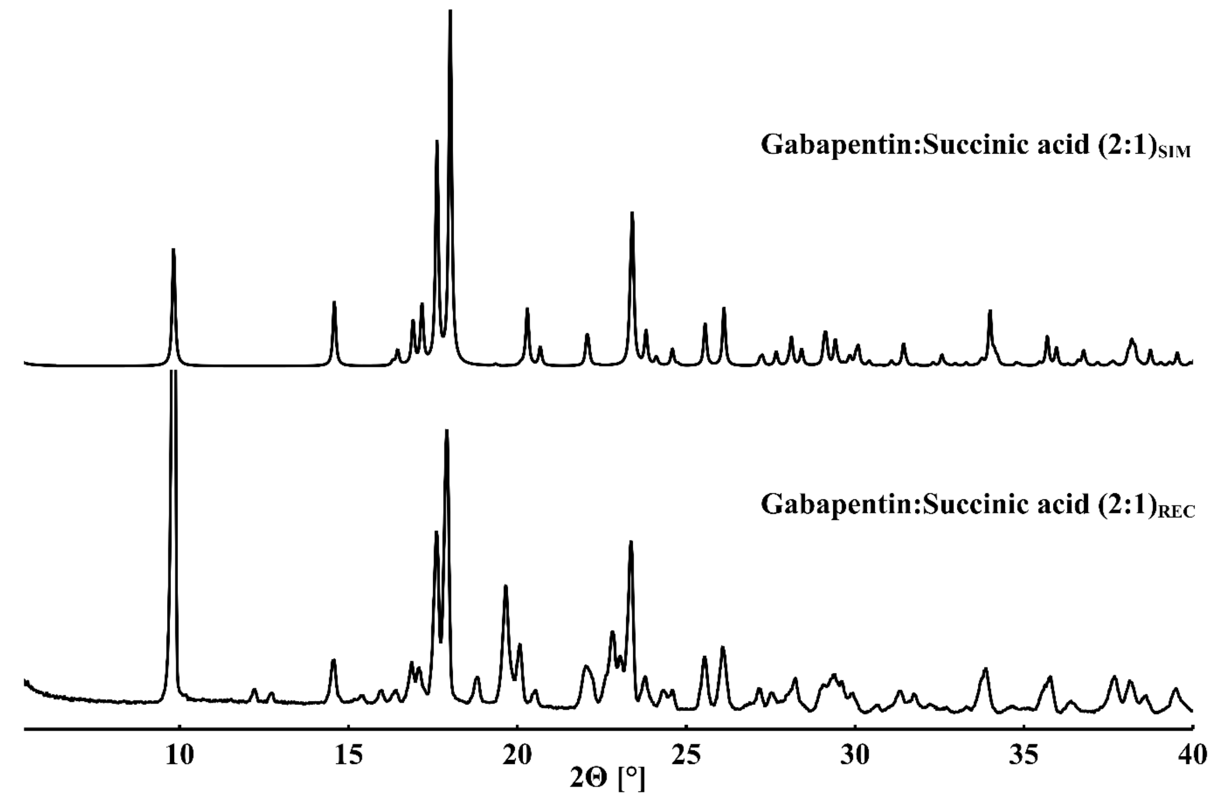

Figure S40. *Powder pattern comparison of gabapentin:succinic acid (2:1). Simulated from single crystal data (top), recorded substance (bottom). A range between 5° 2Θ and 40° 2Θ is depicted.*

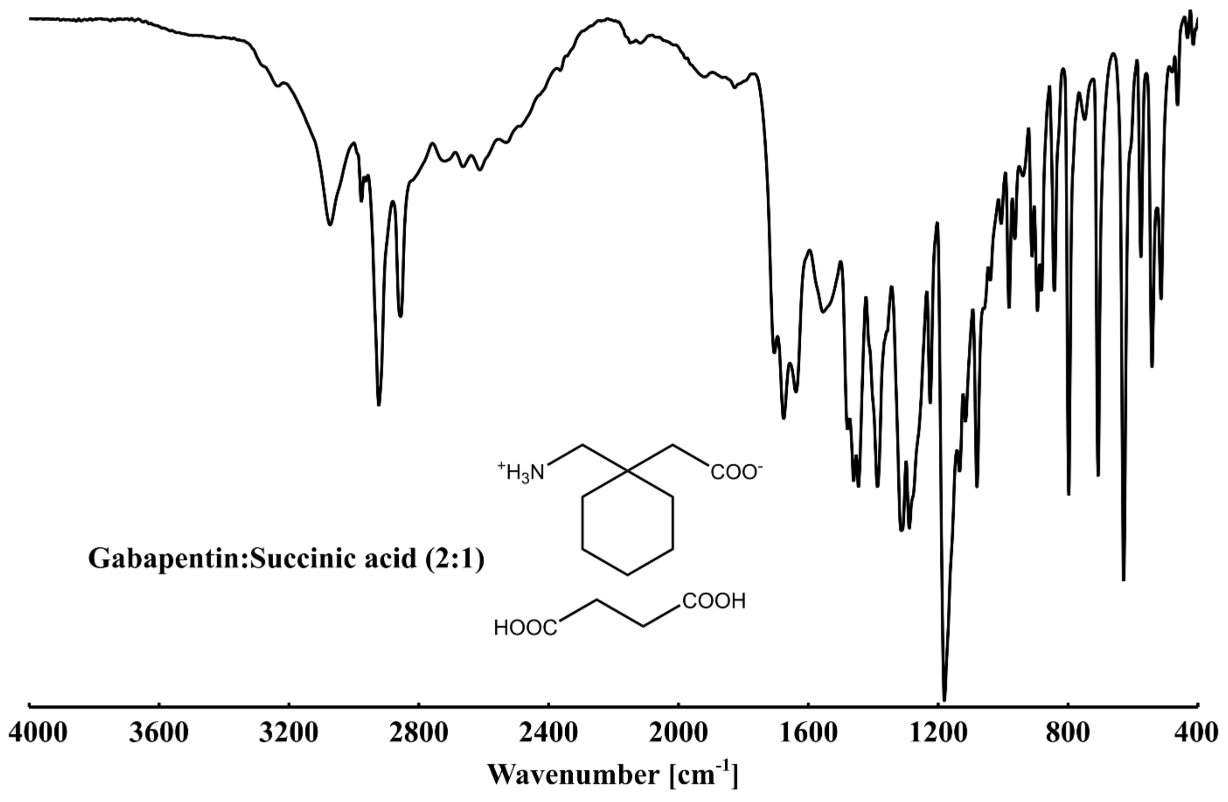

Figure S41. *IR spectrum of gabapentin:succinic acid (2:1), shown in a range between 4000 cm^{-1} – 400 cm^{-1}. Broad ammonium hydrogen bond network and C-H stretch band between 3660 cm^{-1} and 2225 cm^{-1}, carboxyl band at 1704 cm^{-1} carboxylate band at 1676 cm^{-1}.*

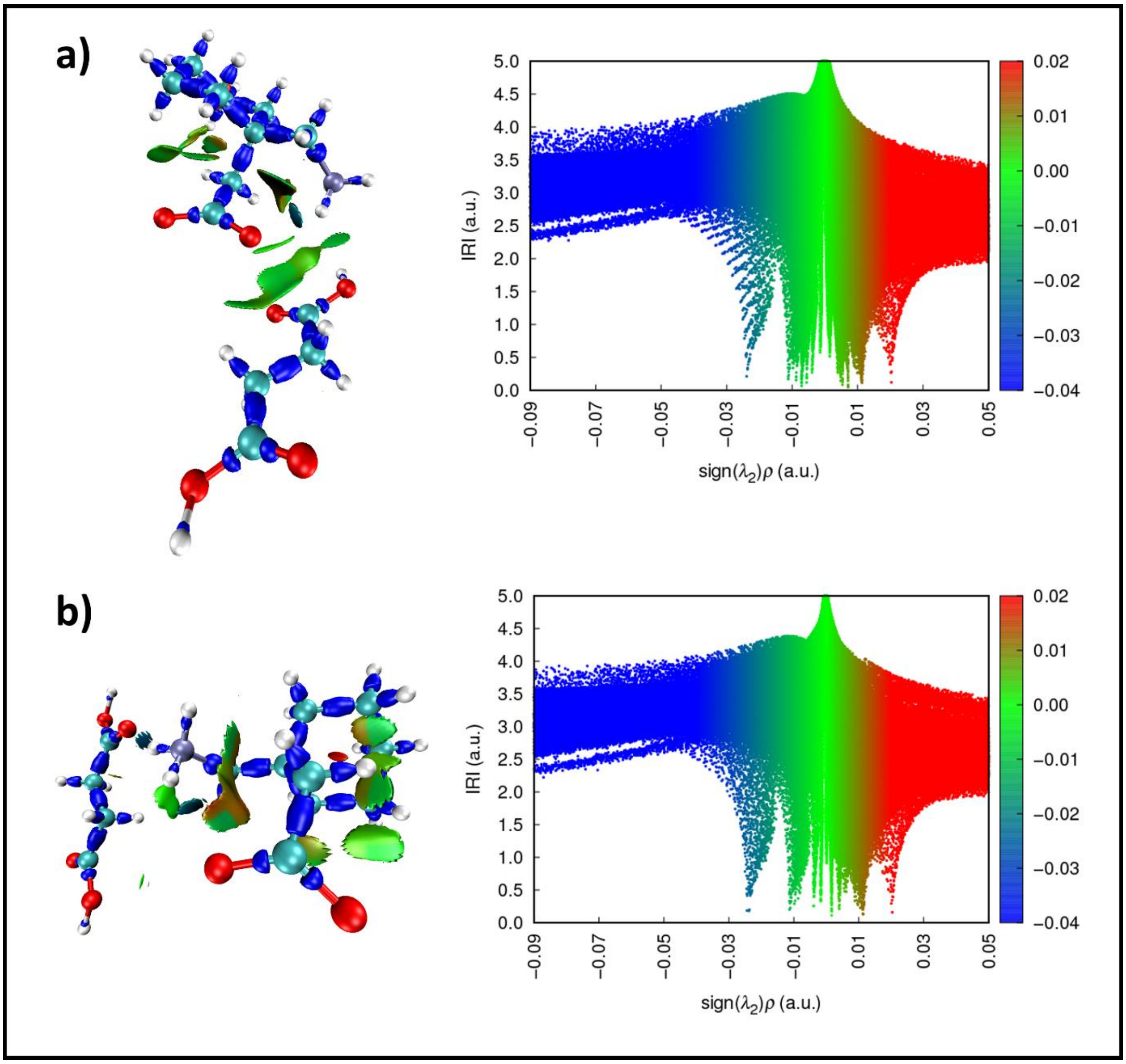

Figure S42. *Interaction Region Indicator surfaces and related scatter plots of two distinctive hydrogen bonds in gabapentin:succinic acid (2:1): N1-H5…O3 a), and N1-H6…O4 b). The intramolecular hydrogen bond N1-H5…O1 is best visible in a), but present in both. Blue regions signify strong attraction, green regions weak attraction and red regions repulsion.*

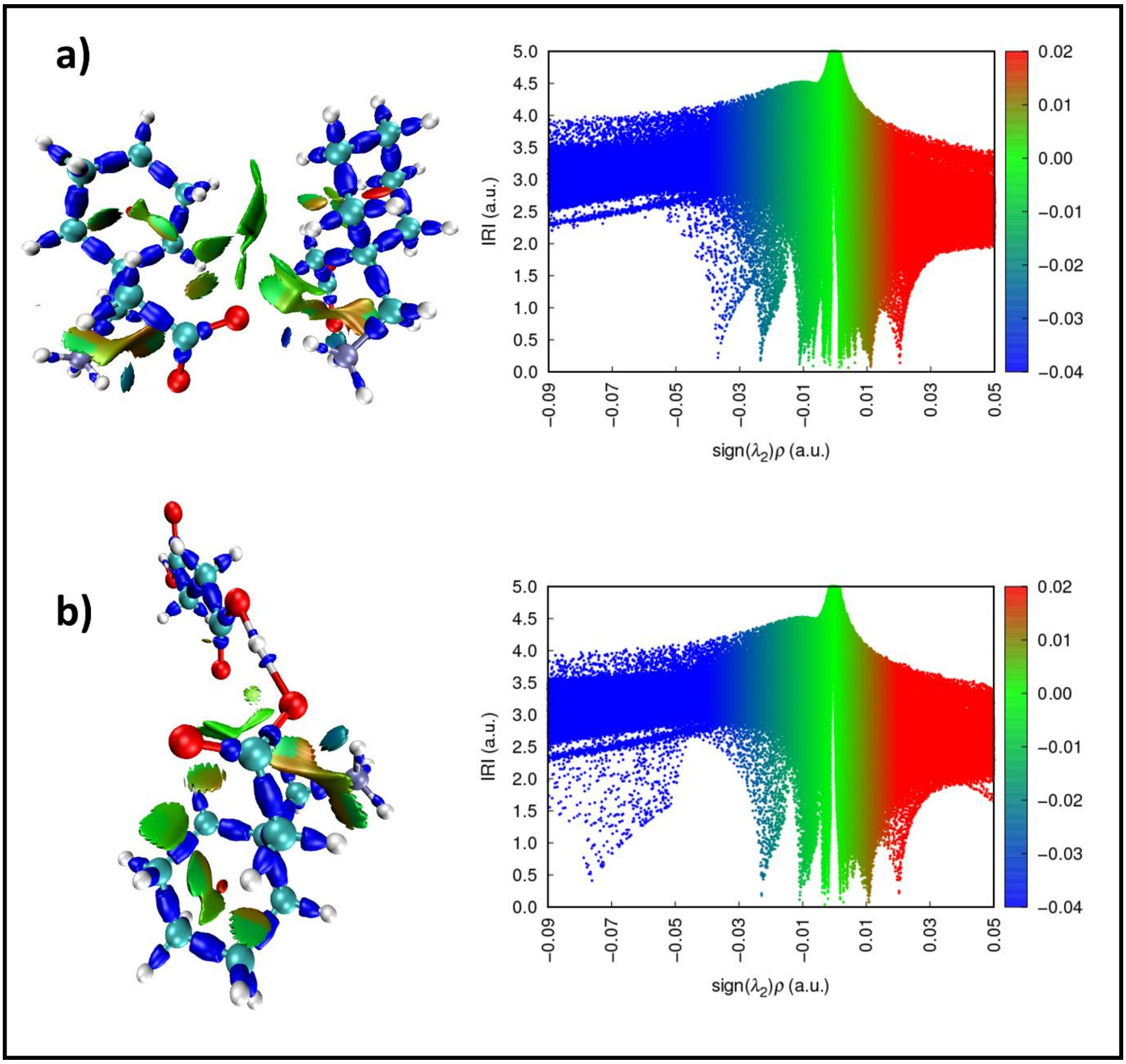

__Figure S43.__ Interaction Region Indicator surfaces and related scatter plots of two distinctive hydrogen bonds in gabapentin:succinic acid (2:1): N1-H7…O2 a), and O3-H18…O1 b). The intramolecular hydrogen bond N1-H5…O1 is best visible in a), but present in both. The hydrogen bond strength in b) crossed the threshold for weak covalent interactions. Blue regions signify strong attraction, green regions weak attraction and red regions repulsion.

Comparison of 1 and 2 multicomponent systems

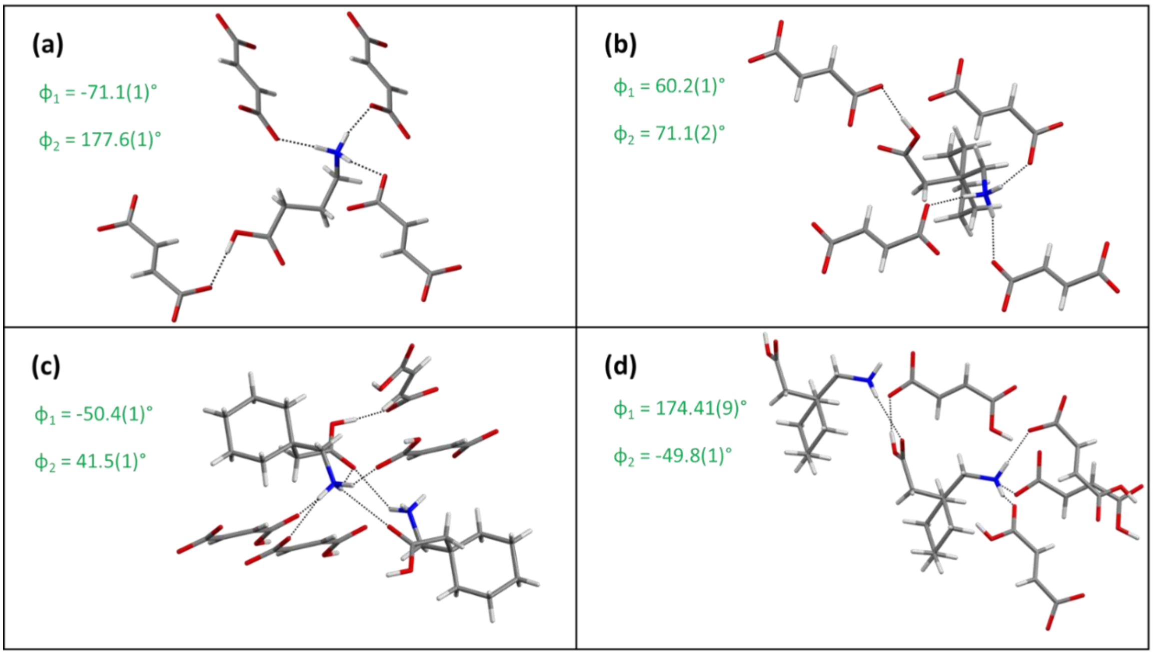

Figure S44. *Torsion angles and selected hydrogen bonds for (a)* ***1-3****, (b)* ***2-3a****, (c) first independent gabapentin molecule in* ***2-3b*** *and (d) second independent gabapentin molecule in* ***2-3b.*** *HBs are depicted as black dotted lines, torsion angles are only depicted as values for better visibility, oxygen atoms in red, nitrogen atoms in blue, carbon atoms in grey and hydrogen atoms in white.*

Table S24*. Overview on the strong, distinctive hydrogen bonds in the fumarates of GABA and gabapentin. The proton acceptor distance, the donor acceptor distance, the bond angle and the binding energy calculated for charged HBs are shown.*

Compound	Interaction	H…A [Å]	D…A [Å]	D - H…A [°]	Ebond [kJ mol^{-1}]
**1-3**	O1-H1…O3	1.72(2)	2.606(7)	179(2)	-65.80
	N1-H9…O4	1.91(0)	2.785(1)	164(3)	-49.63
	N1-H8…O3	1.89(9)	2.785(9)	163(6)	-48.84
	N1-H10…O4	1.94(2)	2.849(2)	166(1)	-44.02
**2-3a**	O1-H1…O3	1.68(3)	2.644(3)	174(2)	-73.09
	N1-H6…O4	1.81(2)	2.761(0)	174(6)	-58.11
	N1-H7…O4	1.836(16)	2.746(4)	162(2)	-53.54
	N1-H8…O3	1.935(18)	2.807(7)	151(7)	-45.58
**2-3b**	O1-H1…O5	1.51(2)	2.507(0)	175(2)	-111.08
	O11-H42…O10	1.50(2)	2.525(5)	178(3)	-110.63
	O7-H39…O6	1.65(2)	2.559(0)	170(5)	-76.81
	O3-H19…O9	1.73(2)	2.644(7)	172(9)	-65.75
	N2-H26…O6	1.875(17)	2.816(6)	178(1)	-48.08
	N2-H25…O9	1.948(15)	2.838(7)	163(0)	-41.64
	N1-H8…O12	1.973(16)	2.813(2)	148(5)	-38.97
	N1-H6…O2	2.043(15)	2.806(0)	141(0)	-35.27
	N1-H7…O12	2.005(15)	2.800(1)	144(6)	-37.23
	N2-H24…O8	2.032(16)	2.798(1)	142(0)	-33.00
	N1-H8…O2	2.486(16)	2.933(8)	109(5)	-17.06
	N1-H7…O10	2.534(17)	3.166(6)	126(8)	-14.97
	N2-H24…O4	2.597(15)	3.160(8)	130(8)	-14.74

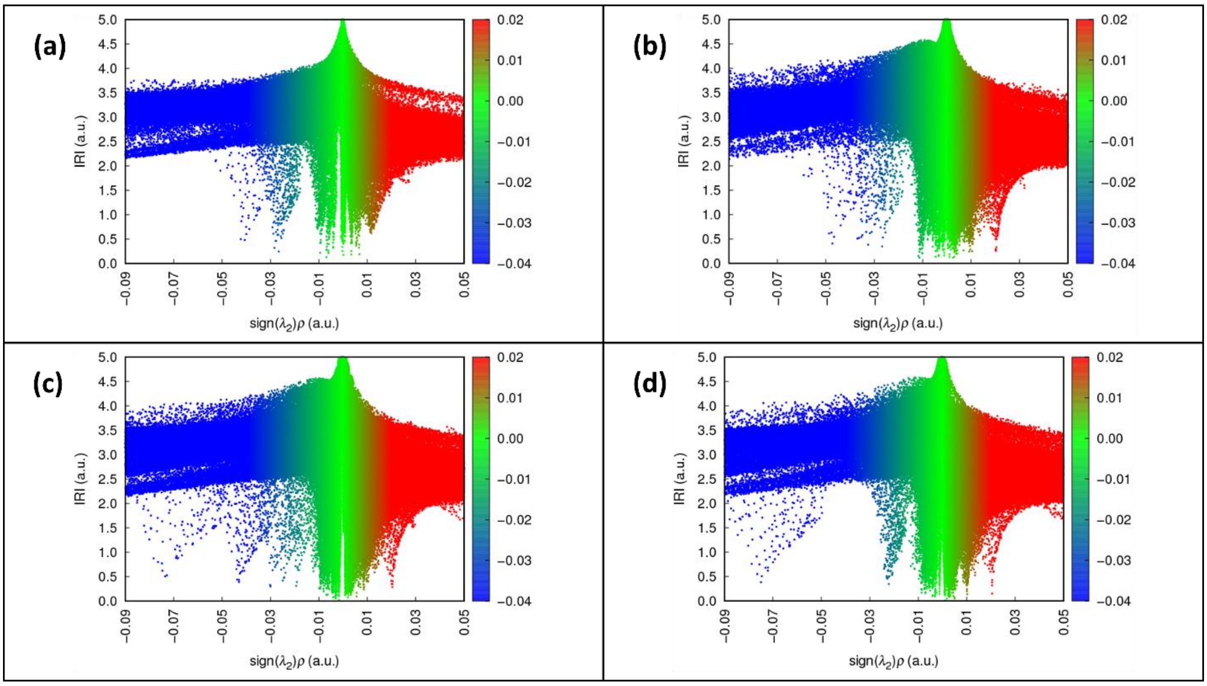

Figure S45. *Scatterplots of HB-interaction spheres of **1**-**3** (a), **2**-**3a** (b), the interaction sphere around the first distinctive **2** molecule in **2**-**3b** (c) and the same for the second distinctive **2** molecule in **2**-**3b** (d). Colors indicate occurring interaction types: blue corresponds to HBs, green to van-der-Waals interactions and red to intermolecular repulsion.*

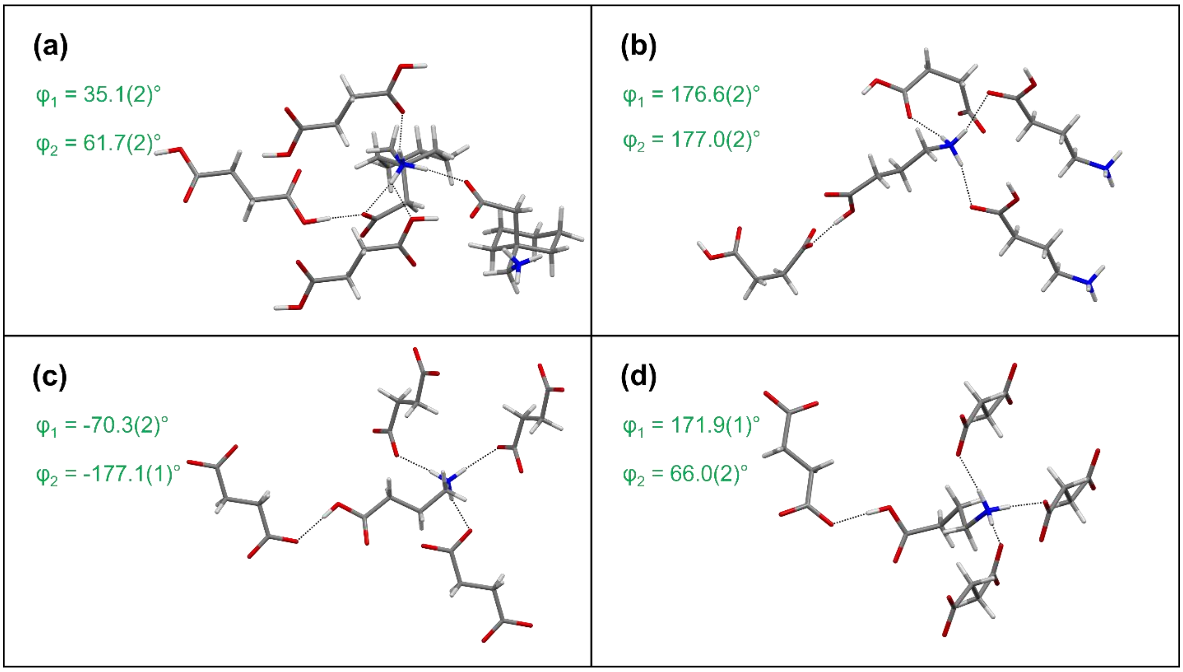

Figure S46. *Torsion angles and selected hydrogen bonds for (a) **2-4**, (b) **1-4b**, (c) first distinctive GABA molecule in **1-4b** and (d) second distinctive GABA molecule in **1-4a**. Hydrogen bonds are depicted as black dotted lines, torsion angles are only depicted as values for better visibility, oxygen atoms in red, nitrogen atoms in blue, carbon atoms in grey and hydrogen atoms in white.*

Table S25 *Overview on the strong, distinctive hydrogen bonds in the succinates of GABA and the gabapentin:succinic acid co-crystal. The proton acceptor distance, the donor acceptor distance, the bond angle and the binding energy calculated for charged HBs are shown.*

Compound	Interaction	H…A [Å]	D…A [Å]	D - H…A [°]	Ebond [kJmol-1]
**1-4a**	O1-H1…O5	1.62(3)	2.543(6)	173(2)	-84.68
	O3-H11…O7	1.72(3)	2.581(3)	178(2)	-64.86
	N1-H8…O5	1.84(2)	2.783(8)	173(2)	-52.26
	N2-H20…O8	1.88(2)	2.836(6)	163(2)	-51.29
	N2-H18…O7	1.87(2)	2.765(1)	165(2)	-49.47
	N1-H10…O6	1.90(2)	2.816(5)	173(2)	-43.73
	N2-H19…O8	1.94(3)	2.845(2)	171(7)	-41.51
	N1-H9…O6	1.98(2)	2.900(2)	172(8)	-41.14
**1-4b**	O1-H1…O3	1.52(3)	2.455(2)	175(4)	-104.67
	O5-H11…O4	1.78(4)	2.583(2)	163(3)	-52.74
	N1-H8…O2	1.95(3)	2.861(3)	161(3)	-38.57
	N1-H9…O2	2.02(3)	2.931(3)	166(2)	-33.56
	N1-H10…O6	2.05(3)	2.861(3)	156(2)	-30.40
**2-4**	O3-H18…O1	1.50(3)	2.522(0)	175(2)	-111.50
	N1-H7…O2	1.79(3)	2.734(3)	175(2)	-56.03
	N1-H6…O4	1.94(2)	2.776(0)	155(2)	-38.62
	N1-H5…O1	2.06(2)	2.811(2)	137(6)	-36.47
	N1-H5…O3	2.51(2)	3.036(7)	116(4)	-16.71

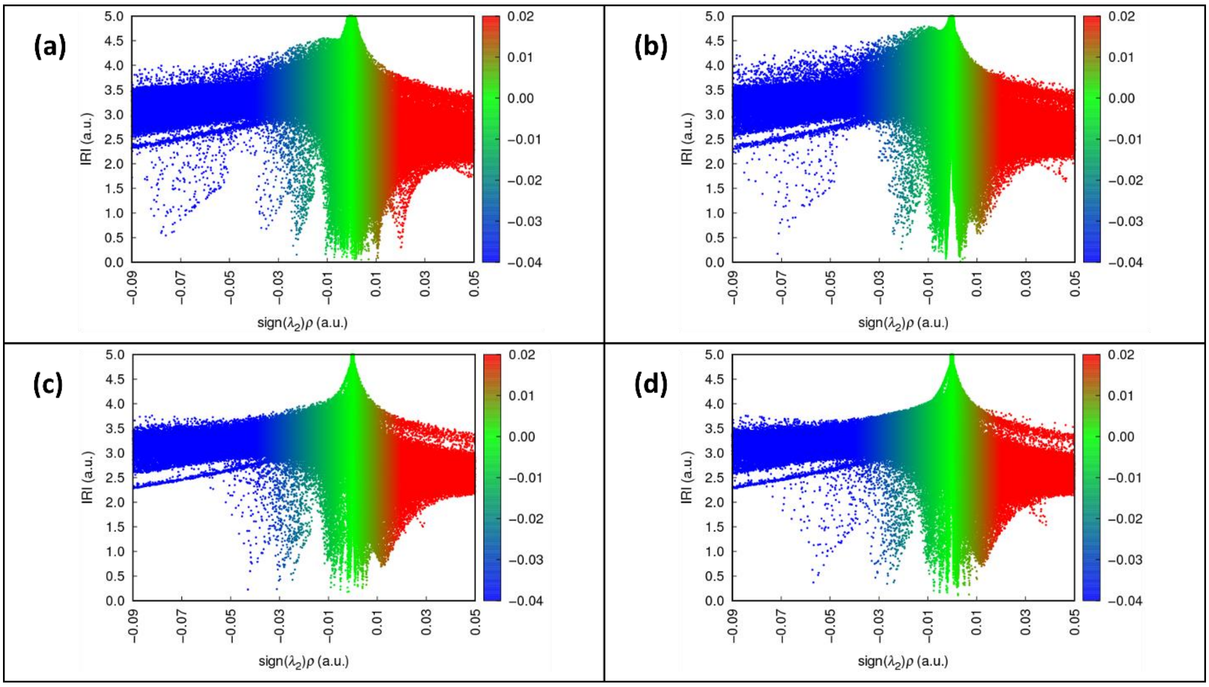

Figure S47. *Scatterplots of HB-interaction spheres of **2-4** (a), **1-4a** (b), the interaction sphere around the first distinctive **1** molecule in **1-4b** (c) and the same for second distinctive **1** molecule in **1-4b** (d). Colors indicate occurring interaction types: blue corresponds to HBs, green to van-der-Waals interactions and red to intermolecular repulsion.*
